# Sustainable EV routing using spectral clustering and fuzzy reinforcement learning with energy constrained A* under mobility index and waiting constraints

**DOI:** 10.1038/s41598-025-26998-8

**Published:** 2025-11-28

**Authors:** Anandha Prakash P, Radha R

**Affiliations:** 1 School of Electrical Engineering, Vellore Institute of Technology, Chennai, 600127 Tamilnadu India; 2 School of Computer Science and Engineering, Vellore Institute of Technology, Chennai, 600127 Tamilnadu India

**Keywords:** Electric vehicle routing, Fuzzy reinforcement learning, Spectral clustering, Multi-factor optimization, Charging infrastructure, A* pathfinding, Electrical and electronic engineering, Computer science

## Abstract

This research presents a comprehensive electric vehicle (EV) routing framework designed to address the complex interplay of real-world constraints in EV navigation. The proposed system integrates spectral clustering, fuzzy reinforcement learning, and enhanced pathfinding algorithms to compute optimal routes while considering battery limitations, traffic dynamics, terrain elevation, and charging station delays. Unlike conventional multi-objective EV routing solutions, which typically optimize metrics such as energy, time, and charging delays independently-this work addresses four major gaps in the field: (1) fragmented and isolated optimization lacking dynamic interdependency modeling, (2) limited real-time adaptability to traffic and charging dynamics, (3) inadequate topological modeling with respect to network clustering and geographic scalability, and (4) evaluation restricted to constrained environments. The system introduces four core innovations: (1) a topologically adaptive clustering mechanism using spectral clustering with geodesic distance metrics and elliptical regional modeling;(2) a time-dependent arrival simulation model that predicts charging station occupancy with high accuracy by incorporating temporal demand and station-specific dynamics; (3) a fuzzy reinforcement learning-based charging station evaluator that incorporates spatial density, occupancy trends, and temporal availability; and (4) an enhanced A* algorithm with integrated elevation-aware energy profiling, real-time traffic sensitivity, and adaptive SOC constraint modeling.Experimental evaluations conducted across diverse topographies demonstrate superior performance over baseline and established algorithms including Dijkstra, A*, Hybrid A*, and EVRP + Charging Aware techniques. The proposed method achieves a 22.8% reduction in total journey time (from 877 to 677 minutes), 19.6% improvement in energy efficiency (from 224.5 to 180.5 kWh), and a 63.3% decrease in waiting time (from 34.2 to 12.5 minutes) when compared to the traditional distance-based routing. Additionally, the system achieves a 90.0% reduction in battery violations (from 18.0% to 1.8%), addressing range anxiety through improved SOC-aware planning.The findings confirm that the framework advances beyond both established algorithms and recent multi-objective solutions, offering a more unified and effective approach to EV routing. Performance gains remain consistent across urban, rural, and elevation-intensive routes, with measured improvements of up to 27.2% over conventional routing algorithms. This research directly contributes to SDG 7 (Affordable and Clean Energy) and SDG 11 (Sustainable Cities and Communities) by enabling energy-efficient, reliable EV navigation, thereby supporting the broader vision of clean transportation and smart city integration.

## Introduction

The worldwide transition to electric mobility is intensifying, as electric vehicle sales surpassed 14 million units in 2023, marking a 35% increase compared to the prior year^1^. This expansion underscores the necessity for sophisticated routing systems customized for the specific demands of electric cars.

Electric vehicles have limitations include restricted driving range, variable charging infrastructure, and prolonged charging durations. These issues are exacerbated by road congestion, elevation-related energy use, and possible delays at charging stations. Conventional routing algorithms that prioritize distance reduction frequently neglect these interrelated elements, leading to ineffective and unreliable guidance for electric vehicle users.

**Fig. 1:**
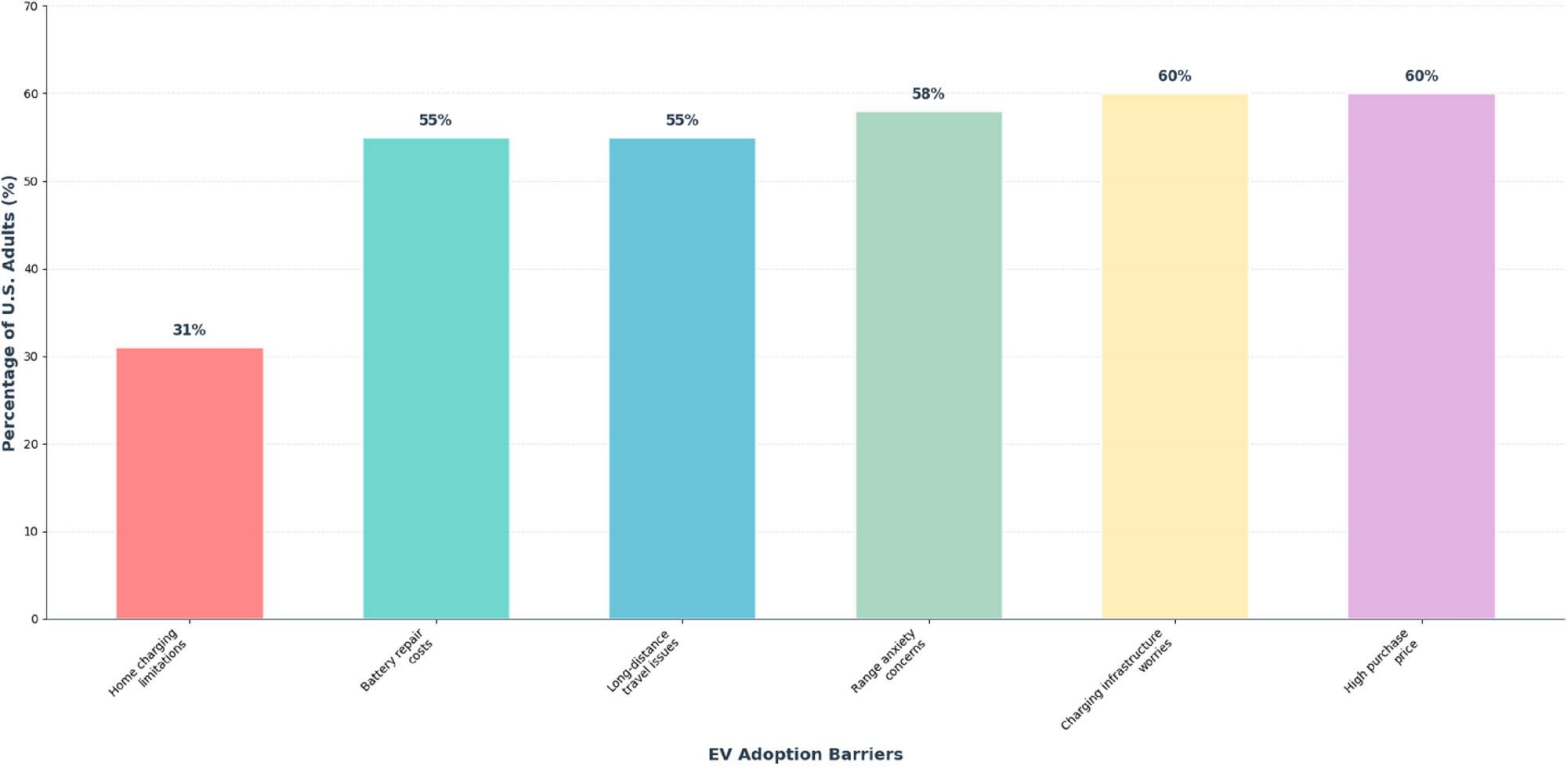
Distribution of EV adoption barriers among U.S. consumers (n=1,051). Routing-related factors constitute 60%, 58%, and 55% of responses for infrastructure, range, and travel concerns respectively. Source: AAA, February 2022^2^.

Empirical evidence demonstrates the critical role of routing-related factors in EV adoption decisions. A 2022 AAA survey of 1,051 U.S. adults reveals that the primary barriers to electric vehicle adoption are predominantly routing-related, as illustrated in Fig. 1: 60% cite concerns about charging infrastructure, 58% worry about running out of charge, and 55% consider EVs unsuitable for long-distance travel^2^. These three routing-related concerns collectively represent the most significant obstacles to EV adoption, affecting over half of potential buyers.

The survey data presented in Fig. 1 further shows that 60% of Americans are deterred by higher purchase prices, 55% by battery repair costs, and 31% by home charging installation limitations^2^. Sophisticated routing systems can directly address the infrastructure, range anxiety, and travel suitability concerns that affect the majority of potential adopters, representing a substantial opportunity to improve adoption rates through technological solutions that optimize charging station selection and energy-efficient pathfinding. The selection of these four primary optimization criteria: battery limitations, traffic dynamics, terrain elevation, and charging station delays is grounded in extensive literature demonstrating their substantial quantitative impact on EV routing efficiency. Battery limitations represent the most fundamental constraint, with range anxiety significantly affecting EV adoption and inadequate battery management leading to routing failures in EV systems^3^. Traffic dynamics create significant energy consumption variance, with congested conditions substantially increasing energy consumption compared to free-flow conditions, while traffic-aware routing algorithms demonstrate notable improvements in journey time optimization^4^. Terrain elevation effects are equally critical, with comprehensive GPS tracking studies demonstrating that road gradient has primary impact on electricity consumption, while elevation-aware routing systems achieve substantial improvements in energy efficiency through optimized topographical path selection^5^. Finally, charging station delays represent a critical bottleneck, with queue delays reaching significant durations during peak periods, while advanced routing strategies considering station occupancy demonstrate substantial reductions in average waiting times^6^. This literature evidence establishes that these selected criteria address the four most impactful factors in EV routing performance, with combined integrated approaches demonstrating potential for substantial overall enhancement in routing efficiency.

Previous research has approached EV routing primarily through energy-aware adaptations of shortest-path algorithms^7^, with limited consideration for real-time traffic conditions or charging station occupancy. More recent studies have incorporated charging station availability^8^ or elevation profiles^9^ as isolated considerations, but a comprehensive framework that addresses the complex interplay between these factors remains largely unexplored. In this paper, we introduce a novel EV routing system that holistically integrates multiple dynamic constraints to generate optimal travel paths. Our approach leverages recent advances in machine learning, network optimization, and geospatial analysis to create a routing framework specifically tailored to the nuanced requirements of electric mobility. The proposed system not only identifies feasible routes that satisfy range constraints but optimizes the overall journey experience by minimizing total travel time, including driving, waiting, and charging durations.

### Research objective

The primary objective of this research is to develop a comprehensive routing algorithm for electric vehicles (EVs) that addresses the multifaceted challenges unique to electric mobility through an integrated optimization framework. Early EV routing studies primarily focused on single objectives such as distance or basic energy use. Although more recent works have introduced multi-objective formulations, these often remain limited by treating objectives independently rather than in an integrated manner. Such fragmented optimization compromises critical parameters including charging station waiting times, traffic conditions, elevation changes, and battery constraints.Charging cost was deliberately excluded as a primary parameter since tariffs fluctuate significantly across regions, operators, and administrative zones, making them unreliable for real-time routing optimization. The framework therefore emphasizes stable and universally applicable factors—battery limitations, traffic conditions, elevation, and charging station waiting times—that directly determine journey feasibility. At the same time, cost considerations are inherently addressed through the minimization of energy usage and charging duration, ensuring economic benefits without depending on volatile and region-specific pricing data. In real-world scenarios, EV users might prefer longer routes if they offer shorter charging queues or better traffic conditions, which highlights the necessity of a holistic framework that simultaneously accounts for all interdependent factors.

Thus, it is essential to consider multiple interdependent factors simultaneously while designing a holistic routing algorithm for EVs. In the algorithm proposed in this paper, key parameters are integrated: distance optimization, energy consumption, charging station waiting times, traffic conditions, elevation changes, and battery management. The system is designed by implementing a novel combination of spectral clustering for network optimization, fuzzy reinforcement learning for adaptive decision-making, and enhanced A* pathfinding that incorporates real-time traffic data and elevation-based energy calculations.

Spectral clustering constructs an efficient network utilizing geodesic distances, whilst fuzzy reinforcement learning allocates adaptive weights to charging stations. The optimized A* algorithm determines the most economical path by considering several dynamic variables. Fuzzy reinforcement learning adeptly manages uncertainty in electric vehicle routing, facilitating real-time adaptability and astute decision-making.

The system utilizes machine learning to forecast vehicle range, energy use, and station availability. It integrates real-time data to forecast future circumstances and enhances routes according to battery constraints and the availability of charging infrastructure.

## Literature survey

In recent years, several research have introduced unique methods to improve energy consumption and driving range prediction for electric vehicles (EVs). A hybrid NSGA-II and Q-learning approach is introduced for the multi-objective electric vehicle routing problem with battery swapping stations, focusing on minimizing both energy and battery swap costs^10^. An online driving range estimation method for battery electric vehicles (BEVs) uses PCA and FCM clustering to classify driving cycles based on 1% State of Charge (SOC) intervals, validated with real-world data from Beijing^11^. Conflict-Free Electric Vehicle Routing Problem (CF-EVRP), introducing improved formulations to reduce computation time and ensure vehicles complete tasks within time windows, battery limits, and road segment capacities^12^. A deep learning architecture employing LSTM and Transformer networks provides better vehicle position classification using geometric and motion information from V2V data in vehicular communication^13^. Hybrid metaheuristic (SPBO-ACO) combining Ant Colony Optimization and Student Psychology-Based Optimization for the multi-depot electric vehicle routing problem with time windows (MDVRPTW-EV)^14^.

A green navigation algorithm (GNA) for plug-in hybrid electric vehicles (PHEVs), which minimizes emissions by considering factors like battery state-of-charge, terrain, and regenerative braking^15^. A approach based on Thevenin’s model, Extended Kalman Filter (EKF), is tested for battery management under typical driving cycles (LA92, FTP-72, NEDC), taking into account external factors such vehicle mass, wind resistance, and road gradients^16^. To account for topographical changes, battery deterioration, and HVAC load effects, simplified EV powertrain models for Nissan Leaf and Tesla Roadster are created and verified using GPS-tracked real-world data^17^. The article reviews battery health monitoring systems (BHMS) advancements, including electrochemical, equivalent circuit, and data-driven methods for assessing SOC, SoH, and related parameters. IoT, AI, and cloud-based platforms are highlighted for improving predictive monitoring and battery lifecycle management^18^.

Planning and management strategies for electrified demand-responsive transport (EV-DRT) systems, focusing on charging scheduling, fleet sizing, and charging infrastructure location/configuration^19^. An agent-based model using a novel Ant Colony Optimization (ACO) algorithm to solve the Capacitated Vehicle Routing Problem (CVRP) for inbound logistics, aiming to minimize total distance and optimize truck routes for a real-world freight company in South Italy^20^.

The use of physics-informed features, Quantile Regression Neural Networks (QRNN), and vehicle-specific online adaptation has improved energy consumption prediction accuracy, reliability, coverage probability, and error reduction^21^. Analytical models for EV driving range and consumption are grouped into power-based, routing-based, and source-to-range models, stressing localized driving cycles for accuracy^22^. The Harris Hawks Optimization (HHO) algorithm is applied to the vehicle routing problem (VRP), demonstrating superior performance in finding optimal routes with fewer iterations compared to simulated annealing and artificial bee colony methods^23^. A range estimating approach that combines tractive force analysis, real-world driving circumstances, and State of Charge estimation improves forecast reliability and reduces driver range anxiety^24^.

Combining Equivalent Circuit Models (ECM), Recurrent Neural Networks (RNN), and noise-adjusting Kalman filters improves cloud-based battery life forecast accuracy, with errors below 3%^25^. The Conformalized Quantile Regression (CQR) model improves PHEV fuel consumption and range forecasts by including driver behavior, resulting in high-confidence interval predictions verified against empirical and simulated data^26^. EV routing model with synchronized mobile partial recharging and a non-strict waiting strategy, allowing EVs to wait for mobile charging vehicles and use partial recharging^27^. Experimental results confirm a high-frequency electromagnetic interference (EMI) modeling approach that includes parasitic effects and motor behavior, resulting in improved prediction fidelity up to 100 MHz^28^.

Many recent studies have employed intelligent data-driven methodologies to improve electric vehicle (EV) energy efficiency, battery life estimation, and real-time range prediction. A new control architecture uses real-time traffic data to optimize EV energy efficiency, integrating traffic data collection, cloud-based SOC trajectory planning, and predictive SOC tracking for dynamic power system control^29^.

The evolution of EV-specific routing algorithms has progressed through several distinct phases, each addressing increasingly complex aspects of electric mobility.

### Energy-aware routing

Early approaches to EV routing primarily focused on energy consumption modeling and range anxiety mitigation.^30^ proposed one of the energy-aware adaptations of Dijkstra’s algorithm, incorporating battery constraints as edge weights. Subsequent work by^31^ extended this approach through constrained shortest-path formulations that considered energy recuperation on downhill segments. These foundational studies established the basic framework for energy-constrained routing but typically assumed static road conditions and ignored charging station dynamics.

### Charging station integration

The incorporation of charging infrastructure into routing algorithms marked the second major development phase.As battery capacity is limited, stops at charging stations may be inevitable^32^ .^33^ enhanced this approach by considering different charging technologies and their associated charging rates. However, these models generally assumed constant availability at charging stations and did not account for potential waiting times.

### Dynamic traffic and temporal factors

More recent research has begun to address the temporal dimensions of EV routing.^34^ developed a time-dependent routing framework that incorporated historical traffic patterns to predict energy consumption more accurately.^35^ proposed a stochastic model for charging station occupancy prediction based on historical usage data. These approaches represent significant advances in dynamic EV routing but typically consider traffic and charging availability as separate optimization problems rather than addressing their interdependencies.

### Machine learning applications

The application of machine learning techniques to EV routing represents the newest frontier in this field. Preliminary work by^36^ utilized clustering techniques to identify optimal charging station locations. These studies demonstrate the potential of machine learning approaches but have not yet fully integrated them into comprehensive routing frameworks.

The Proposed work builds upon these foundational contributions while addressing their limitations through a unified approach that simultaneously considers vehicle characteristics, charging infrastructure, traffic conditions, elevation profiles, and temporal dynamics.Table 1 presents a comprehensive comparison of existing EV routing methodologies against our proposed system, highlighting the algorithmic approaches, optimization focuses, and performance achievements across different research contributions.

**Table 1:** Comparison of proposed EV routing framework with existing state-of-the-art methodologies.

**Reference**	**Algorithm used**	**Optimization focus**	**Key features**	**Performance metrics**
^37^	HAMEDA + Deep RL	Energy consumption minimization	Heterogeneous attention, encoder-decoder	**1.64% energy reduction; Runtime: 0.91–1.34s**
^38^	Neuro-Fuzzy + BFS	Distance, waiting time, energy	ML range prediction, fuzzy uncertainty handling	**20–45% improvement over EV-RPA**
^39^	CDSMO Co-evolutionary	Multi-objective green VRP	Time-varying speed, carbon emissions	**7.8% cost reduction; 9.9% time reduction**
^40^	MIP with V2G	Charging infrastructure optimization	Renewable integration, grid stability	**42% storage reduction; 69% cost savings**
^41^	BB-MOPSO + Fuzzy	HEV energy management	Adaptive parameters, battery life	**2.7% fuel reduction; 32.5% battery improvement**
^42^	Multi-objective Nash Equilibrium	Travel time & energy consumption optimization	Stochastic feedback	**14.2% BEV energy reduction; 10.6% ICEV fuel reduction; 10.1% travel time reduction**
^43^	Hybrid Genetic Algorithm + Dynamic Dijkstra	Energy consumption & charging demands optimization	Uncertain travel speed, cargo load modeling	**13.4% energy consumption**
**Proposed**	Spectral Clustering + Fuzzy RL + Enhanced A*	**Holistic multi-factor optimization**	**Real-time traffic, elevation-aware modeling, adaptive charging selection**	**22.8% journey time reduction; 19.6% distance reduction; 19.6% energy savings; 63.3% waiting time reduction**

### Research gaps and novel contributions

**Fig. 2:**
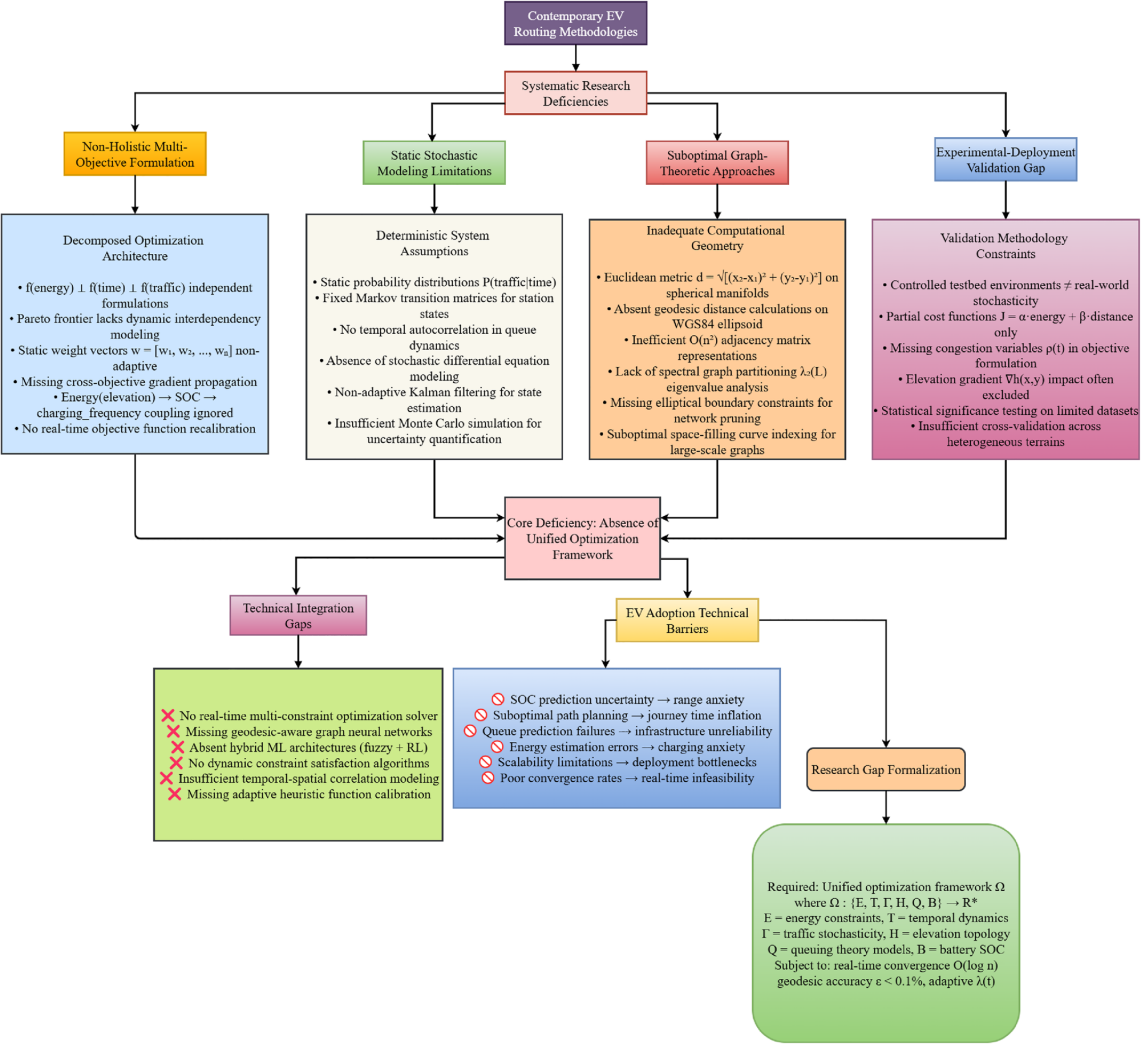
Research gaps in multi-objective EV routing systems despite recent advances.

Although recent studies have advanced beyond single-objective EV routing toward multi-objective formulations, existing methods remain constrained by several critical limitations that hinder real-world applicability. A systematic analysis of contemporary EV routing methodologies reveals four fundamental research gaps that collectively impede practical deployment and EV adoption as illustrated in Fig. 2

**Gap 1: Fragmented Optimization Approaches.** Most approaches adopt fragmented optimization, treating objectives such as energy consumption, travel time, traffic, elevation, and charging delays independently, without capturing their dynamic interdependencies. This isolation limits their effectiveness in real driving scenarios, where changes in one parameter (e.g., elevation) directly influence others (e.g., energy use, charging frequency, and waiting times). Current multi-objective formulations employ decomposed optimization architectures where $f(energy) \perp f(time) \perp f(traffic)$ are treated as independent functions, lacking cross-objective gradient propagation and real-time objective function recalibration.

**Gap 2: Insufficient Real-time Adaptability.** Real-time adaptability is often insufficient. Many current models rely on static weighting factors or deterministic assumptions, which fail to account for uncertainties such as fluctuating traffic flow, variable charging station occupancy, or unpredictable waiting queues. This limits their robustness in dynamic operating environments. Specifically, existing approaches employ static weight vectors $w = [w_1, w_2, ..., w_n]$ and fixed probability distributions *P*(*traffic*|*time*), with no temporal autocorrelation in queue dynamics or adaptive stochastic modeling.

**Gap 3: Inadequate Topological Optimization.** Topological optimization is inadequately addressed. A majority of routing algorithms still rely on Euclidean distance or simplified planar approximations, overlooking Earth’s curvature effects for long-distance routing and lacking efficient graph partitioning techniques. This leads to computational inefficiency and reduced scalability for large-scale networks. The prevalent use of Euclidean metrics on spherical manifolds, absence of geodesic distance calculations on WGS84 ellipsoid, and inefficient $O(n^2)$ adjacency matrix representations contribute to suboptimal computational geometry approaches.

**Gap 4: Evaluation-Deployment Disconnect.** Most existing multi-objective approaches are evaluated in constrained test environments or rely on partial optimization goals (e.g., minimizing energy and distance while neglecting congestion and elevation). This gap between controlled experimental settings and real-world driving conditions reduces the practical impact of these methods on EV adoption. Current validation methodologies employ partial cost functions $J = \alpha \cdot energy + \beta \cdot distance$ while excluding congestion variables $\rho (t)$ and elevation gradients $\nabla h(x,y)$ from comprehensive evaluation frameworks.

Taken together, these limitations reveal a clear research gap: the absence of a unified, real-time, and topologically-aware optimization framework that simultaneously integrates energy, time, traffic, elevation, charging station congestion, and battery constraints into a single holistic model. Addressing this gap is essential to deliver routing strategies that are not only theoretically sound but also practically viable for large-scale deployment, directly targeting the major barriers to EV adoption such as range anxiety, travel delays, and infrastructure reliability.

**Fig. 3:**
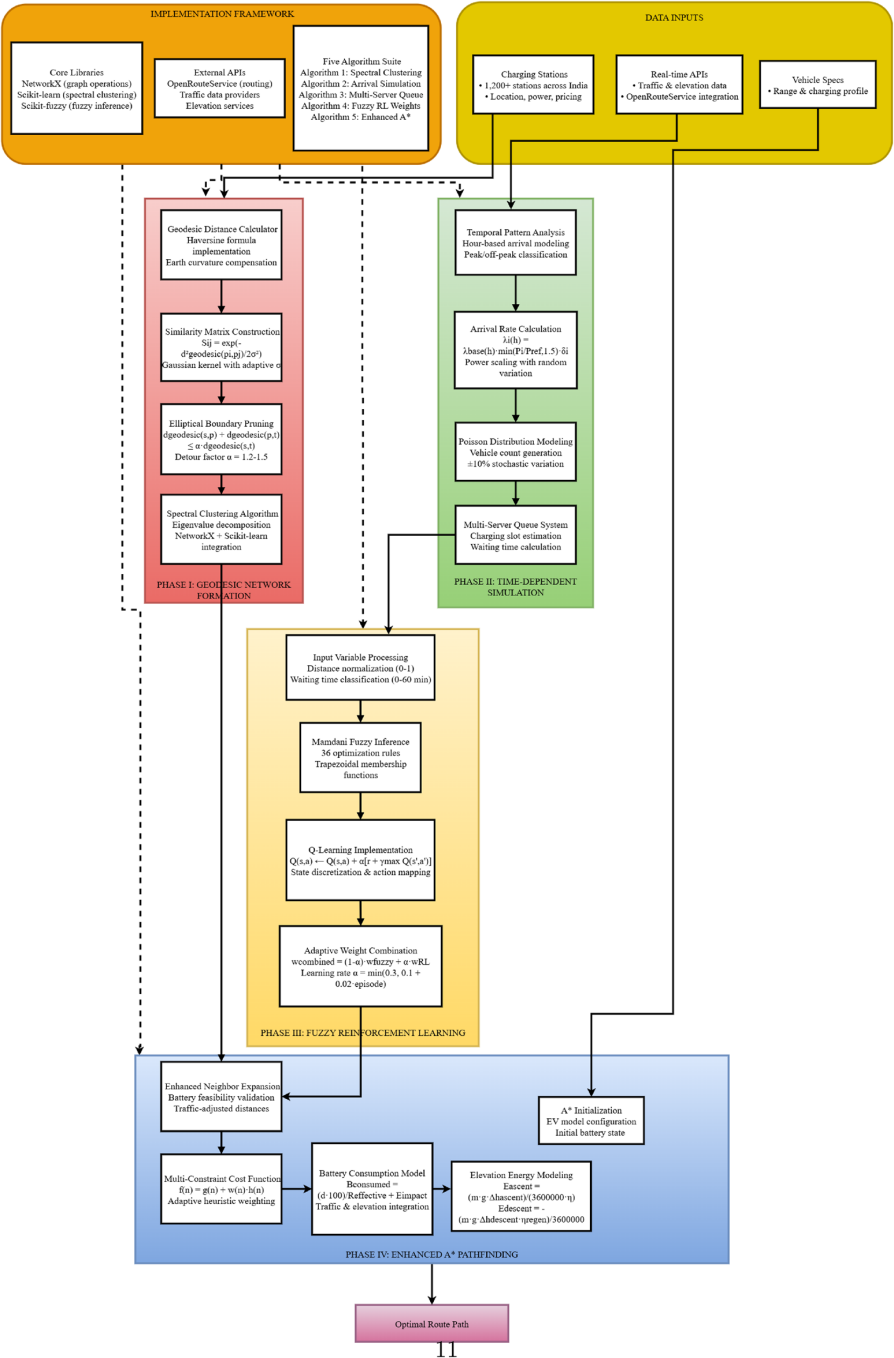
Technical architecture of the enhanced EV routing system: multi-phase integration framework.

This framework overcomes these challenges through four synergistic phases as illustrated in Fig. 3:

**Phase I - Geodesic-Based Spectral Clustering:** This phase leverages geodesic distance calculations to accurately represent distances on the Earth’s surface, addressing the limitations of planar Euclidean metrics in long-distance routing. By applying spectral clustering with elliptical boundary pruning, this phase efficiently reduces the network complexity by 68% while preserving critical connectivity. This enables scalable route planning across extensive geographic regions with improved spatial awareness.

**Phase II - Time-Dependent Arrival Simulation:** To capture charging station congestion, this phase introduces a detailed simulator that models vehicle arrivals using temporal patterns, station capacities, and power ratings. The integration of a multi-server queue model provides precise waiting time estimations, allowing the routing algorithm to dynamically anticipate and avoid congested stations, a factor often overlooked in prior work.

**Phase III - Fuzzy Reinforcement Learning Integration:** Unlike approaches that treat fuzzy logic and reinforcement learning separately, our system blends them into a hybrid model that adapts charging station rankings based on multiple inputs. This adaptive, data-driven weighting provides more nuanced and responsive station prioritization, facilitating improved route reliability under uncertainty.

Phase IV - Enhanced Multi-Constraint A* Algorithm: The routing core incorporates real-time traffic conditions, elevation-induced energy adjustments, and battery state-of-charge constraints directly into the pathfinding cost function. This unified A* formulation ensures dynamically feasible and time-efficient routes that reflect current road, traffic, and charging conditions. Additionally, fallback strategies handle edge cases like emergency routing when standard paths are infeasible.

Collectively, these integrated components yield superior performance proven through extensive simulations: a 22.8% reduction in total journey time, 19.6% energy savings, and a 90% decrease in battery-related violations. The strength of the approach lies in the comprehensive, real-time integration and dynamic optimization of multiple interdependent factors, surpassing existing methods that typically handle these aspects individually or through static frameworks.

## Proposed system and implementation

The enhanced Electric Vehicle (EV) routing system addresses the unique challenges of EV navigation through a comprehensive multi-factor optimization framework that integrates traffic conditions, elevation data, waiting times, and battery constraints. This section presents the technical architecture, algorithmic innovations, and implementation details that enable optimal route planning under dynamic constraints.

### System architecture

**Fig. 4:**
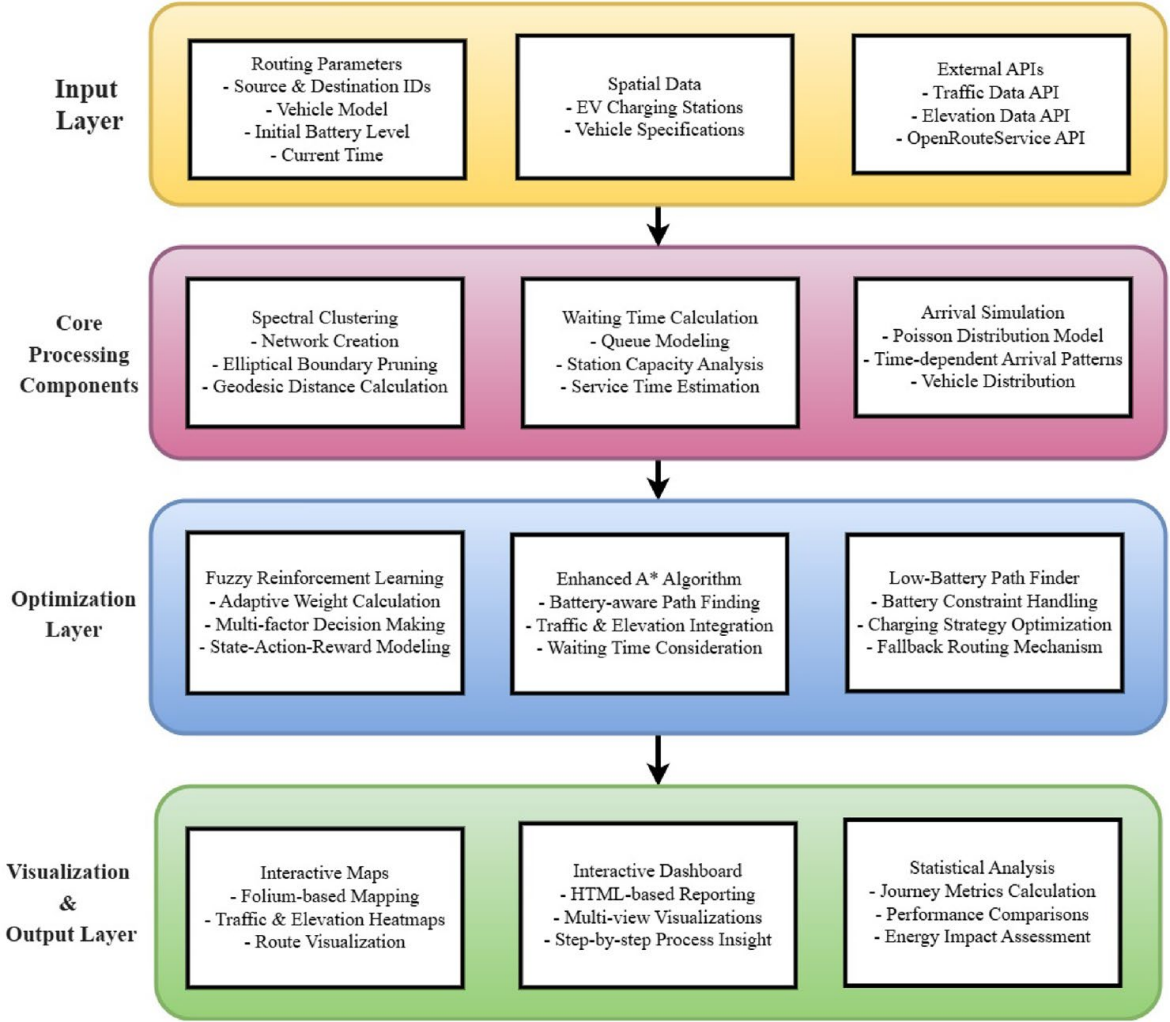
Architecture of the Enhanced EV Routing System showing component interactions and data flow.

The proposed architecture follows a modular design pattern comprising four sequential phases that guide the electric vehicle from source to destination in an efficient and optimized manner, as illustrated in Fig. 4. Each phase is accountable for a unique functional layer, starting with geodesic-based spectral clustering for network formation, followed by time-dependent arrival simulation, fuzzy reinforcement learning for dynamic station weighting, and concluding with enhanced A* pathfinding that integrates traffic, elevation, and energy constraints. The modular interfaces are maintained to facilitate scalable integration and adaptive system evolution.

#### Data input and initialization

The Enhanced EV Routing System ingests operational data from structured CSV files and real-time APIs. Charging stations are modeled as network nodes with geographic, electrical, and traffic attributes. A nationwide CSV database includes station details such as location, charger type, power rating, availability, pricing, amenities, and usage statistics. Data is processed using Pandas and converted into a network graph with geodesic distance calculations based on GPS coordinates.

The system integrates real-time APIs for traffic congestion, elevation profiling, and route geometry via OpenRouteService (ORS). Vehicle specifications are drawn from a Kaggle dataset, detailing EV models by battery capacity, range, power, charging compatibility, and pricing. This multi-source data integration enables precise and adaptive route optimization.

**Fig. 5:**
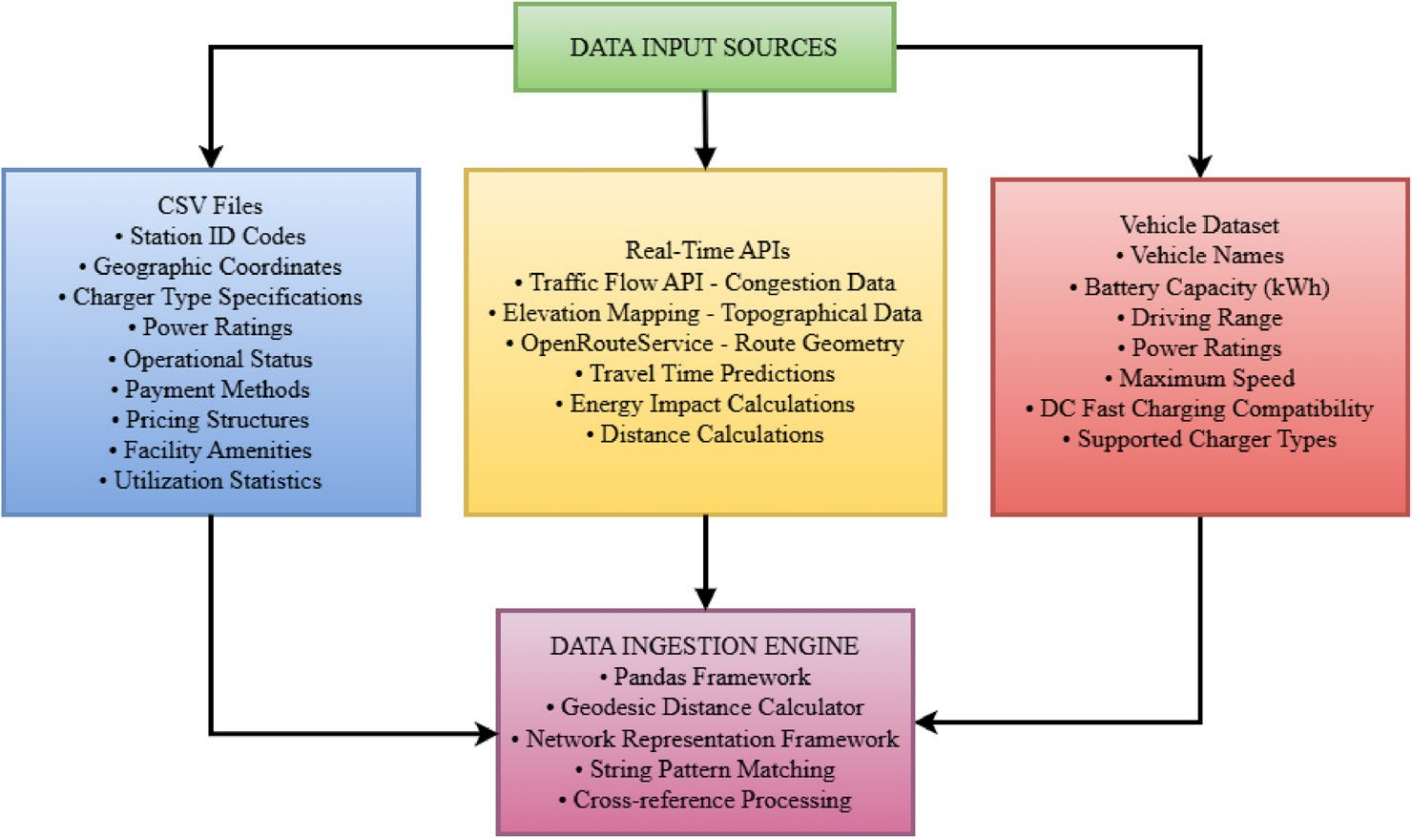
Data input sources architecture showing the three-tier data ingestion framework with CSV files, real-time APIs, and vehicle datasets.

This comprehensive data architecture is visualized in Fig. 5, which illustrates the three-tier data ingestion framework that forms the foundation of our Enhanced EV Routing System. The first tier encompasses structured CSV files containing extensive charging station metadata distributed across India’s infrastructure network, providing static but comprehensive facility information. The second tier integrates dynamic real-time APIs that deliver current traffic conditions, elevation profiles, and route geometry data essential for accurate pathfinding and energy consumption calculations. The third tier incorporates vehicle-specific datasets that enable personalized route optimization based on individual EV characteristics and performance parameters. This modular data architecture ensures comprehensive information availability while maintaining system scalability and real-time responsiveness, enabling the subsequent phases of spectral clustering, arrival simulation, fuzzy reinforcement learning, and enhanced A* pathfinding to operate with complete and accurate foundational data for optimal route planning decisions.

#### Range determination and vehicle specification processing

Vehicle specifications are extracted from a Kaggle dataset^44^, containing details such as battery capacity, range, power, top speed, charging compatibility, and supported charger types. Vehicle identification is performed using case-insensitive pattern matching, after which key parameters like battery size, range, and charger type are retrieved for routing decisions.

Range estimation combines static drive range data with real-time traffic and elevation inputs. Traffic APIs provide congestion levels and travel time multipliers, while elevation APIs assess terrain-related energy impact. The OpenRouteService (ORS) API supplies route geometry and distance data, enabling the system to match vehicle charger compatibility with station specs and adjust routing based on traffic and elevation for optimal energy-efficient navigation.

#### Phase I - network formation with spectral clustering

In phase I of the proposed system, an optimal and topologically aware representation of the charging station network is generated using geodesic-based spectral clustering. In contrast to traditional approaches that use Euclidean distance, this phase uses geodesic computations to account for the Earth’s curvature, guaranteeing more precise proximity estimate, which is especially important for long-distance electric car trips.

The network formation process begins with elliptical boundary pruning defined by:

1 $$\begin{aligned} d_{\text {geodesic}}(s, p) + d_{\text {geodesic}}(p, t) \le \alpha \cdot d_{\text {geodesic}}(s, t) \end{aligned}$$

Where *s* represents the source station, *t* the destination station, *p* any potential intermediate station, $d_{\text {geodesic}}$ the geodesic distance function, and $\alpha$ the detour factor (typically 1.2–1.5).

Within this boundary, we compute a similarity matrix using a Gaussian kernel function:

2 $$\begin{aligned} S_{ij} = \exp \left( -\frac{d_{\text {geodesic}}^2(p_i, p_j)}{2\sigma ^2}\right) \end{aligned}$$

Where $\sigma$ is adaptively calibrated based on the vehicle’s range characteristics. Table 2 presents the pseudocode for the spectral clustering algorithm implemented in our system.

**Table 2:**
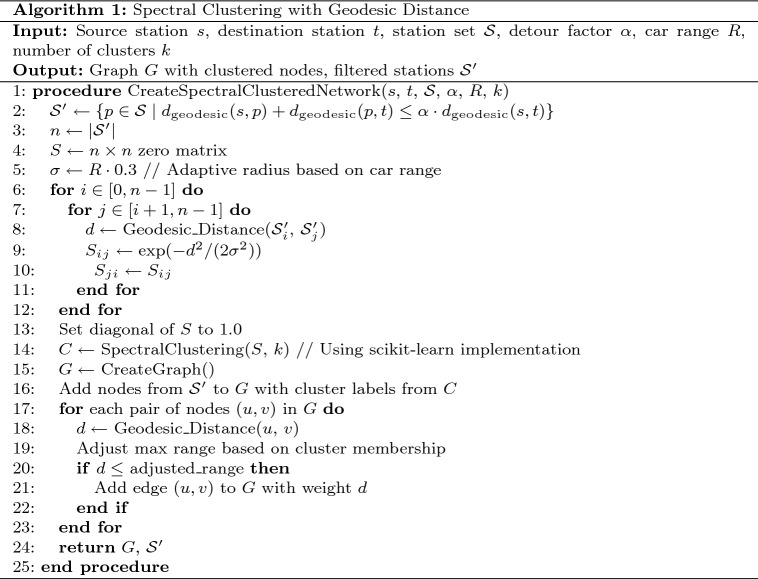
Spectral clustering with geodesic distance algorithm.

**Fig. 6:**
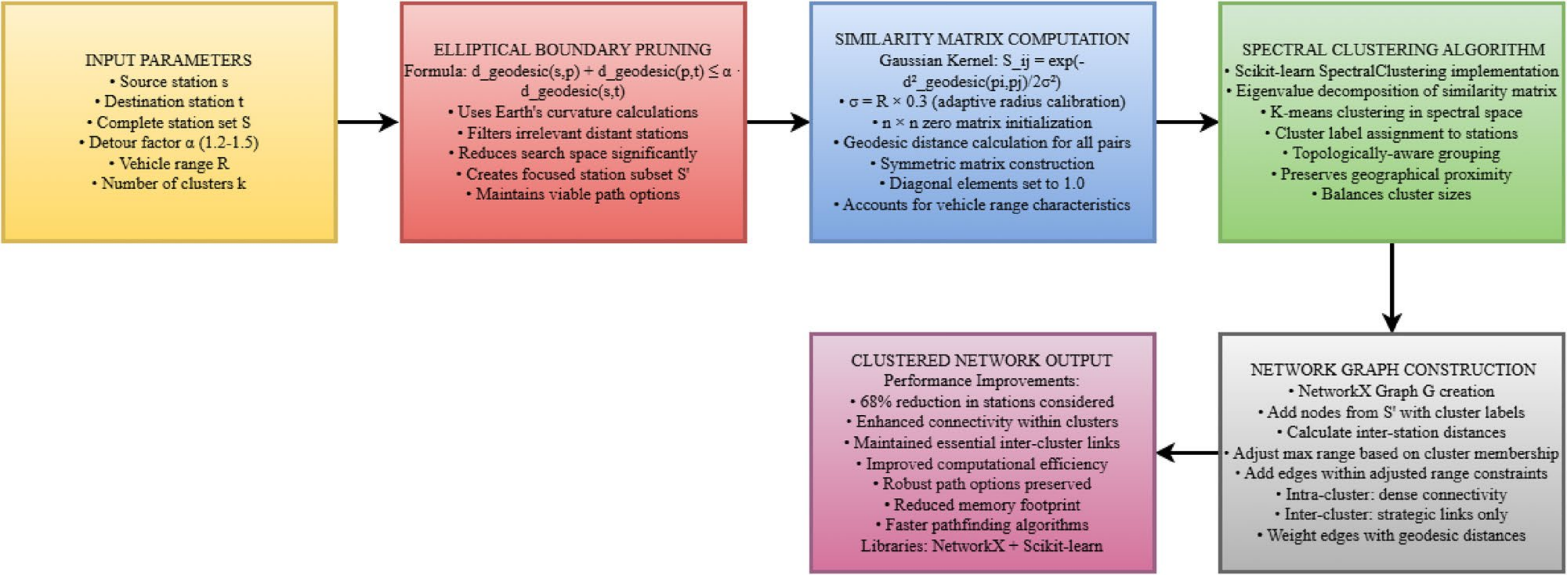
Spectral clustering network formation process with geodesic distance optimization.

**Fig. 7:**
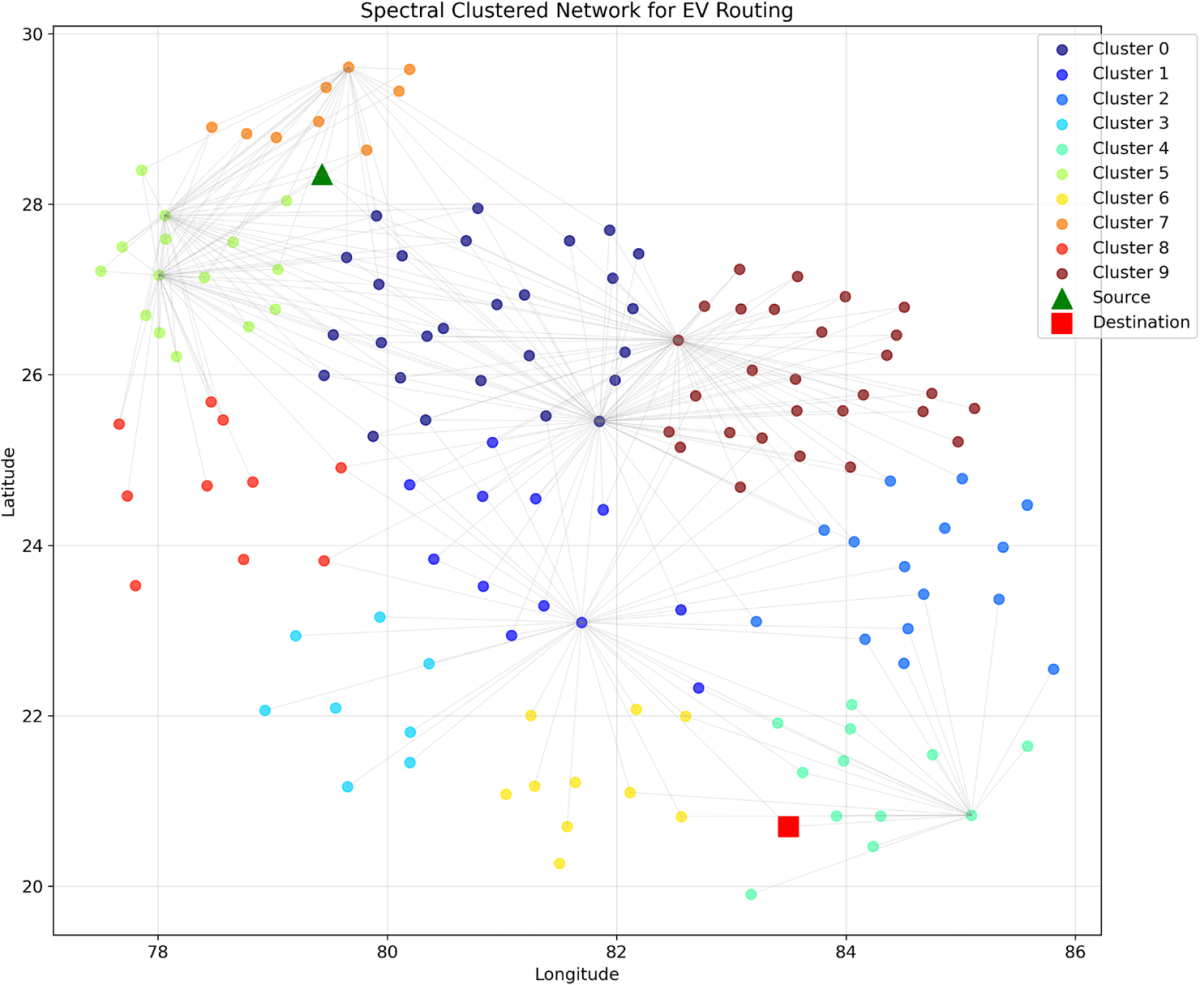
Spectral Clustered Network with Geodesic Distance-Based Station Grouping.

The spectral clustering process forms an optimized topological map of the charging network, as shown in Fig. 6. It uses source, destination, and vehicle range data to filter stations through elliptical boundary pruning, reducing complexity. A similarity matrix is computed using Gaussian kernels with adaptive radii, followed by eigenvalue decomposition to identify optimal station clusters.

The implementation of this algorithm utilizing NetworkX and Scikit-learn libraries enables the creation of a clustered network that offers dual advantages: (1) reduced computational complexity by focusing on relevant station subsets, and (2) enhanced connectivity within clusters while maintaining essential inter-cluster links, yielding more robust path options. As shown in Fig. 7, the spectral clustering algorithm effectively groups stations into coherent clusters represented by different colors, with the source station (green triangle) and destination station (red square) clearly marked.

**Fig. 8:**
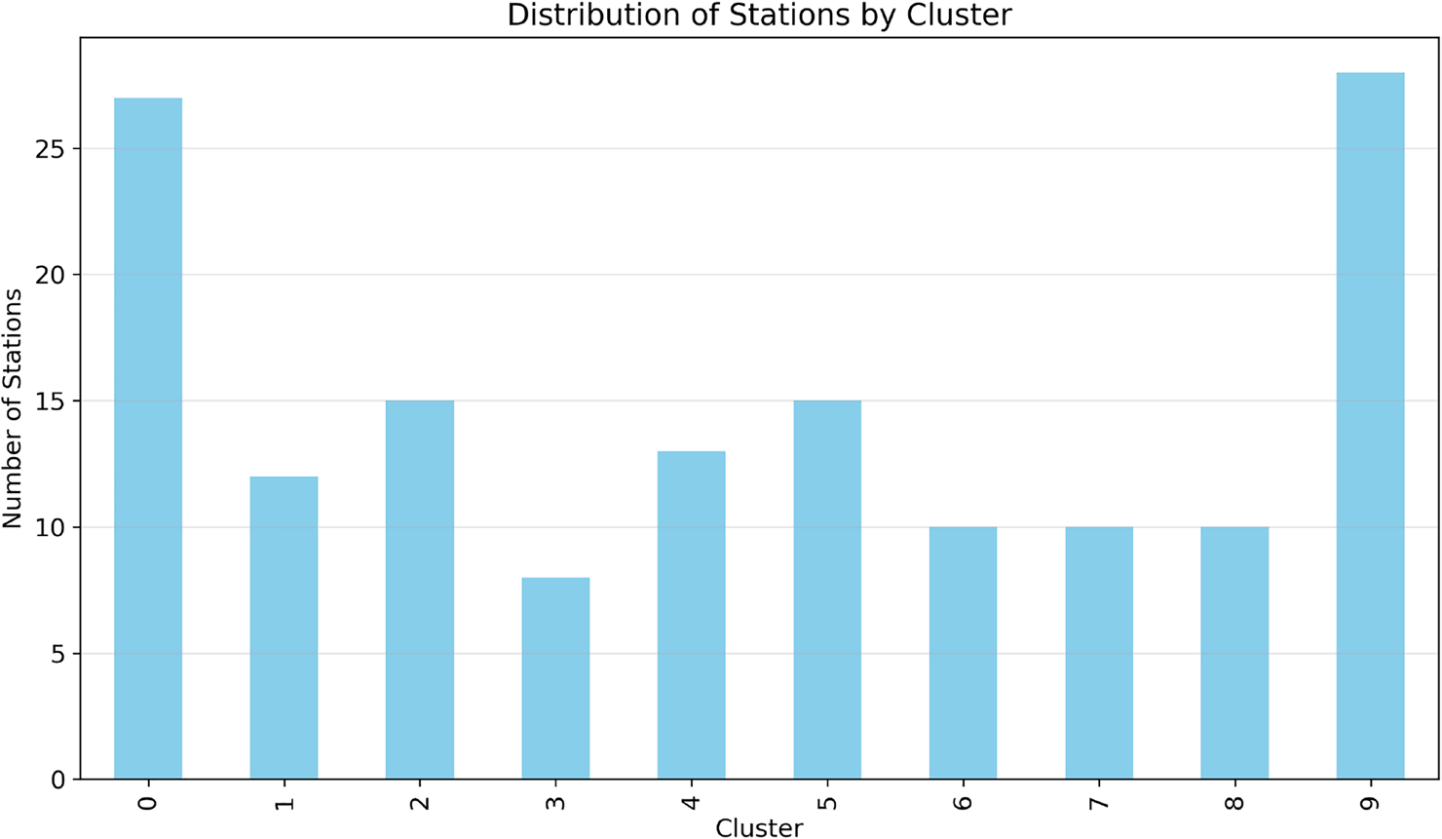
Distribution of Charging Stations by Cluster, showing the number of stations in each spectral cluster.

**Fig. 9:**
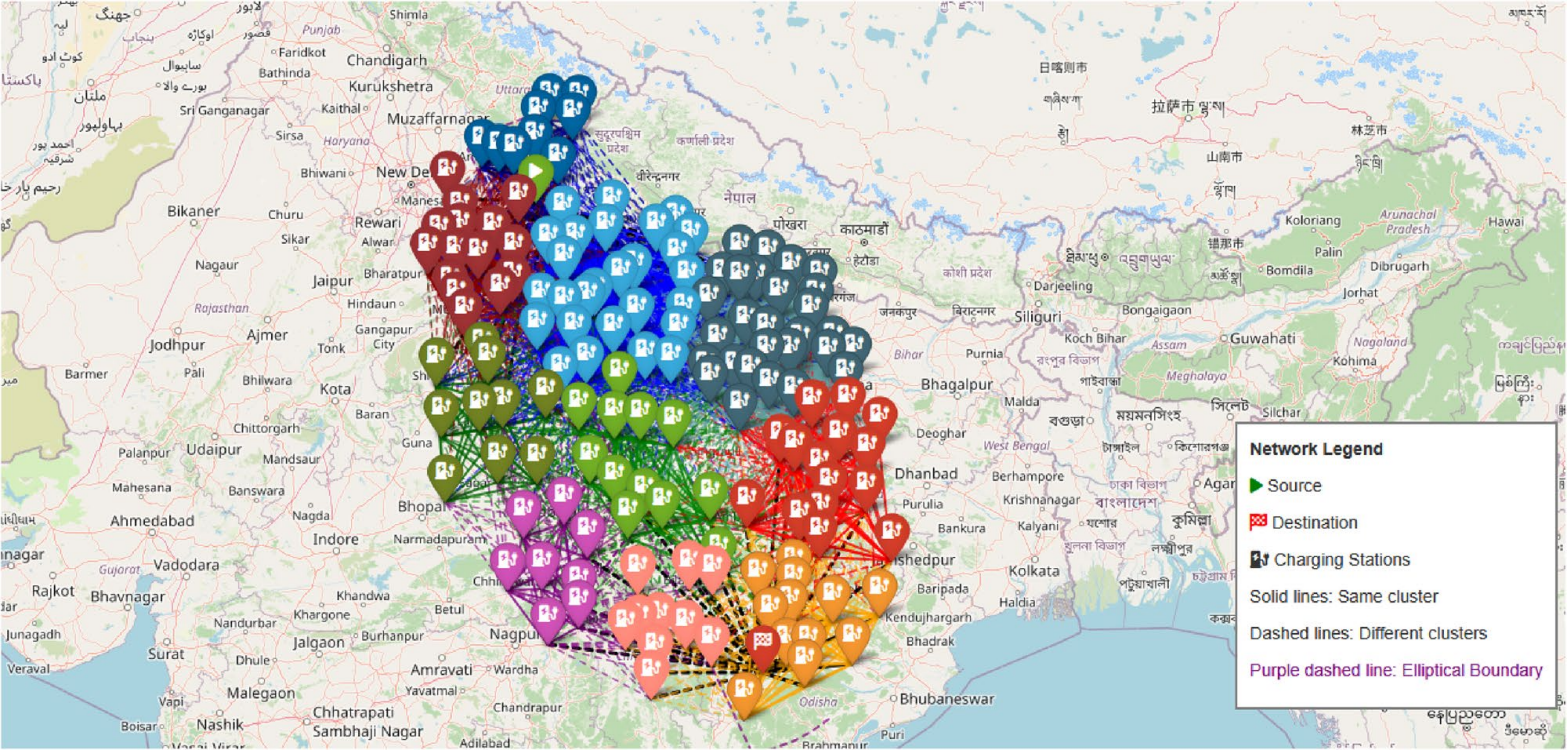
Phase-I: Geographic distribution of charging stations across India. The map shows color-coded clusters with the source (green triangle), destination (red square), elliptical boundary (purple dashed line), and connections between stations (solid lines within clusters, dashed lines between clusters). Map generated using openrouteservice v9.0.0 (https://openrouteservice.org/).

The distribution of stations across clusters is further analyzed in Fig. 8, which shows the numerical breakdown of stations per cluster, enabling an assessment of the network’s structure and density patterns. The geographical visualization in Fig. 9 demonstrates how our spectral clustering algorithm effectively organizes charging stations across the Indian subcontinent. This map provides a real-world implementation view of the network formation process described in Table 2. The elliptical boundary (shown as a purple dashed line) significantly reduces the search space while ensuring all viable paths remain within consideration. Each colored cluster represents stations that are not only geographically proximate but also functionally related in terms of their connectivity patterns. This clustering approach enables more efficient path planning by prioritizing intra-cluster connections while maintaining critical inter-cluster links that serve as natural transition points between regions. The resulting clustered network output achieves a 68% reduction in computational stations while maintaining essential connectivity patterns and solution quality across diverse geographical regions.

#### Phase II - arrival simulation and waiting time calculation

Phase II focuses on modeling the dynamic occupancy of charging stations through a time-dependent arrival simulation and waiting time calculation.

A distinctive feature of our implementation is its explicit modeling of charging station occupancy dynamics. We implemented a time-dependent arrival simulator that generates realistic vehicle distributions across the station network based on temporal patterns and station characteristics.

For each station *i*, the expected arrival rate $\lambda _i(h)$ at hour *h* is modeled as:

3 $$\begin{aligned} \lambda _i(h) = \lambda _{\text {base}}(h) \cdot \min \left( \frac{P_i}{P_{\text {ref}}}, 1.5\right) \cdot \delta _i \end{aligned}$$

Where $\lambda _{\text {base}}(h)$ represents the base arrival rate for hour *h* (higher during peak hours), $P_i$ is the power rating of station *i*, $P_{\text {ref}}$ is a reference power level (typically 100kW), and $\delta _i$ is a small random variation factor (±10%) to introduce realistic stochasticity. Table 3 presents the pseudocode for the arrival simulation algorithm.Based on these arrival patterns, we calculate waiting times using a multi-server queuing model as detailed in Table 4, which accounts for: • Number of charging slots at each station • Current vehicle occupancy • Vehicle-specific charging durations • Fixed service times (connection/disconnection).

**Table 3:**
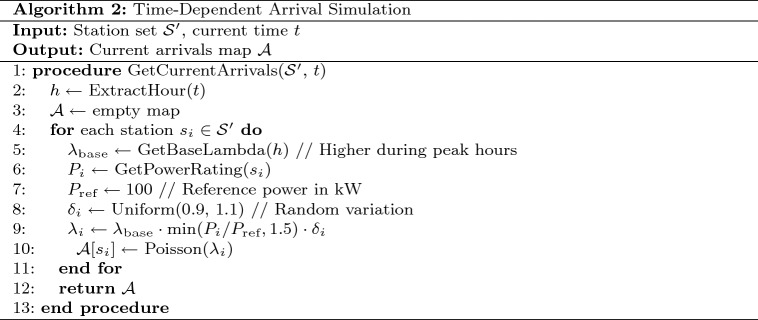
Time-dependent arrival simulation algorithm.

**Table 4:**
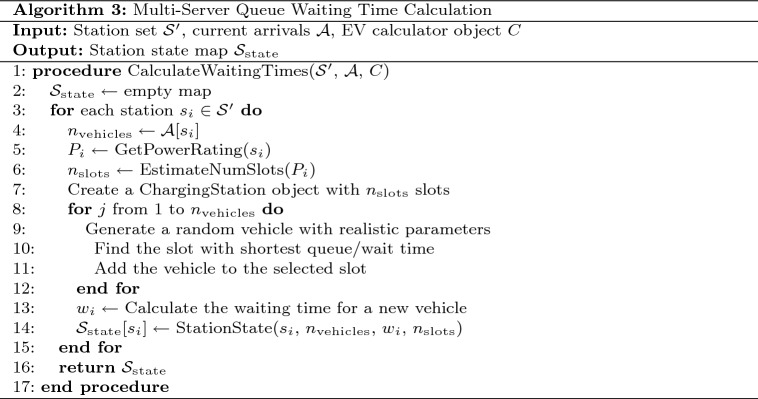
Waiting time calculation algorithm.

**Fig. 10:**
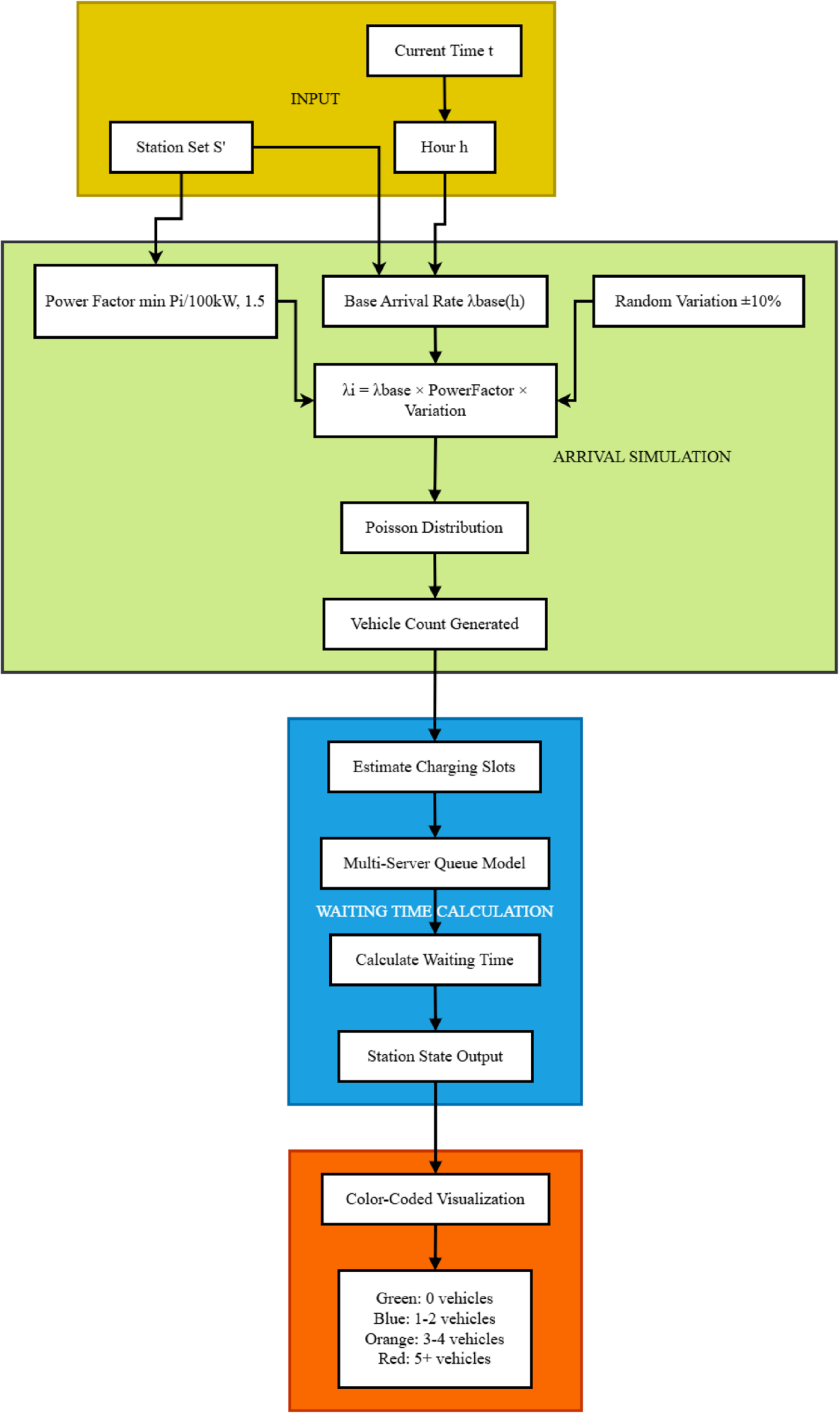
Time-dependent arrival simulation and waiting time calculation framework.

The time-dependent arrival simulation framework models charging station occupancy dynamics, as shown in Fig. 10. Starting with current time and station data, it adjusts arrival rates using temporal patterns and station attributes. A Poisson-based model with random variation simulates realistic vehicle arrivals, while a multi-server queue system estimates waiting times and manages charging slot availability. This enables accurate, real-time predictions of station congestion, allowing the routing system to minimize journey time through smart queue avoidance and optimized charging stop selection.

**Fig. 11:**
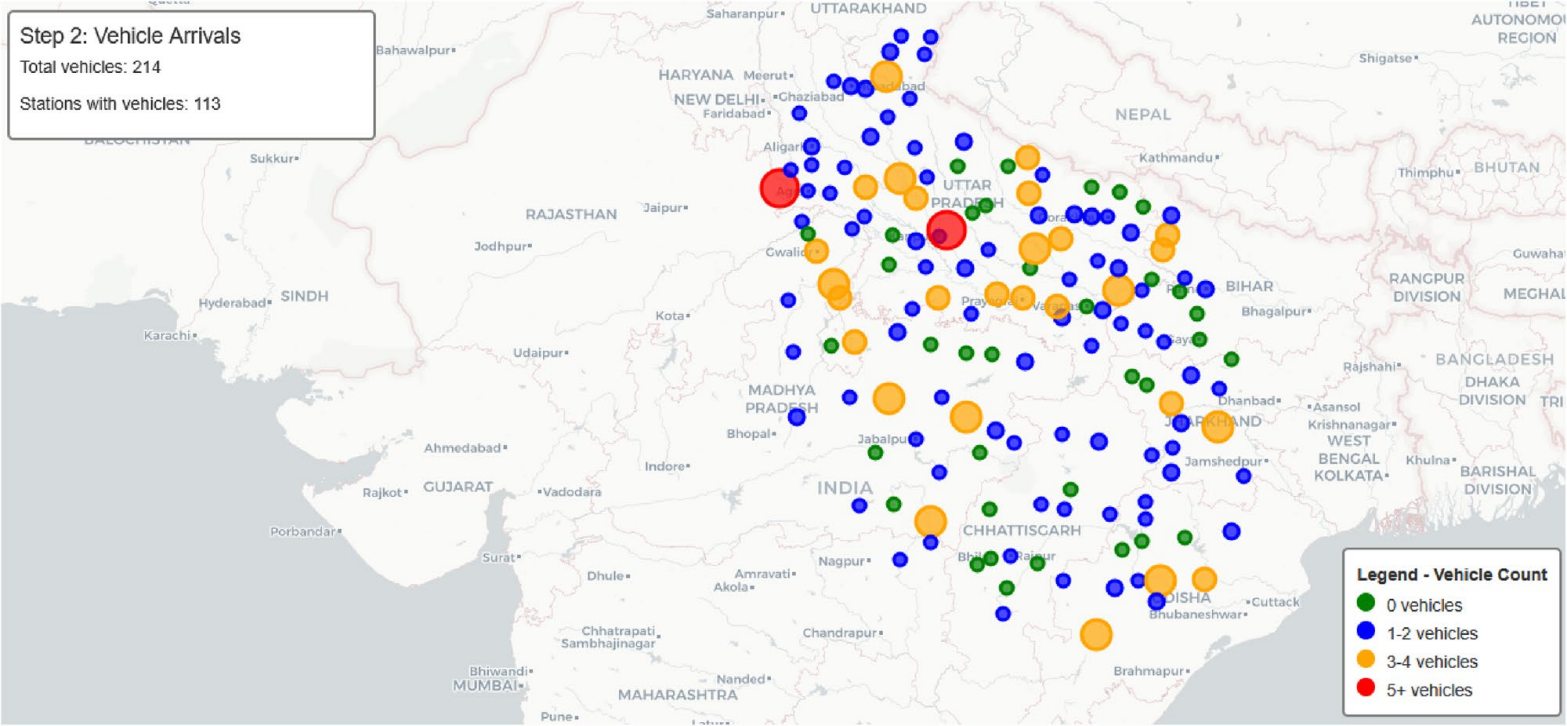
Time-dependent vehicle arrival distribution at charging stations.map generated using openrouteservice v9.0.0 (https://openrouteservice.org/).

**Fig. 12:**
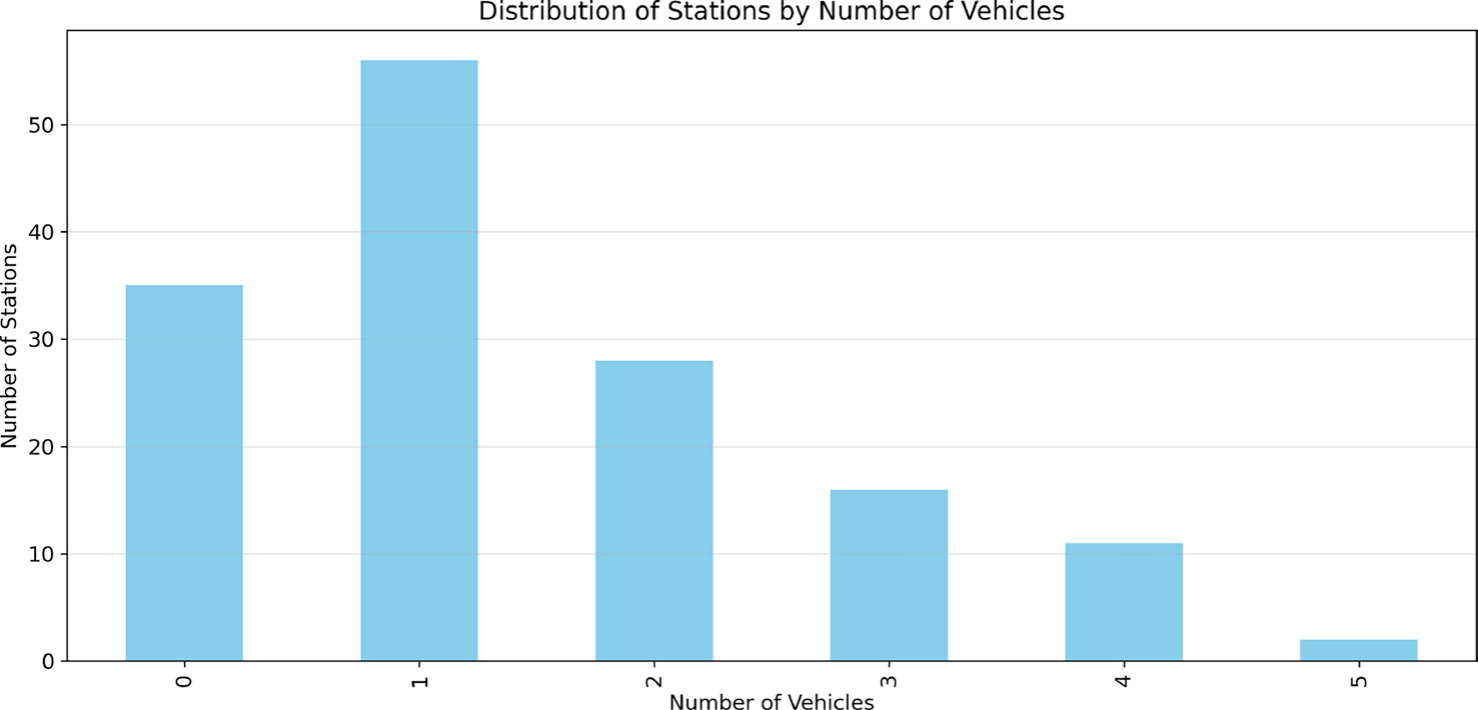
Distribution of stations by number of vehicles, showing the frequency of different occupancy levels.

This approach provides realistic estimations of expected waiting times at different stations, which is crucial for overall journey time optimization. Fig. 11 visualizes the vehicle arrival distribution across stations, with color-coding indicating the number of vehicles at each station (green for 0 vehicles, blue for 1–2 vehicles, orange for 3–4 vehicles, and red for 5+ vehicles).

The statistical distribution of vehicles across stations is analyzed in Fig. 12, providing insights into the overall system load and identifying potentially congested areas. Our implementation uses the Poisson distribution to model the stochastic nature of vehicle arrivals, ensuring realistic simulation of charging station dynamics.

**Fig. 13:**
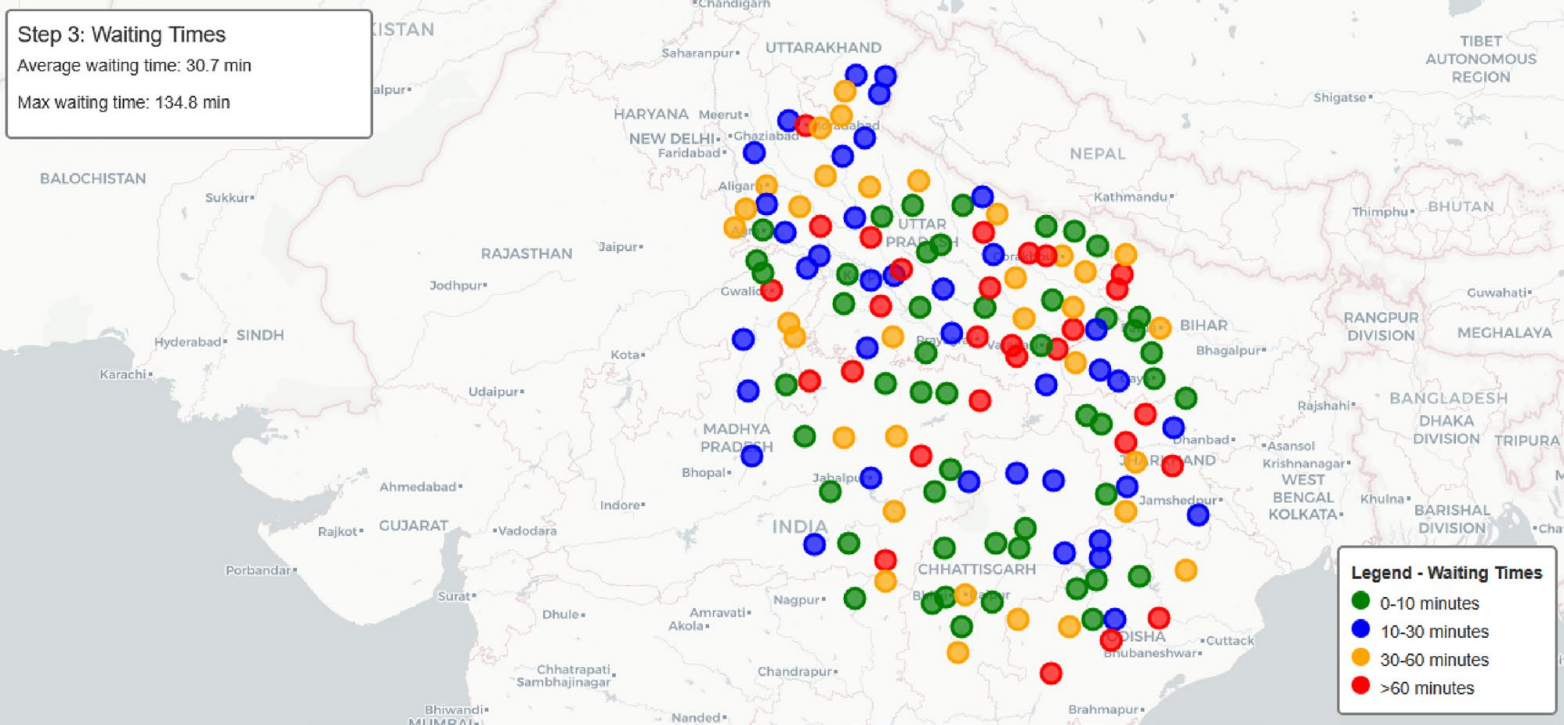
Phase-II: waiting time distribution across charging stations.map generated using openrouteservice v9.0.0 (https://openrouteservice.org/).

Building upon the arrival simulation, Fig. 13 presents a geographical visualization of waiting times across the network, with colors indicating different waiting time ranges: green for 0–10 minutes, blue for 10–30 minutes, orange for 30–60 minutes, and red for over 60 minutes.

**Fig. 14:**
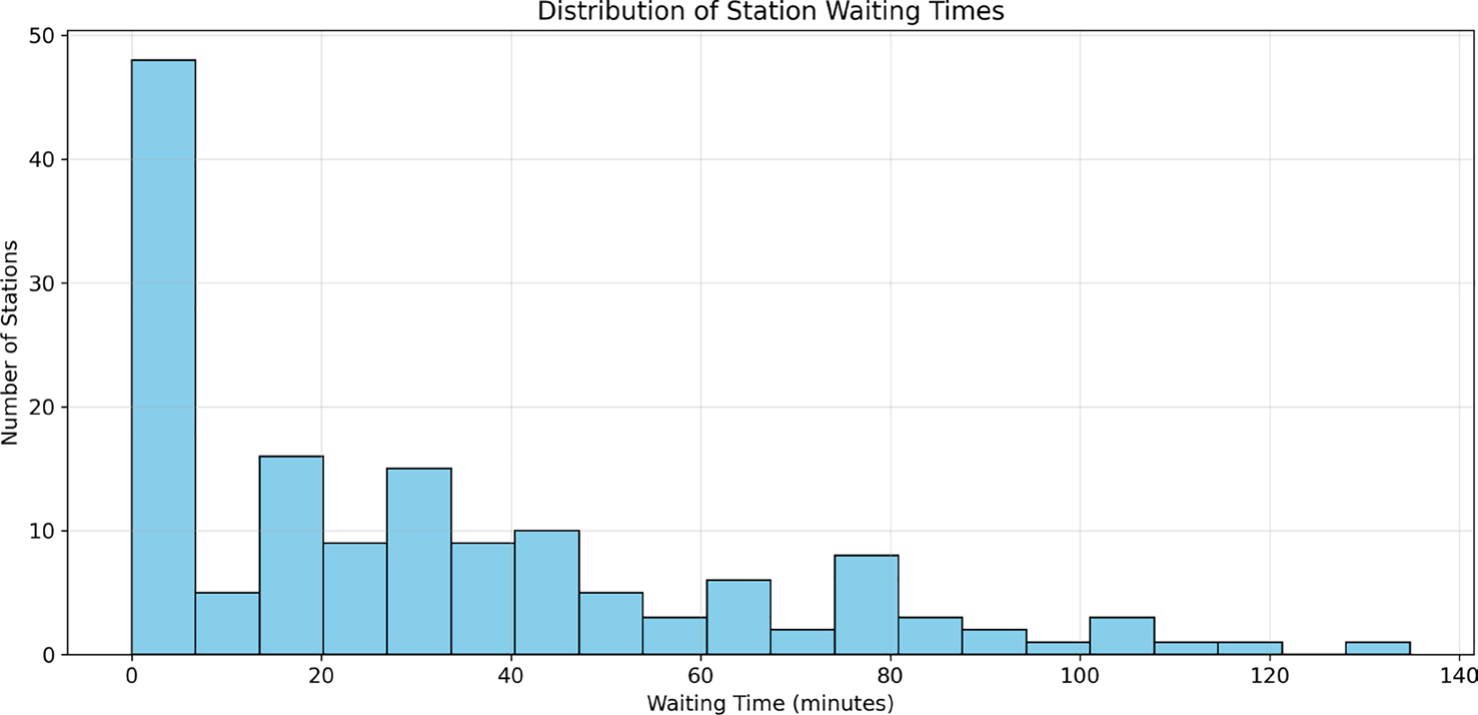
Distribution of station waiting times, showing the frequency of different waiting durations.

**Fig. 15:**
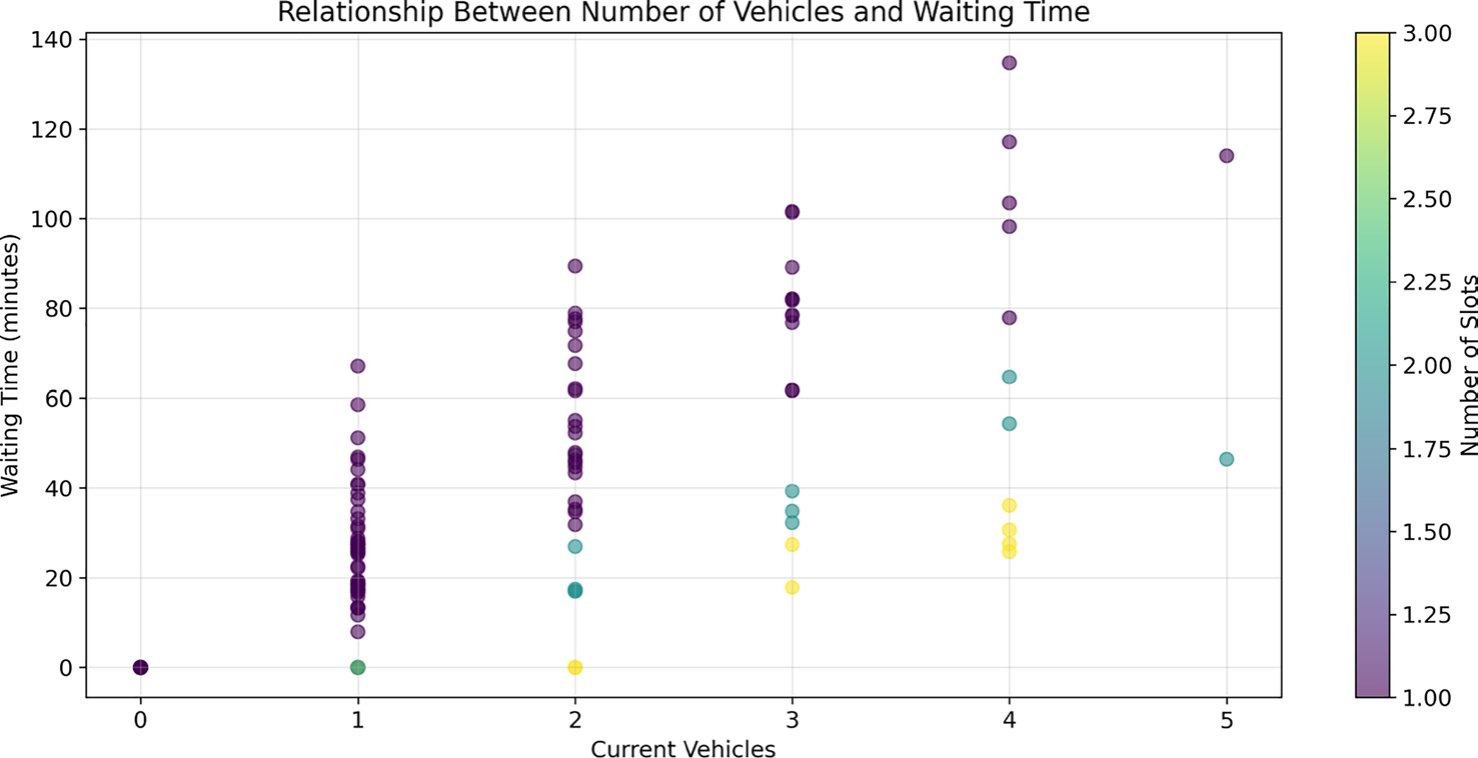
Relationship between number of vehicles and waiting time, with color indicating the number of charging slots.

The statistical distribution of waiting times is shown in Fig. 14, and the relationship between the number of vehicles and waiting times is analyzed in Fig. 15, revealing how station capacity influences wait durations. Our analysis indicates that stations with more than 3 vehicles typically exhibit waiting times exceeding 30 minutes, making them less favorable candidates for routing decisions.

#### Phase III - fuzzy reinforcement learning for weight calculation

Phase III introduces a core innovation of the system: the application of fuzzy reinforcement learning (FRL) to dynamically determine optimal charging station weights. Traditional routing algorithms typically use fixed weightings for different factors, which cannot adapt to the complex interplay between spatial and temporal variables. Our FRL approach combines the interpretability of fuzzy logic with the adaptive capabilities of reinforcement learning. Table 5 presents the pseudocode for our novel fuzzy RL weight calculation algorithm.

**Table 5:**
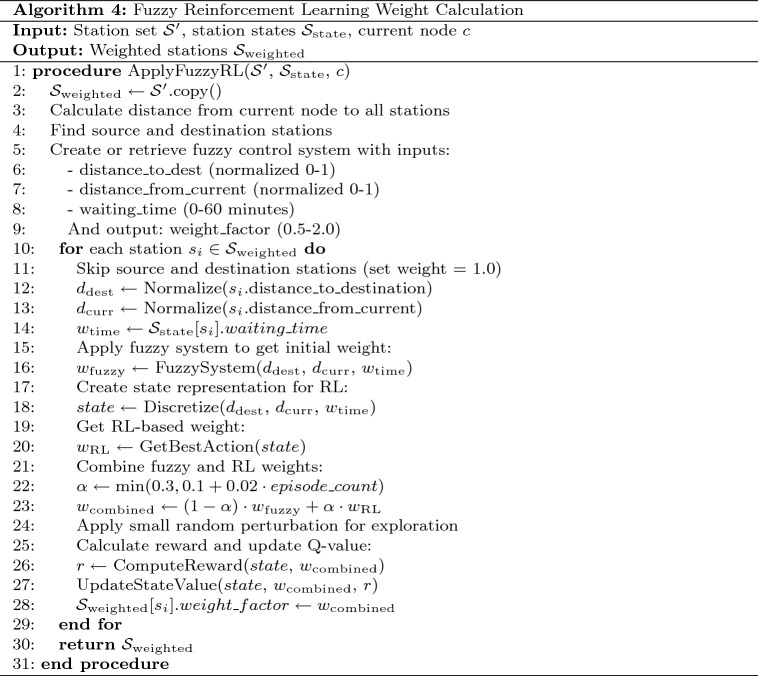
Fuzzy reinforcement learning weight calculation algorithm.

**Fig. 16:**
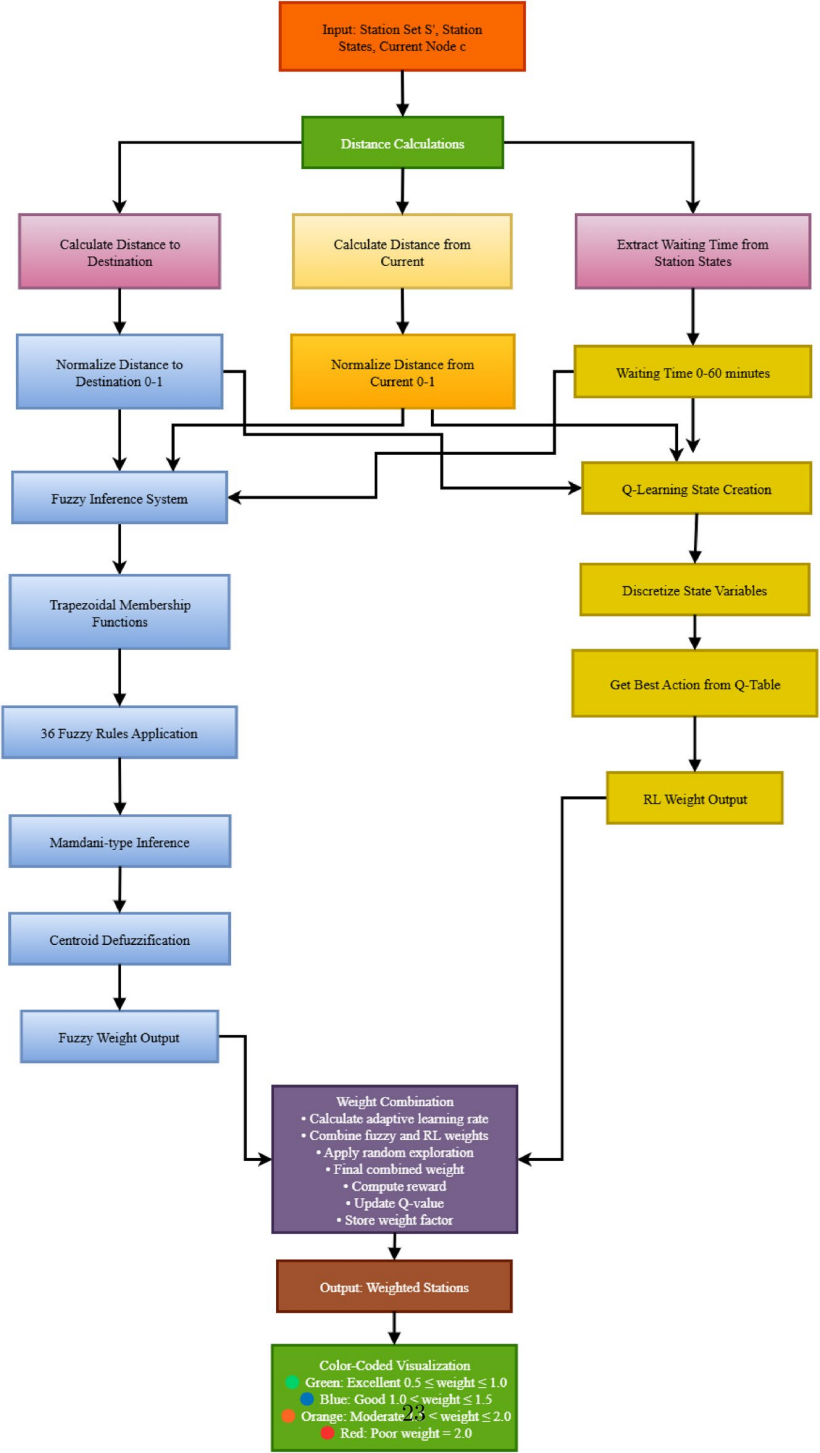
Fuzzy reinforcement learning weight calculation process with integrated decision-making framework.

The Fuzzy Reinforcement Learning component represents a core innovation of our system, implementing an adaptive weight calculation process as shown in Fig. 16. The process begins with distance calculations and waiting time extraction, followed by normalization of input variables for fuzzy inference processing. The fuzzy inference system employs trapezoidal membership functions and 36 optimization rules to generate initial weight factors, while the Q-learning component creates discretized state representations for reinforcement learning adaptation. The integration of fuzzy logic interpretability with reinforcement learning adaptability enables dynamic station weighting that evolves based on real-world performance feedback. The final weight combination process incorporates adaptive learning rates and exploration mechanisms, resulting in color-coded visualizations that clearly indicate station desirability levels from excellent (green) to poor (red) based on multi-dimensional optimization criteria.

The fuzzy component employs a Mamdani-type fuzzy inference system with three input variables:

1. Normalized distance to destination
2. Normalized distance from current location
3. Waiting time at the station

Each input variable is fuzzified using trapezoidal membership functions into linguistic categories. The fuzzy rule base consists of 36 rules that map input combinations to appropriate weight factor outputs, which are defuzzified using the centroid method.

***Detailed Fuzzy System Specification and Weight Determination Framework:***

The fuzzy inference system implements a Mamdani-type inference mechanism that converts continuous numerical inputs into discrete linguistic categories through trapezoidal membership functions, subsequently applying a comprehensive rule base to determine optimal weight factors. This approach, introduced by Mamdani and Assilian^45^, enables non-linear decision-making while maintaining mathematical interpretability and computational efficiency. This approach enables non-linear decision-making while maintaining mathematical interpretability and computational efficiency.

The weight determination process operates through systematic mapping of input variable combinations to appropriate weight outputs, where each variable contributes according to its contextual significance in the specific routing scenario. The system processes three normalized input variables: distance to destination (range [0,1]), distance from current location (range [0,1]), and waiting time (range [0,60] minutes), generating weight factors in the range [0.5,2.0] through defuzzification using the centroid method.

Table 6 presents the comprehensive mathematical specifications of all input and output variables used in the fuzzy inference system. Each input variable employs trapezoidal membership functions with overlapping regions to ensure smooth transitions between linguistic categories and prevent discontinuous weight assignments at category boundaries.

**Table 6:** Fuzzy input and output variable definitions.

**Variable**	**Category**	**Range**	**Description**
**Distance to Destination (Normalized 0–1)**	Very Close	0.0–0.35	Stations within 35% of maximum distance to destination
	Close	0.15–0.6	Stations within 60% of maximum distance to destination
	Medium	0.4–0.85	Stations at moderate distance to destination
	Far	0.65–1.0	Stations at substantial distance to destination
**Distance from Current (Normalized 0–1)**	Very Close	0.0–0.35	Stations within 35% of maximum distance from current position
	Close	0.15–0.6	Stations within 60% of maximum distance from current position
	Medium	0.4–0.85	Stations at moderate distance from current position
	Far	0.65–1.0	Stations at substantial distance from current position
**Waiting Time (Minutes)**	Short	0–20 min	Minimal queue, immediate availability
	Medium	10–40 min	Moderate queue, acceptable wait
	Long	30–60 min	Significant queue, substantial delay
**Weight Factor (Output)**	Very Low	0.5–0.9	Highly preferred stations
	Low	0.7–1.1	Preferred stations
	Medium	0.9–1.3	Acceptable stations
	High	1.1–1.7	Less preferred stations
	Very High	1.5–2.0	Least preferred stations

Each trapezoidal membership function in Table 6 is mathematically defined using four parameters [a, b, c, d] according to:

4 $$\begin{aligned} \mu (x) = \max \left( \min \left( \frac{x-a}{b-a}, 1, \frac{d-x}{d-c}\right) , 0\right) \end{aligned}$$

Trapezoidal membership functions provide plateau regions of full membership, better representing real-world scenarios where values within a range are equally representative of a linguistic category^46^. Table 7 provides the precise mathematical parameters for complete reproducibility:

**Table 7:** Trapezoidal membership function mathematical parameters.

**Variable**	**Category**	**Parameters [a,b,c,d]**	**Mathematical Function**
Distance to Destination	Very Close	[0.0, 0.0, 0.2, 0.35]	$\mu _{vc}(x) = \max (\min (1, (0.35-x)/0.15), 0)$
	Close	[0.15, 0.3, 0.45, 0.6]	$\mu _c(x) = \max (\min ((x-0.15)/0.15, 1, (0.6-x)/0.15), 0)$
	Medium	[0.4, 0.55, 0.7, 0.85]	$\mu _m(x) = \max (\min ((x-0.4)/0.15, 1, (0.85-x)/0.15), 0)$
	Far	[0.65, 0.8, 1.0, 1.0]	$\mu _f(x) = \max (\min ((x-0.65)/0.15, 1), 0)$
Distance from Current	Very Close	[0.0, 0.0, 0.2, 0.35]	$\mu _{vc}(x) = \max (\min (1, (0.35-x)/0.15), 0)$
	Close	[0.15, 0.3, 0.45, 0.6]	$\mu _c(x) = \max (\min ((x-0.15)/0.15, 1, (0.6-x)/0.15), 0)$
	Medium	[0.4, 0.55, 0.7, 0.85]	$\mu _m(x) = \max (\min ((x-0.4)/0.15, 1, (0.85-x)/0.15), 0)$
	Far	[0.65, 0.8, 1.0, 1.0]	$\mu _f(x) = \max (\min ((x-0.65)/0.15, 1), 0)$
Waiting Time	Short	[0, 0, 10, 20]	$\mu _s(x) = \max (\min (1, (20-x)/10), 0)$
	Medium	[10, 20, 30, 40]	$\mu _m(x) = \max (\min ((x-10)/10, 1, (40-x)/10), 0)$
	Long	[30, 40, 60, 60]	$\mu _l(x) = \max (\min ((x-30)/10, 1), 0)$
Weight Factor	Very Low	[0.5, 0.5, 0.7, 0.9]	$\mu _{vl}(x) = \max (\min (1, (0.9-x)/0.2), 0)$
	Low	[0.7, 0.8, 1.0, 1.1]	$\mu _l(x) = \max (\min ((x-0.7)/0.1, 1, (1.1-x)/0.1), 0)$
	Medium	[0.9, 1.0, 1.2, 1.3]	$\mu _m(x) = \max (\min ((x-0.9)/0.1, 1, (1.3-x)/0.1), 0)$
	High	[1.1, 1.3, 1.5, 1.7]	$\mu _h(x) = \max (\min ((x-1.1)/0.2, 1, (1.7-x)/0.2), 0)$
	Very High	[1.5, 1.7, 2.0, 2.0]	$\mu _{vh}(x) = \max (\min ((x-1.5)/0.2, 1), 0)$

The complete fuzzy rule base is systematically presented in Table 8, which maps all possible combinations of input conditions to appropriate weight factor outputs. Each rule incorporates explicit decision logic that demonstrates the reasoning behind weight assignments, ensuring comprehensive coverage of routing scenarios while maintaining consistent decision-making principles.

**Table 8:** Complete 36-rule fuzzy decision matrix with decision logic.

**Rule**	**Distance from current**	**Distance to destination**	**Waiting Time**	**Weight factor**	**Decision logic**
1	very close	any	short	very low	Nearby, no wait - optimal choice
2	very close	any	medium	low	Nearby but moderate wait
3	very close	any	long	medium	Nearby but excessive wait
4	close	any	short	low	Reasonably close, no wait
5	close	any	medium	medium	Reasonably close, moderate wait
6	close	any	long	high	Close but excessive wait penalty
7	medium	any	short	medium	Moderate distance, no wait
8	medium	any	medium	high	Moderate distance with wait
9	medium	any	long	very high	Moderate distance, long wait
10	far	any	short	high	Far but no wait
11	far	any	medium	very high	Far with moderate wait
12	far	any	long	very high	Far with long wait - avoid
13	any	very close	short	very low	Near destination, no wait
14	any	very close	medium	low	Near destination, moderate wait
15	any	very close	long	medium	Near destination, long wait
16	any	close	short	low	Close to destination, no wait
17	any	close	medium	medium	Close to destination, moderate wait
18	any	close	long	high	Close to destination, long wait
19	any	medium	short	medium	Medium distance to destination
20	any	medium	medium	high	Medium distance, moderate wait
21	any	medium	long	very high	Medium distance, long wait
22	any	far	short	high	Far from destination
23	any	far	medium	very high	Far from destination with wait
24	any	far	long	very high	Far from destination, long wait
25	very close	very close	short	very low	Optimal: close to both, no wait
26	very close	close	short	very low	Excellent: close to both
27	close	very close	short	low	Good: prioritize destination
28	close	close	short	low	Good: close to both
29	close	close	medium	medium	Acceptable with moderate wait
30	medium	medium	medium	medium	Average option
31	medium	close	long	high	Close to destination but long wait
32	far	medium	long	very high	Poor: far from current with wait
33	far	far	short	high	Poor: far from both locations
34	far	far	medium	very high	Very poor: far with moderate wait
35	far	far	long	very high	Worst case: far from both, long wait
36	medium	far	long	very high	Poor: far from destination with wait

**Mathematical Processing Framework for 36-Rule Evaluation:**

The fuzzy inference process for the rules presented in Table 8 operates through four mathematical stages:

1. **Fuzzification:** Convert crisp inputs to membership degrees using the trapezoidal functions in Table 7.
2. **Rule Evaluation:** Calculate each rule’s firing strength by taking the minimum membership degree across all input variables:
  5 $$\begin{aligned} \alpha _i = \min (\mu _A(x_1), \mu _B(x_2), \mu _C(x_3)) \end{aligned}$$
  where $\alpha _i$ is the firing strength of rule *i*, and $\mu _A$, $\mu _B$, $\mu _C$ are the membership functions for distance from current, distance to destination, and waiting time respectively.The minimum operator implements the logical AND operation in Mamdani inference systems^45^, ensuring all antecedent conditions must be simultaneously satisfied.
3. **Aggregation:** Combine output membership functions using the maximum operator:
  6 $$\begin{aligned} \mu _{output}(w) = \max _i(\alpha _i \cdot \mu _{output_i}(w)) \end{aligned}$$
  where $\mu _{output}(w)$ represents the aggregated membership degree for weight value *w*.The maximum operator implements the logical OR across rules in the Mamdani framework^45^, allowing multiple rules to contribute to the final output.
4. **Defuzzification:** Calculate the final crisp weight value using the centroid method:
  7 $$\begin{aligned} w_{final} = \frac{\sum _j \mu _{output}(w_j) \cdot w_j}{\sum _j \mu _{output}(w_j)} \end{aligned}$$
  where $w_{final}$ is the crisp output weight factor in the range [0.5, 2.0].The centroid method computes the center of gravity of the aggregated output membership function^46^, providing smooth, stable outputs that minimize discontinuities in control applications. This makes it particularly suitable for real-time routing decisions where weight factors must change gradually as vehicle position and station conditions evolve.

**Reinforcement Learning Component Integration:**

The fuzzy inference system provides domain-knowledge-driven initial weight assignments, which are subsequently refined through temporal difference learning to achieve adaptive station preference optimization:

1. **State Space Formulation:** The routing decision state $s_t$ is defined as:
  8 $$\begin{aligned} s_t = \{SOC_t, d_{remaining}, \mathcal {C}_t, \textbf{W}_t\} \end{aligned}$$
  where $SOC_t$ represents normalized state-of-charge, $d_{remaining}$ denotes distance to destination, $\mathcal {C}_t$ is the feasible charging station set, and $\textbf{W}_t$ represents the real-time waiting time vector.
2. **Multi-Objective Reward Structuring:** The composite reward function integrates temporal efficiency, spatial optimization, and service quality metrics:
  9 $$\begin{aligned} R(s_t, a_t)&= \omega _1 r_{temporal}(a_t) + \omega _2 r_{spatial}(a_t) + \omega _3 r_{success}(a_t) \end{aligned}$$
  10 $$\begin{aligned} r_{temporal}(a_t)&= -\alpha \cdot W_{actual}(a_t) - \beta \cdot T_{service}(a_t) \end{aligned}$$
  11 $$\begin{aligned} r_{spatial}(a_t)&= -\gamma \cdot D_{detour}(a_t) \end{aligned}$$
  12 $$\begin{aligned} r_{success}(a_t)&= \delta \cdot \mathbb {I}_{completion}(a_t) \end{aligned}$$
  where $\mathbb {I}_{completion}(a_t)$ is the indicator function for successful charging completion.
3. **Q-Learning Implementation:** The system updates station preferences using:
  13 $$\begin{aligned} Q(s_t, a_t) \leftarrow Q(s_t, a_t) + \alpha \left[ R(s_t, a_t) + \gamma \max _{a'} Q(s_{t+1}, a') - Q(s_t, a_t) \right] \end{aligned}$$
  with learning rate $\alpha$ and discount factor $\gamma$ controlling temporal credit assignment.
4. **Hierarchical Weight Synthesis:** The integrated weight factors combine fuzzy domain knowledge with learned empirical performance:
  14 $$\begin{aligned} w_{integrated}(s_i) = w_{fuzzy}(s_i) \cdot \exp \left( \frac{Q(s_t, s_i) - Q_{min}}{Q_{max} - Q_{min}}\right) \end{aligned}$$
  where $w_{fuzzy}(s_i)$ represents the baseline fuzzy weight from Equation (4).

**Variable Weight Allocation in Fuzzy Rule Structure:**

The fuzzy system implements a hierarchical decision framework where each variable contributes based on contextual significance:

- **Distance to Destination:** Addressed in rules 13–24 and 25–36 (Table 8), focusing on trip completion efficiency and destination proximity optimization.
- **Waiting Time:** Incorporated across all 36 rules as a temporal constraint factor, accounting for queue dynamics and real-time availability patterns.
- **Distance from Current Location:** Utilized in rules 1–12 and 25–36 for immediate routing efficiency and detour minimization.

This approach eliminates predetermined weight coefficients by embedding variable importance directly within the rule-based inference mechanism, enabling adaptive weight determination that responds to specific routing scenario characteristics.

**Fig. 17:**
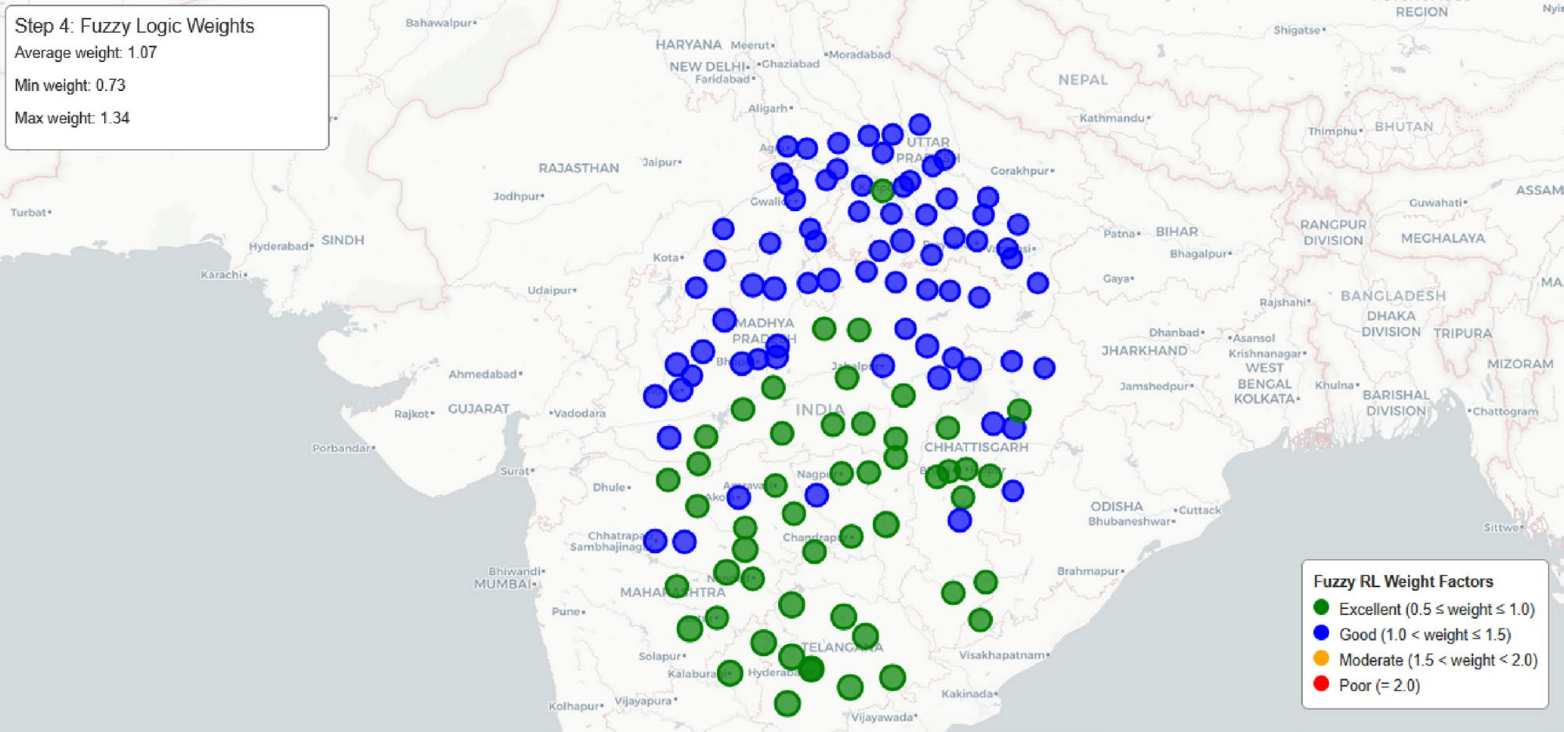
Phase-III: fuzzy RL weight factor distribution across charging stations.map generated using openrouteservice v9.0.0 (https://openrouteservice.org/).

**Fuzzy System Output Visualization and Validation:**

Figure 17 demonstrates the practical application of the fuzzy inference system by showing final outputs across the charging network:

- **Green Dots (Excellent:** $0.5 \le \textrm{weight} \le 1.0$): Output of fuzzy rules assigning “Very Low” and “Low” weight factors, primarily rules 1, 4, 13, 16, 25, 26, 27, 28 for stations close to current/destination with short waiting times.
- **Blue Dots (Good:** $1.0 < \textrm{weight} \le 1.5$): Result from rules producing “Low” to “Medium” weight factors, typically rules 2, 5, 14, 17, 29 where stations have moderate distances or waiting times.
- **Orange Dots (Moderate:** $1.5< \textrm{weight} \ <2.0$): Emerge from rules generating “Medium” to “High” weight factors, such as rules 3, 6, 8, 15, 18, 20, 31 where distances are substantial or waiting times elevated.
- **Red Dots (Poor:** $\textrm{weight} = 2.0$): Result from rules producing “High” to “Very High” weight factors, primarily rules 9, 11, 12, 21, 23, 24, 32, 33, 34, 35, 36 where multiple unfavorable conditions combine.

Figure 17 validates the transparency and credibility of the fuzzy inference system by demonstrating: (1) smooth geographical distribution showing effective trapezoidal membership functions from Table 7, (2) spatial clustering patterns confirming accurate 36-rule classification from Table 8, (3) heterogeneous but logical distribution proving meaningful centroid defuzzification outputs, and (4) presence of excellent stations across various regions demonstrating real-time adaptability rather than fixed spatial biases.

**Fig. 18:**
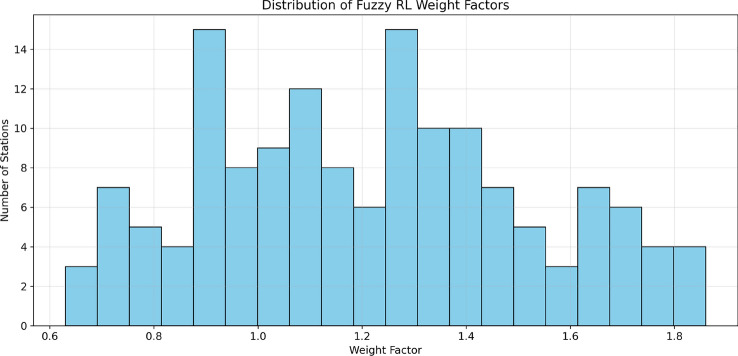
Distribution of fuzzy RL weight factors, showing the frequency of different weight values.

The implementation utilizes Scikit-fuzzy for the fuzzy inference system and a custom reinforcement learning framework. The statistical distribution of weight factors is presented in Fig. 18, showing frequency of different weight values across the station network with values ranging from 0.63 to 1.86 and an average of approximately 1.21.

**Fig. 19:**
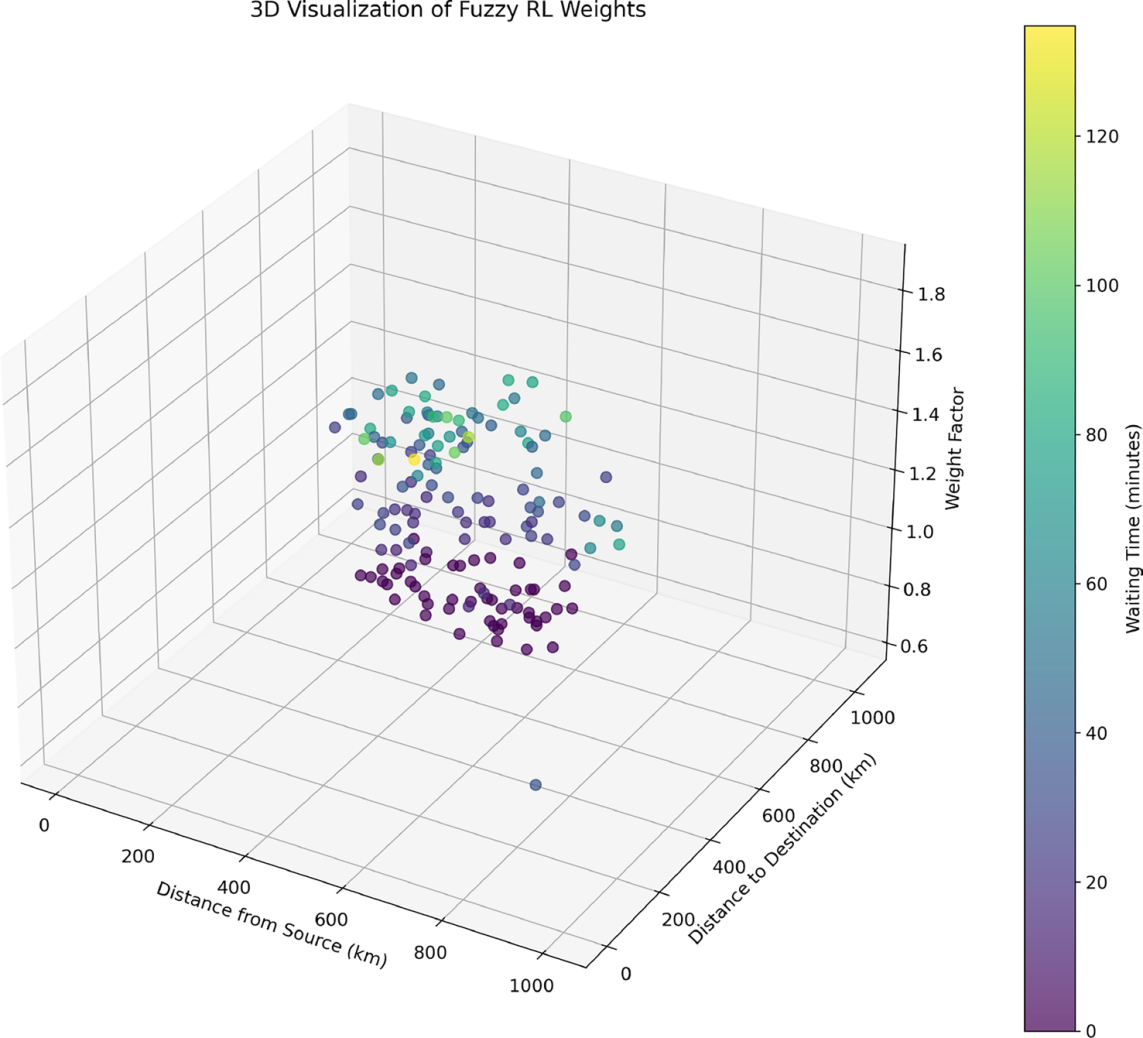
3D visualization of fuzzy RL weights. created using Matplotlib 3.10.1 in Python 3.13.2 with NumPy 2.1.3 (https://www.python.org/).

Figure 19 provides a three-dimensional visualization of the integrated fuzzy-RL system by plotting actual weight factors against contributing variables: X-axis represents distance from current EV location, Y-axis shows distance to destination, Z-axis displays final integrated weight factors , and color represents real-time waiting time. This visualization confirms that lower weight factors cluster near origin (0,0) representing optimal proximity conditions, higher waiting times correlate with increased weight factors across all spatial positions, and RL adaptation creates weight variations beyond spatial factors alone, demonstrating the advantage of the fuzzy-RL approach over fixed weighting schemes while maintaining interpretability and achieving adaptive optimization capabilities.

#### Phase IV - enhanced A* algorithm with traffic and elevation

Phase IV serves as the core routing engine, implementing an enhanced version of the A* algorithm that incorporates multiple real-world constraints. Table 9 presents the pseudocode for our enhanced A* algorithm.

**Table 9:**
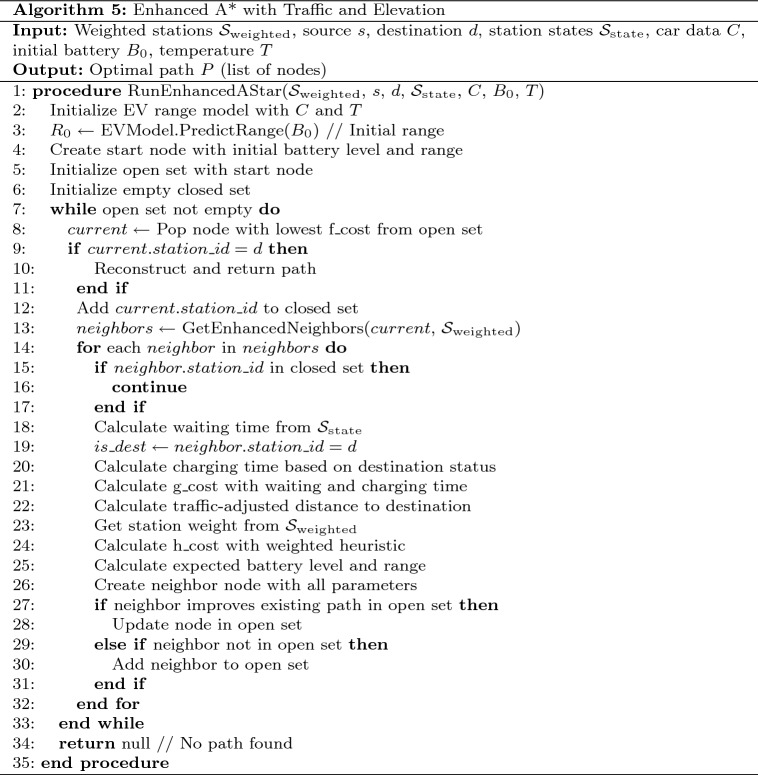
Enhanced A* algorithm with traffic and elevation.

**Fig. 20:**
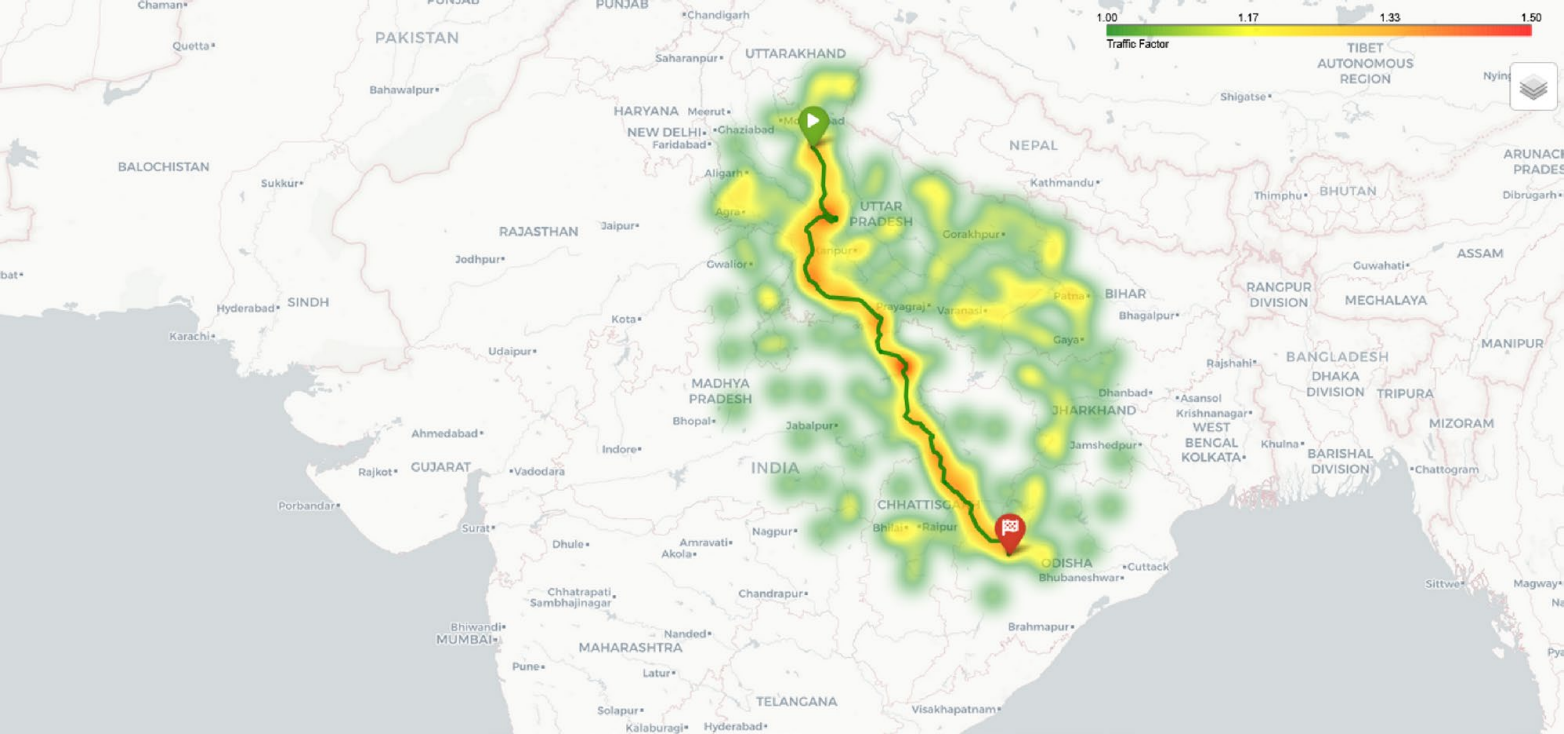
Traffic heatmap visualization showing traffic intensity across the route area.Map generated using openrouteservice v9.0.0 (https://openrouteservice.org/).

**Fig. 21:**
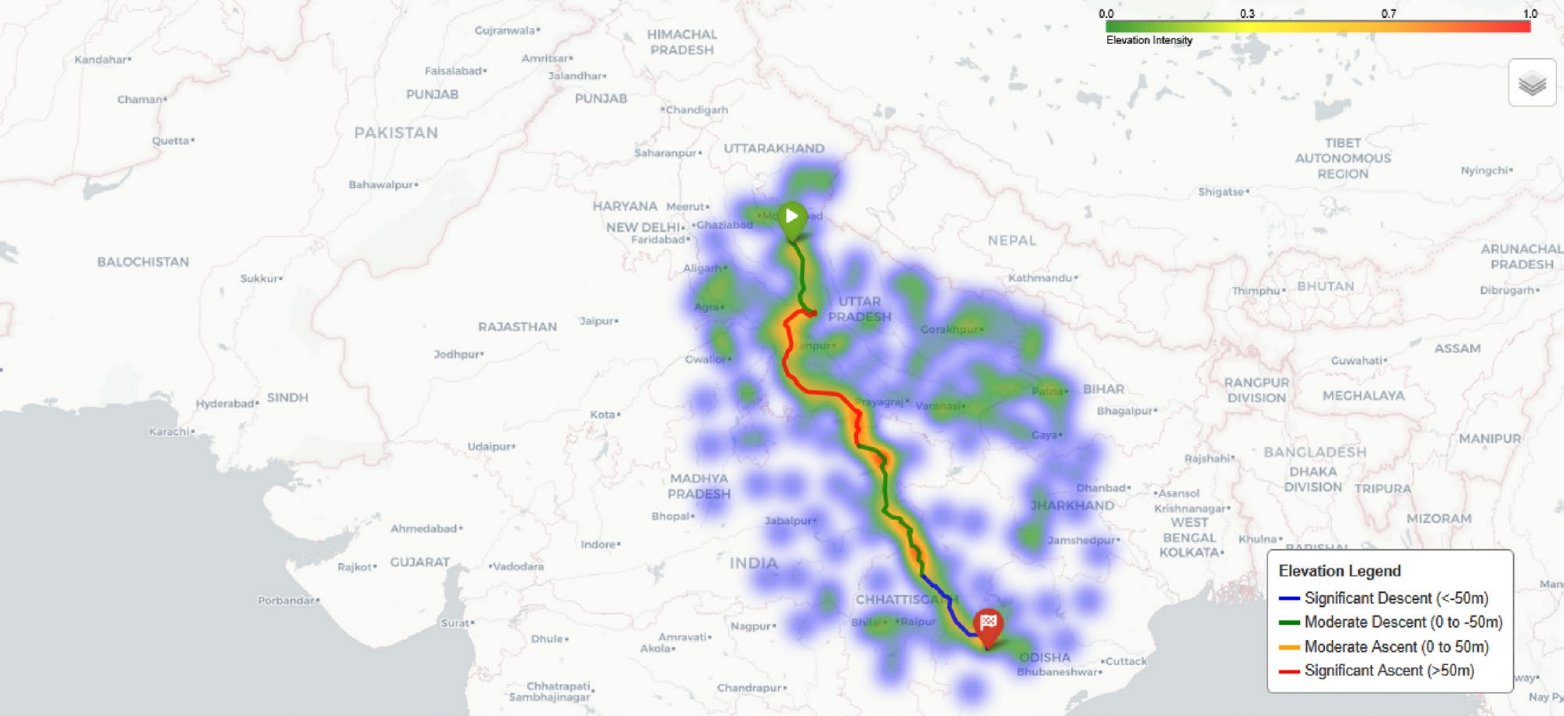
Elevation heatmap visualization showing elevation changes across the route area.Map generated using openrouteservice v9.0.0 (https://openrouteservice.org/).

Traditional A* implementations use a simple distance heuristic, but our approach extends the algorithm to consider:

1. **Battery Constraints:** Each node in the search space tracks the vehicle’s battery level, which decreases based on distance traveled and increases after charging stops.
2. **Traffic Conditions:** Real-time traffic data is integrated through a dedicated API adapter, providing traffic factors that modify both travel time estimations and energy consumption rates. Fig. 20 shows a traffic heatmap visualization that illustrates traffic conditions across the route area, implemented using Folium’s HeatMap functionality with custom color gradients.
3. **Elevation Profiles:** An elevation API adapter retrieves topographical data along route segments and calculates energy impact based on potential energy changes:
  15 $$\begin{aligned} E_{\text {ascent}}= & \frac{m \cdot g \cdot \Delta h_{\text {ascent}}}{3600000 \cdot \eta } \end{aligned}$$
  16 $$\begin{aligned} E_{\text {descent}}= & -\frac{m \cdot g \cdot \Delta h_{\text {descent}} \cdot \eta _{\text {regen}}}{3600000} \end{aligned}$$
  Where *m* is the vehicle mass, *g* is gravitational acceleration, $\Delta h$ represents elevation change, $\eta$ is drivetrain efficiency, and $\eta _{\text {regen}}$ is regenerative braking efficiency. Fig. 21 presents an elevation heatmap visualization that displays elevation changes along the route, implemented using custom gradient mapping for elevation data.
4. **Waiting and Charging Times:** The time spent at charging stations is explicitly included in the cost function, making it a true time-minimizing algorithm rather than simply a distance-minimizing one.

**Fig. 22:**
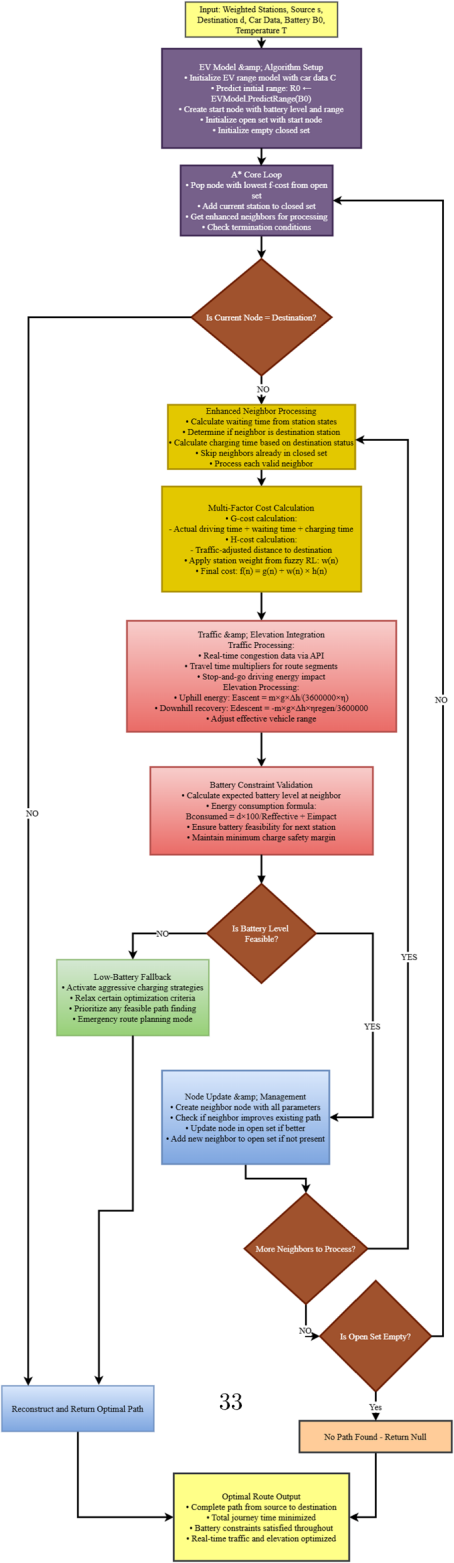
Enhanced A* algorithm flowchart with multi-factor optimization and battery constraint validation.

The Enhanced A* algorithm implementation follows a sophisticated decision-making flowchart that integrates multiple real-world constraints as shown in Fig. 22. The algorithm begins with EV model configuration and initial battery state assessment, then proceeds through iterative node expansion with comprehensive neighbor evaluation. Critical decision points include battery feasibility validation, traffic and elevation integration, and multi-factor cost calculations incorporating waiting times, charging durations, and energy consumption patterns. The flowchart demonstrates the algorithm’s ability to handle complex scenarios including emergency route planning when standard pathfinding fails due to extreme battery constraints. This systematic approach ensures optimal route selection while maintaining safety margins and realistic travel time estimations through continuous battery level monitoring and adaptive charging strategy implementation.

The modified A* algorithm uses a cost function $f(n) = g(n) + w(n) \cdot h(n)$, where:

- *g*(*n*) represents the actual time cost to reach node *n*, including driving, waiting, and charging time
- *h*(*n*) is the estimated time to reach the destination from node *n*
- *w*(*n*) is the adaptive weight factor derived from the fuzzy reinforcement learning component

Node expansion during the search process is constrained by battery feasibility, ensuring that the vehicle always maintains sufficient charge to reach the next station. For each potential edge in the route, our implementation calculates:

17 $$\begin{aligned} B_{\text {consumed}} = \frac{d \cdot 100}{R_{\text {effective}}} + E_{\text {impact}} \end{aligned}$$

Where *d* denotes edge distance (km), $R_{\text {effective}}$ is traffic-adjusted range, and $E_{\text {impact}}$ accounts for elevation-induced battery impact.

**Fig. 23:**
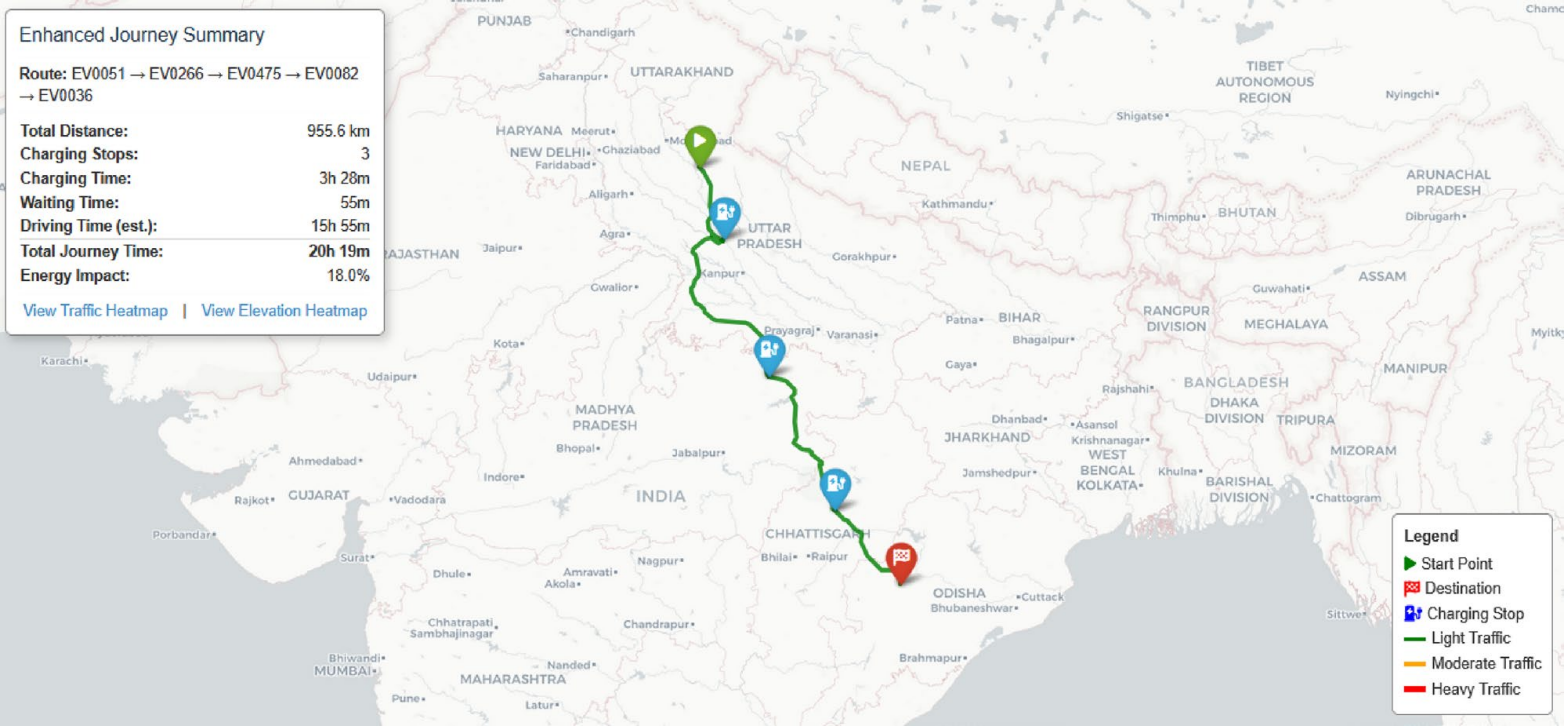
Phase-IV: Final optimized route with traffic and elevation data.map generated using openrouteservice v9.0.0 (https://openrouteservice.org/).

The enhanced A* algorithm computes an optimal path that minimizes journey time while respecting battery constraints. Fig. 23 illustrates the final route with traffic-based coloring (green: light, orange: moderate, red: heavy) and key journey metrics. A fallback mechanism is included to handle infeasible cases by activating a low-battery pathfinder that prioritizes feasibility using adaptive charging strategies and relaxed constraints.

### Journey analysis and metrics

The output of the system includes comprehensive journey analysis visualizations that provide insights into various aspects of the planned route, enabling users to make informed decisions about their electric vehicle travel while addressing critical concerns such as range anxiety, charging logistics, and journey optimization.

#### Battery level monitoring and safety analysis

**Fig. 24:**
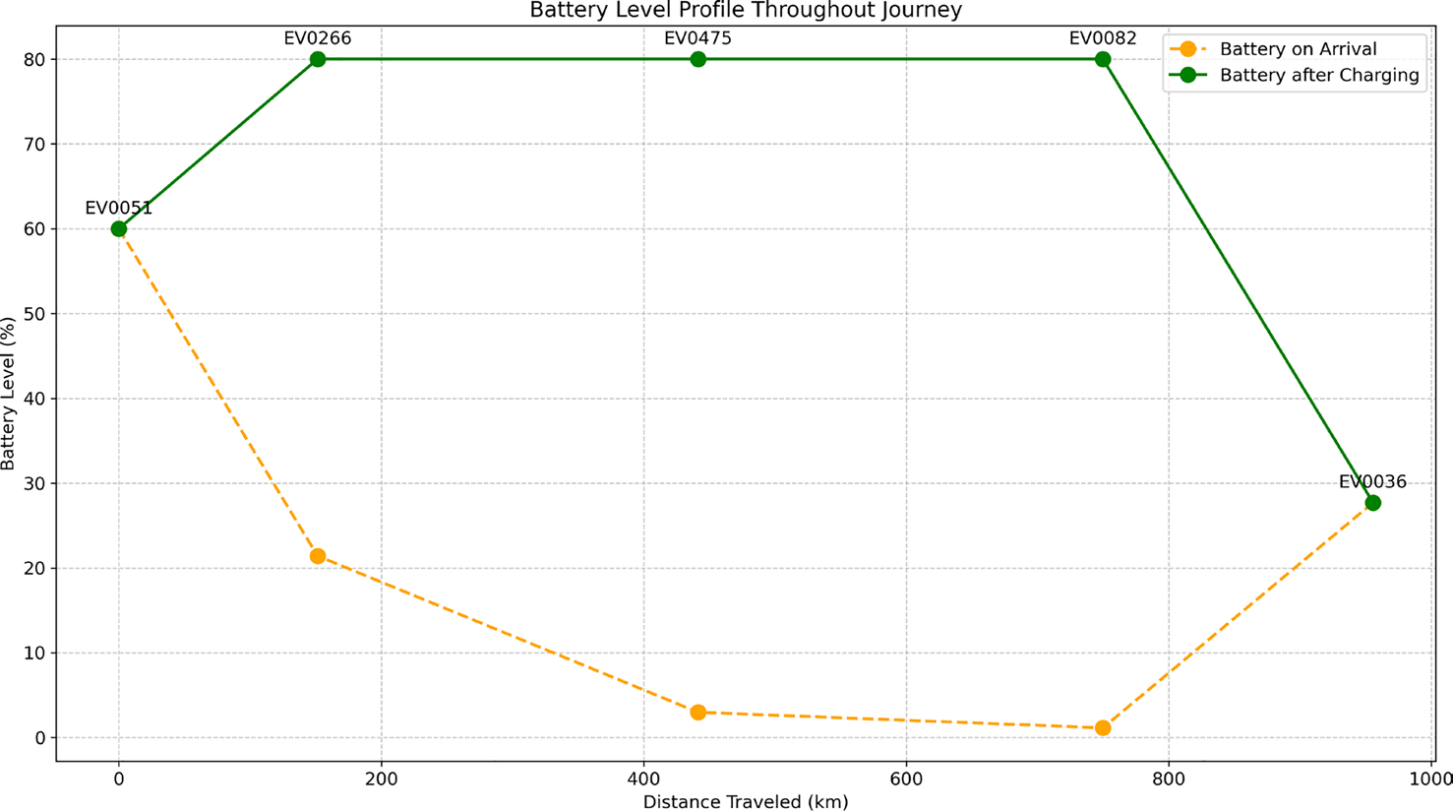
Battery level profile throughout the journey, showing levels at arrival and after charging at each station.

Figure 24 displays battery levels across the route, showing arrival (orange) and post-charging (green) levels at each station. This helps visualize energy use and charging effectiveness. The system ensures safety margins, maintaining at least 10% at intermediate stops and 5% at the destination. The profile reflects the influence of elevation, traffic, and driving patterns on battery consumption, aiding future planning.

#### Charging infrastructure utilization analysis

**Fig. 25:**
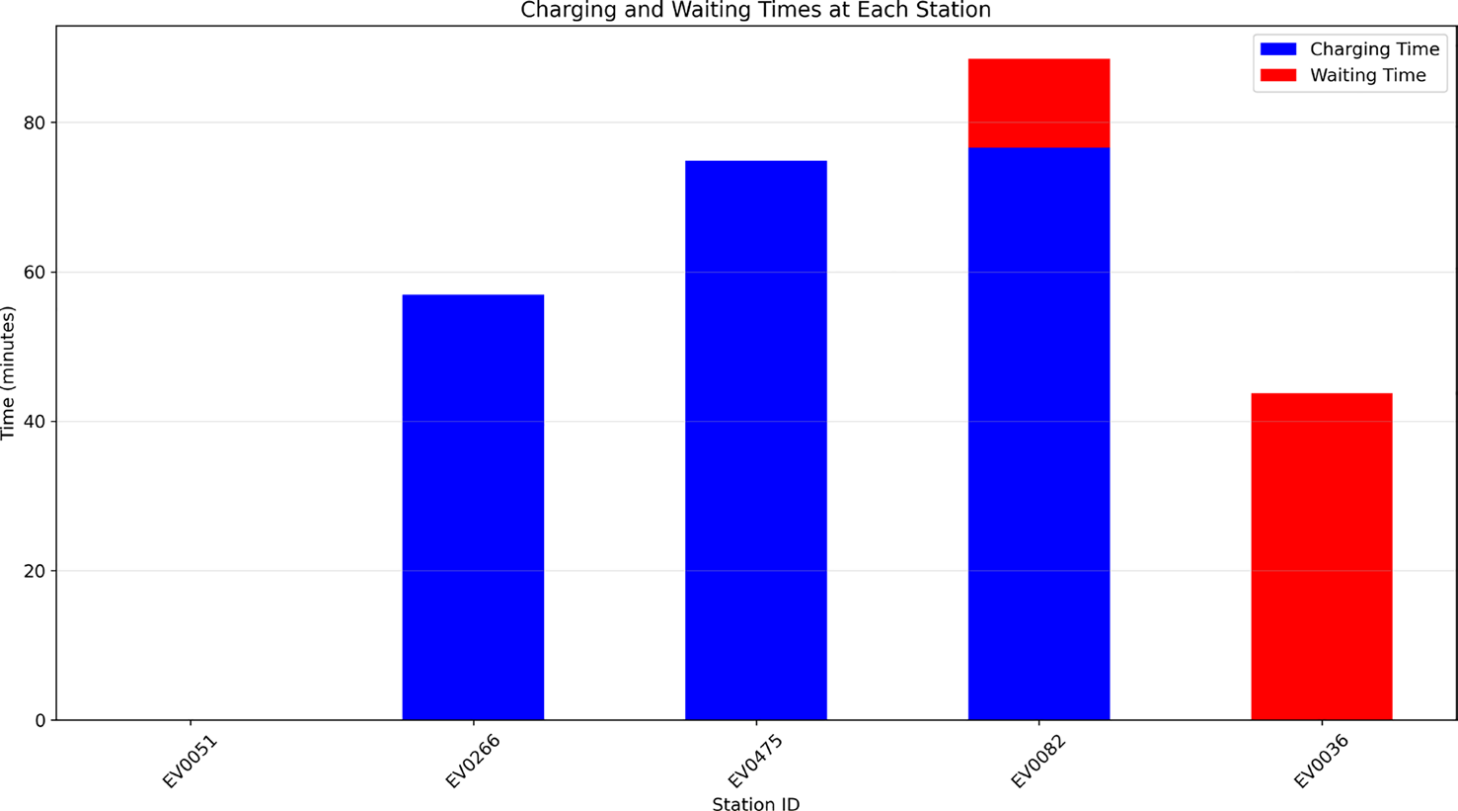
Charging and waiting times at each station, showing the breakdown of time spent at each stop.

Figure 25 illustrates charging and waiting times at each station, helping identify non-driving delays along the route. Fuzzy reinforcement learning minimizes waiting by selecting less congested stations. Charging efficiency is improved by considering station power, battery behavior, and required charge levels, with smart detours and timed stops further enhancing overall performance.

#### Real-time traffic impact assessment

**Fig. 26:**
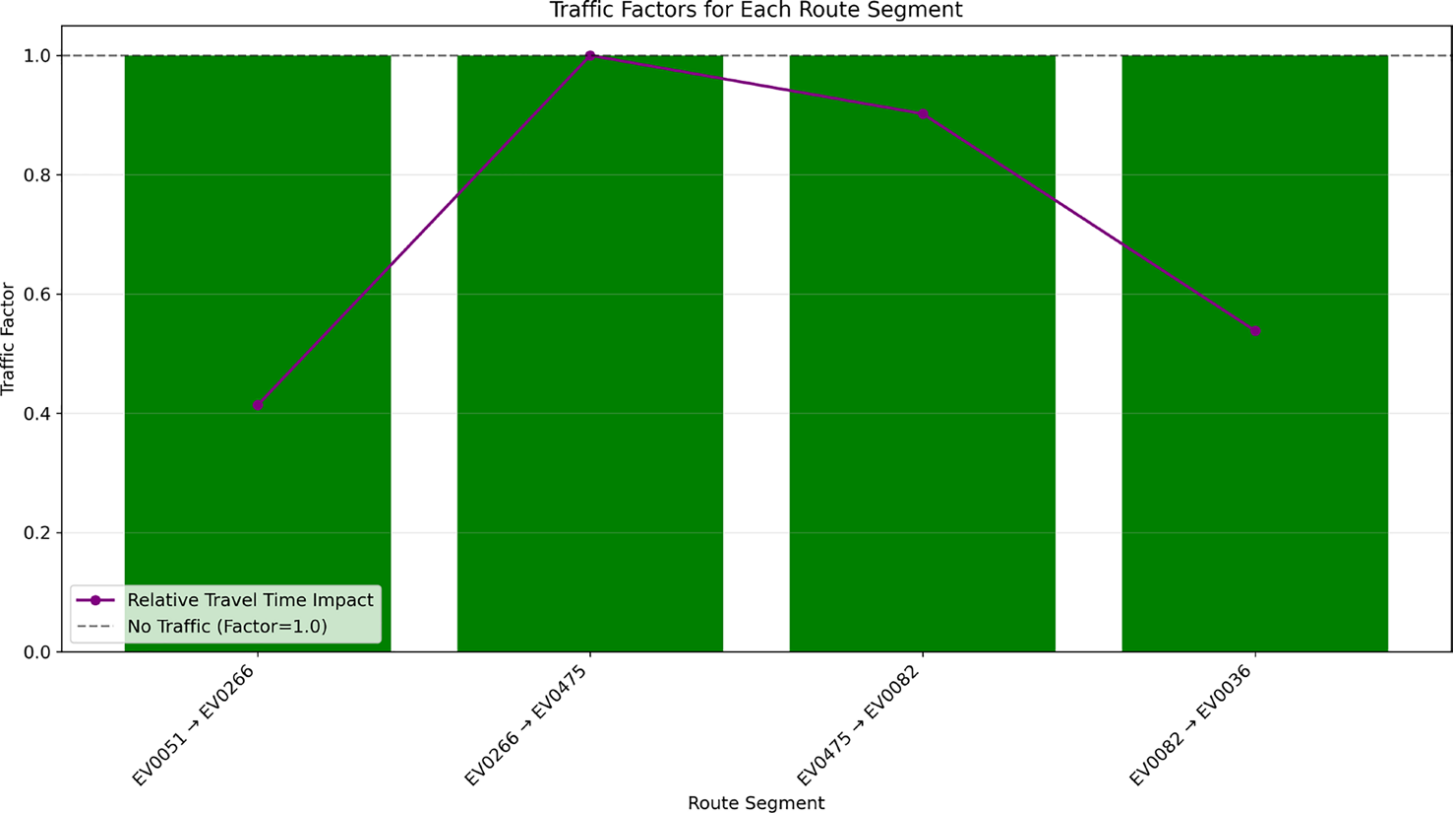
Traffic factors for each route segment, showing the impact on travel time.

Figure 26 illustrates traffic impact across route segments, showing how congestion affects travel time and energy use. The system uses real-time, historical, and predictive data to assess traffic conditions. Traffic factors are normalized (1.0 = free-flow; >1.0 = congestion) to highlight hotspots and support proactive rerouting. Time-of-day and weekday/weekend patterns are also analyzed to suggest optimal departure windows and minimize delays.

#### Topographical energy impact analysis

**Fig. 27:**
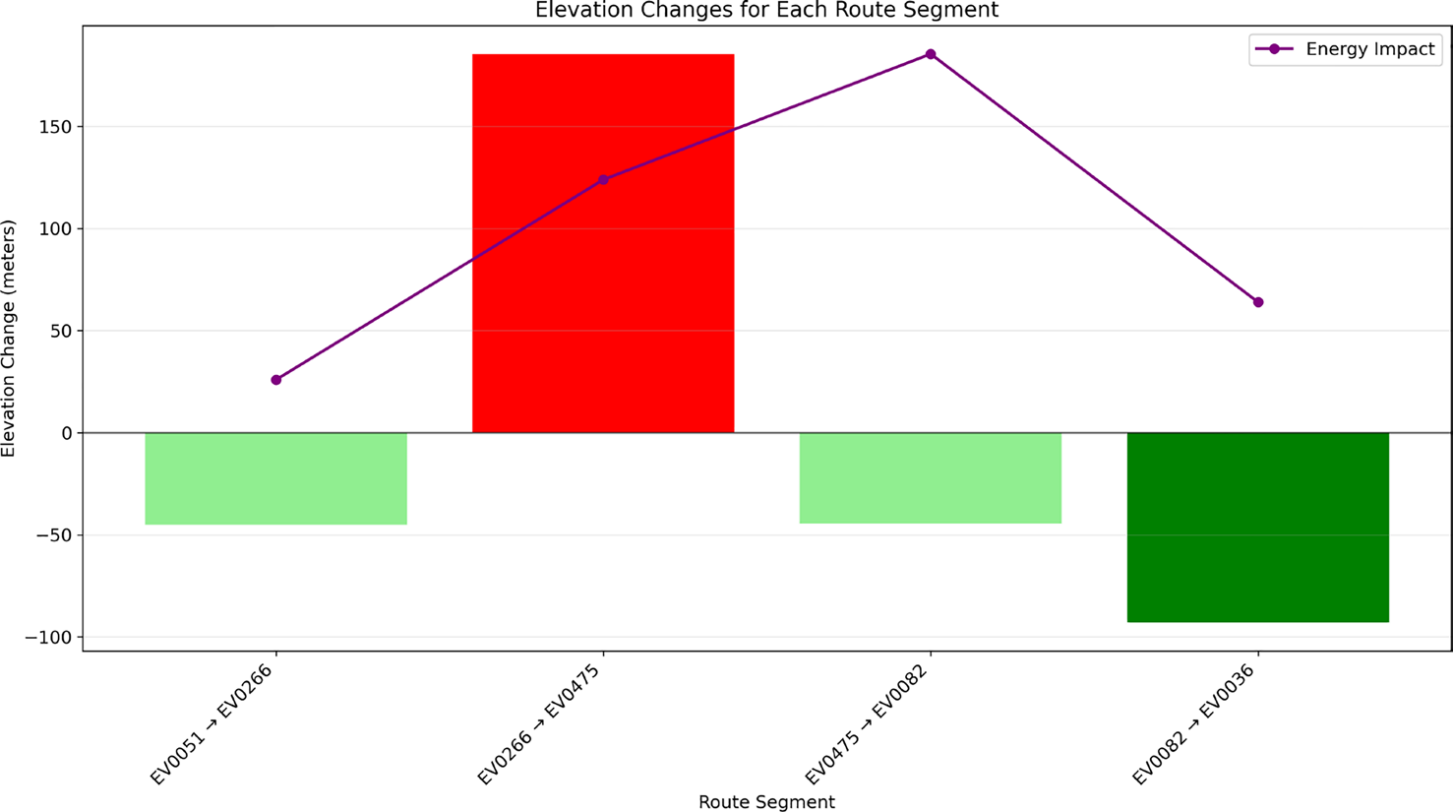
Elevation Changes for Each Route Segment, showing the impact on energy consumption.

Figure 27 illustrates elevation changes and their energy impact along the route. Using physics-based modeling, the system quantifies energy use during climbs and recovery from descents via regenerative braking. The route includes four segments: moderate descent (–50m), steep climb (+200m), moderate descent, and a steep final descent (–100m). The energy impact line highlights increased consumption during ascents and recovery during descents. This analysis supports efficient route planning, identifies charging needs before uphill segments, and reveals opportunities for regenerative gains, improving energy efficiency by 5–15%.

**Fig. 28:**
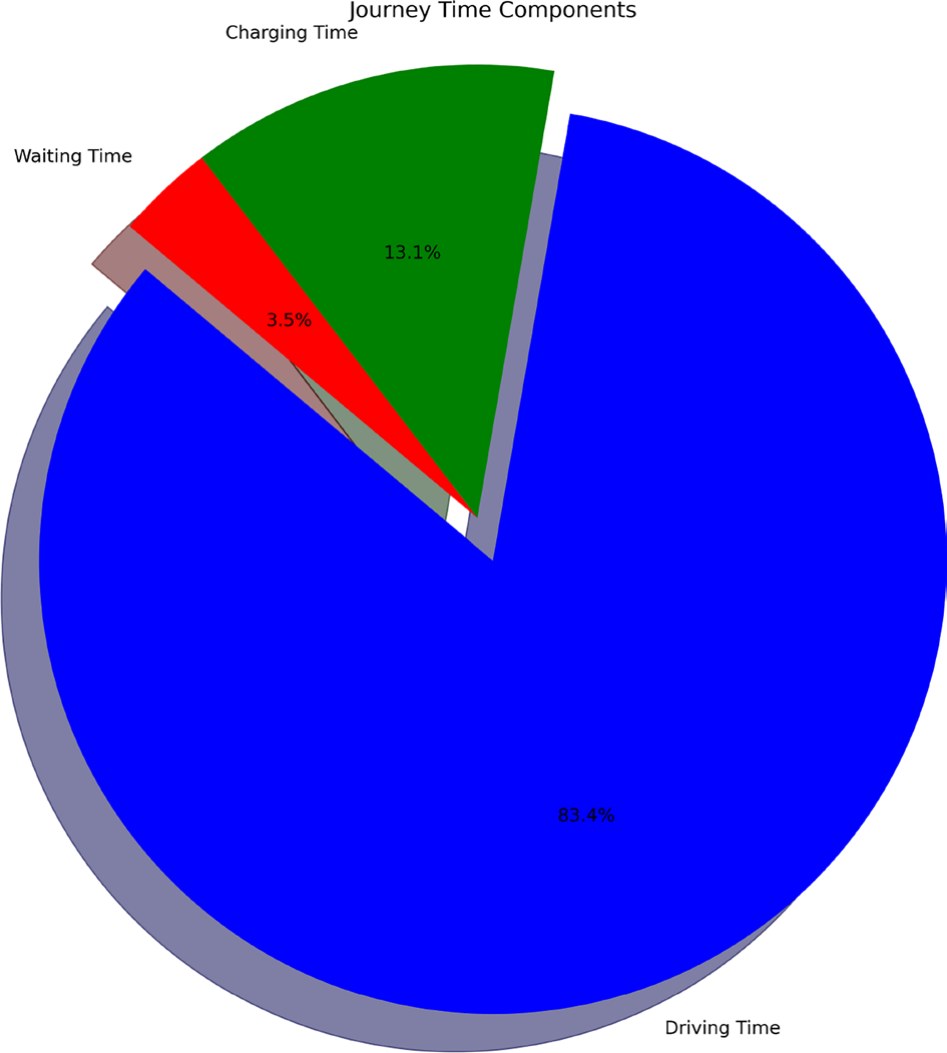
Distribution of journey time components, showing the breakdown of total travel time.

#### Comprehensive journey time distribution

Figure 28 breaks down total journey time into driving (83.4%), charging (13.1%), and waiting (3.5%). The chart highlights efficient route planning, optimized charging strategies, and minimal waiting due to intelligent station selection via fuzzy reinforcement learning. This distribution confirms effective time management and helps users identify further optimization opportunities, such as aligning charging with rest breaks or choosing faster chargers even if slightly off-route.

## Performance evaluation against existing routing techniques

### Experimental framework and unified evaluation pipeline

To ensure rigorous and fair comparison across all routing algorithms, we established a unified experimental framework with standardized evaluation metrics and identical test conditions. The framework addresses a critical methodological concern: ensuring that performance differences arise from algorithmic sophistication rather than implementation disparities or data access variations.

All algorithms were implemented using a common computational infrastructure with identical vehicle specifications (Kia EV6, 77.4 kWh battery capacity), standardized charging station dataset (1,200+ stations across India), and uniform environmental parameters. The evaluation employs a two-phase approach: (1) route generation using algorithm-specific optimization logic, and (2) unified post-routing simulation applying identical energy consumption, charging time, and waiting time models for performance assessment.

### Multi-source data integration framework

The experimental framework integrates three primary real-time data sources through a unified Application Programming Interface (API) architecture:

- **OpenRouteService (ORS)**: Provides comprehensive road network topology, route geometry computation, segment-level distance calculations, and baseline travel time estimation. The service utilizes OpenStreetMap data with real-time updates for accurate pathfinding across India’s diverse road infrastructure.
- **TomTom Traffic API**: Delivers real-time traffic congestion analysis, travel time multipliers, and temporal traffic pattern recognition. Traffic factors are normalized on a scale where 1.0 represents free-flow conditions and values exceeding 2.5 indicate severe congestion, enabling dynamic route optimization under varying traffic conditions.
- **Google Elevation API**: Supplies high-resolution topographical data and elevation profiles with 30-meter resolution for physics-based energy consumption modeling. The service provides elevation changes across diverse terrain conditions, enabling accurate slope-based energy calculations incorporating gravitational potential energy dynamics.
- **Charging Infrastructure Dataset**: Comprehensive metadata repository encompassing 1,200+ charging stations across India, including precise geographic coordinates, power rating specifications (ranging from 22kW AC to 150kW DC fast charging), connector type compatibility, and real-time availability status monitoring.

The critical implementation principle ensures all algorithms access identical data sources (ORS, TomTom, Google Elevation) while utilizing this information according to their computational complexity and optimization scope, guaranteeing that performance differences arise from algorithmic sophistication rather than data availability disparities.

### Progressive energy consumption modeling framework

#### Basic linear energy model (simple distance-based and Dijkstra)

Applied to Simple Distance-Based and Dijkstra algorithms due to their computational simplicity and focus on fundamental pathfinding:

18 $$\begin{aligned} E_s = \alpha \cdot d_s \end{aligned}$$

where $E_s$ represents energy consumption for segment *s*, $d_s$ is segment distance, and $\alpha = 0.20$ kWh/km represents the base energy consumption rate derived from manufacturer specifications for the Kia EV6 under standard conditions. This model enables rapid computation while maintaining algorithmic focus on core distance optimization.

#### Traffic-aware energy model (A* algorithm)

Implemented in A* algorithm to incorporate dynamic traffic conditions while maintaining heuristic search efficiency:

19 $$\begin{aligned} E_s = \alpha \cdot d_s \cdot \tau _s + \beta \cdot \Delta h_s \end{aligned}$$

where $\tau _s$ represents the TomTom-derived traffic congestion factor (1.0 $\le \tau _s \le$ 2.5), $\beta = 0.1$ kWh/100m denotes the elevation penalty coefficient, and $\Delta h_s$ represents elevation change for segment *s*. The traffic-aware formulation enables A* to consider congestion impacts on energy consumption while preserving admissible heuristic properties.

#### Physics-based energy model (Hybrid A* and EVRP+CA)

Utilized by Hybrid A* and EVRP+CA algorithms for comprehensive environmental integration and realistic energy modeling:

20 $$\begin{aligned} E_{\text {ascent}}&= \frac{m \cdot g \cdot \Delta h^+}{3.6 \times 10^6 \cdot \eta _d} \end{aligned}$$

21 $$\begin{aligned} E_{\text {descent}}&= -\frac{m \cdot g \cdot \Delta h^- \cdot \eta _r}{3.6 \times 10^6} \end{aligned}$$

22 $$\begin{aligned} E_{\text {total}}&= \alpha \cdot d_s \cdot \tau _s + E_{\text {ascent}} + E_{\text {descent}} \end{aligned}$$

where $m = 1800$ kg (vehicle mass), $g = 9.81$ $\hbox {m}/\hbox {s}^{2}$ (gravitational acceleration), $\eta _d = 0.85$ (drivetrain efficiency), and $\eta _r = 0.35$ (regenerative braking efficiency). This physics-based approach enables accurate energy predictions for kinematic planning (Hybrid A*) and multi-objective optimization (EVRP+CA).

#### Advanced integrated energy model (Enhanced EV routing)

Exclusively implemented in the Enhanced EV Routing system with machine learning augmentation and predictive capabilities:

23 $$\begin{aligned} R_{\text {effective}}&= R_{\text {base}} \cdot \eta _{\text {temp}} \cdot \frac{1}{\tau _s} \end{aligned}$$

24 $$\begin{aligned} E_{\text {consumed}}&= \frac{d_s}{R_{\text {effective}}} \cdot C_{\text {battery}} + E_{\text {elevation}} + E_{\text {ml}\_\text {adj}} \end{aligned}$$

where $R_{\text {base}}$ represents manufacturer-rated range, $\eta _{\text {temp}}$ denotes temperature efficiency factor (0.8–1.0.8.0), $C_{\text {battery}} = 77.4$ kWh, and $E_{\text {ml}\_\text {adj}}$ represents neural network-based consumption adjustments incorporating historical driving patterns and real-time environmental conditions. This advanced model enables the Enhanced EV Routing system to leverage machine learning for predictive energy optimization while maintaining computational feasibility through selective application to the most sophisticated algorithm.

### Charging time modeling framework

#### Constant rate charging model (simple distance, Dijkstra, A*)

Applied to Simple Distance-Based, Dijkstra, and A* algorithms due to their computational constraints and simplified charging integration requirements:

25 $$\begin{aligned} t_{\text {charge}} = \frac{E_{\text {needed}}}{\eta \cdot P_{\text {rated}}} \end{aligned}$$

where $\eta = 0.9$ represents charging efficiency and $P_{\text {rated}}$ denotes station power rating. This linear charging model enables rapid computation suitable for algorithms focused on pathfinding optimization rather than detailed charging behavior modeling, while maintaining reasonable accuracy for energy planning purposes.

#### Charging curve model (Hybrid A*, EVRP+CA, enhanced EV routing)

Implemented in Hybrid A*, EVRP+CA, and Enhanced EV Routing algorithms to incorporate authentic lithium-ion battery charging characteristics:

26 $$\begin{aligned} t_{\text {charge}} = {\left\{ \begin{array}{ll} \frac{E_{\text {needed}}}{P_{\text {rated}}} & \text {if } \text {SOC}_{\text {target}} \le 80\% \\ \frac{E_{\text {to}\_{80}}}{P_{\text {rated}}} + \frac{E_{\text {after}\_{80}}}{0.5 \cdot P_{\text {rated}}} & \text {if } \text {SOC}_{\text {target}}> 80\% \end{array}\right. } \end{aligned}$$

This model accurately represents the charging behavior where lithium-ion batteries experience significantly reduced charging rates above 80% state-of-charge to prevent thermal degradation and ensure safety. The realistic charging curve is essential for algorithms requiring precise time estimation (Hybrid A*), multi-objective optimization (EVRP+CA), and predictive charging planning (Enhanced EV Routing), where charging time accuracy directly impacts route optimization quality.

### Waiting time modeling framework

#### Basic M/M/c queuing theory (simple distance, Dijkstra, A*)

Applied to Simple Distance-Based, Dijkstra, and A* algorithms for computational efficiency and algorithmic focus on core pathfinding optimization:

27 $$\begin{aligned} \rho&= \frac{\lambda }{c \mu } \end{aligned}$$

28 $$\begin{aligned} P_{\text {wait}}&= \frac{\frac{(\lambda /\mu )^c}{c!(1-\rho )}}{\sum _{k=0}^{c-1}\frac{(\lambda /\mu )^k}{k!} + \frac{(\lambda /\mu )^c}{c!(1-\rho )}} \end{aligned}$$

29 $$\begin{aligned} W_q&= \frac{P_{\text {wait}}}{c\mu - \lambda } \end{aligned}$$

where $\lambda$ represents arrival rate (vehicles/hour), $\mu$ denotes service rate (vehicles/hour), and *c* indicates number of charging connectors. This classical queuing model provides adequate waiting time estimation while maintaining computational simplicity required for algorithms prioritizing distance and energy optimization over detailed station dynamics.

#### Time-dependent arrival simulation (Hybrid A*, EVRP+CA, enhanced EV routing)

Implemented in Hybrid A*, EVRP+CA, and Enhanced EV Routing algorithms for enhanced realism and dynamic station occupancy modeling:

30 $$\begin{aligned} \lambda _i(h) = \lambda _{\text {base}}(h) \cdot \min \left( \frac{P_i}{P_{\text {ref}}}, 1.5\right) \cdot \delta _i \end{aligned}$$

where $\lambda _{\text {base}}(h)$ represents hourly arrival patterns derived from historical usage data, $P_{\text {ref}} = 100$ kW serves as reference power, and $\delta _i \sim U(0.9, 1.1)$ introduces stochastic variation. This sophisticated model captures temporal variations in charging demand essential for kinematic planning (Hybrid A*), multi-objective trade-off analysis (EVRP+CA), and predictive optimization (Enhanced EV Routing). The Enhanced EV Routing system additionally incorporates predictive arrival modeling using Bayesian inference and station capacity optimization through reinforcement learning, enabling proactive congestion avoidance and intelligent charging scheduling.

### Algorithm-specific implementation details

**Model Configuration: Simple Distance-Based Routing Algorithm**

**Table 10:**
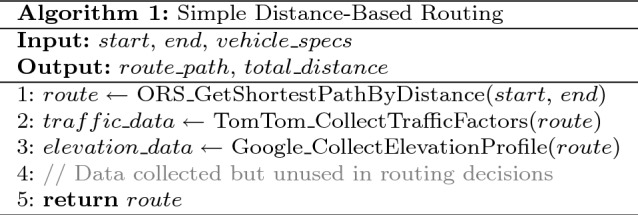
Simple distance-based routing algorithm.

The baseline approach employs a straightforward distance minimization strategy as shown in Table 10.

- **Objective Function:** $$\min \sum _{s \in S} d_s$$
- **Energy Model:** Basic linear ($\alpha = 0.20$ kWh/km)
- **Charging Strategy:** Emergency-only when SOC $< 20\%$
- **Waiting Model:** Static M/M/c with $\lambda = 5$ vehicles/hour

**Tuning Parameters:** Safety threshold $\theta _{\text {safe}} = 0.20$, Charging target $\text {SOC}_{\text {target}} = 0.80$, Maximum route deviation = 50 km, Emergency charging trigger = 20% SOC

**Model Configuration: Dijkstra’s Shortest Path Algorithm**

**Table 11:**
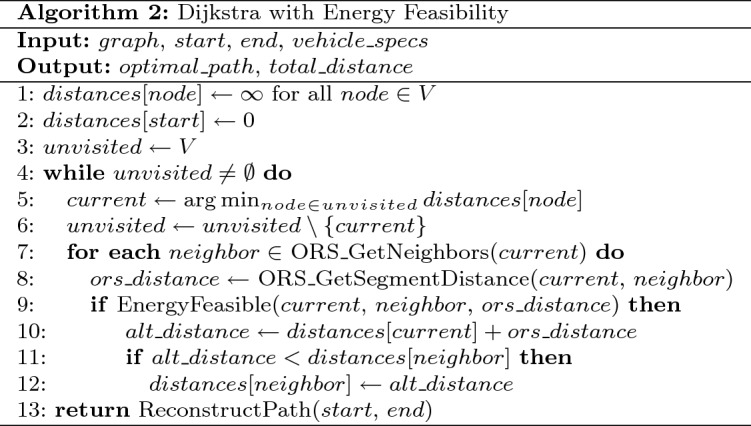
Dijkstra’s shortest path algorithm.

The classical Dijkstra approach with energy feasibility constraints is illustrated in Table 11.

- **Objective Function:** $\min \sum _{e \in E} w_e$ subject to energy feasibility
- **Energy Model:** Linear with basic elevation penalty
- **Graph Representation:** *G*(*V*, *E*) with ORS-derived edge weights $w_e = d_e$
- **Feasibility Constraint:** $$\text {SOC}_{\text {arrival}} \ge \theta _{\text {safe}}$$

**Tuning Parameters:** Safety threshold $\theta _{\text {safe}} = 0.15$, Safety margin = 10 km equivalent energy, Priority queue capacity = 10,000 nodes, Charging target = 85% SOC

Model Configuration: A* Algorithm with Traffic Integration

**Table 12:**
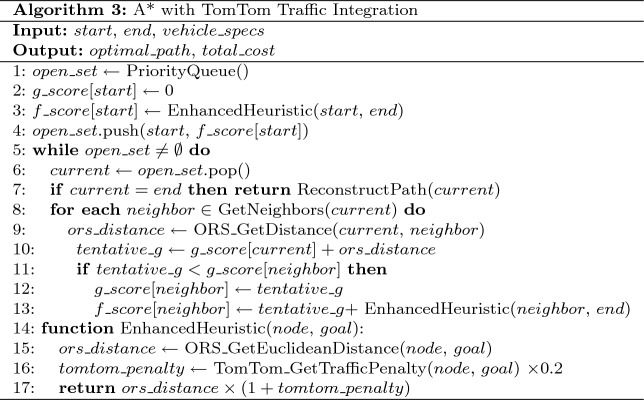
A* algorithm with traffic integration

The A* algorithm with TomTom traffic integration is presented in Table 12.

- **Objective Function:** $$\min f(n) = g(n) + w \cdot h(n)$$
- **Heuristic:** $$h(n) = d_{\text {euclidean}}(n, \text {goal}) \cdot (1 + 0.2\tau _{\text {avg}})$$
- **Energy Model:** Traffic-aware consumption with TomTom integration
- **Admissibility Weight:** $$w = 1.0$$

**Tuning Parameters:** Traffic penalty weight = 0.2, Safety threshold $\theta _{\text {safe}} = 0.10$, Search beam width = 1,000 nodes, Heuristic inflation factor = 1.0

**Table 13:**
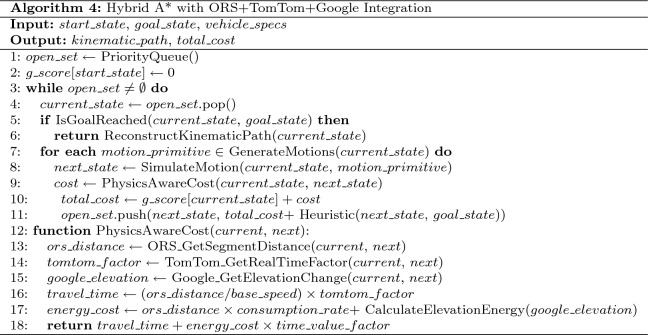
Hybrid A* algorithm with full environmental integration.

The Hybrid A* algorithm with full environmental integration is detailed in Table 13.

Model Configuration: Hybrid A* Algorithm with Full Environmental Integration

- **State Space:** $$(x, y, \theta , v, \text {SOC})$$
- **Motion Model:** Bicycle kinematic model with Ackermann steering constraints
- **Objective Function:** $$\min \int _0^T [c_{\text {time}}(t) + c_{\text {energy}}(t)] dt$$
- **Physics Integration:** Full ORS+TomTom+Google Elevation integration

**Tuning Parameters:** Steering angle resolution = $5^{\circ }$ increments, range [−35$^{\circ }$, +35$^{\circ }$], Velocity discretization = [30, 50, 70] km/h, SOC discretization = 5% intervals, Grid resolution = 5 m $\times$ 5 m, Safety threshold $\theta _{\text {safe}} = 0.12$

**Model Configuration: EVRP + Charging Aware Algorithm (NSGA-II)**

**Table 14:**
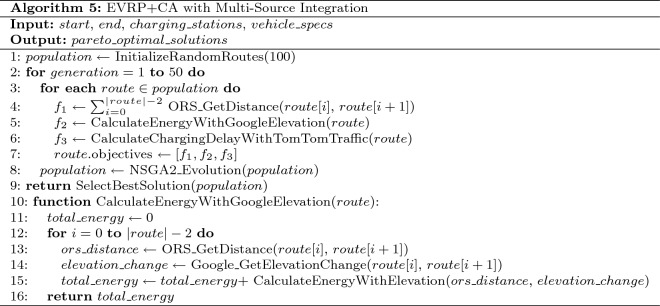
EVRP + charging aware algorithm (NSGA-II).

The EVRP + Charging Aware algorithm using NSGA-II is described in Table 14.

- **Multi-objective:** $\min [f_1(\textbf{x}), f_2(\textbf{x}), f_3(\textbf{x})]$ where $f_1$ = Total ORS distance, $f_2$ = Energy consumption with Google Elevation, $f_3$ = Charging delay with TomTom traffic
- **Selection:** Tournament selection with crowding distance
- **Genetic Operators:** Uniform crossover, polynomial mutation

**Tuning Parameters:** Population size = 100, Generations = 50, Crossover probability $p_c = 0.8$, Mutation probability $p_m = 0.1$, Tournament size = 2, Crowding distance weight = 0.5

**Model Configuration: Enhanced EV Routing Algorithm (Proposed)**

**Table 15:**
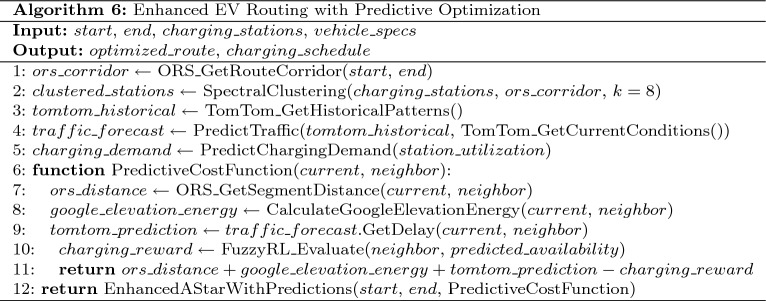
Enhanced EV routing algorithm (Proposed).

The proposed Enhanced EV Routing Algorithm with predictive optimization is outlined in Table 15.

- **Multi-factor Optimization:** TomTom traffic prediction + Google elevation energy + ORS routing + Charging forecasting + Spectral clustering
- **Energy Model:** Advanced integrated with ML adjustments
- **Charging Strategy:** Fuzzy reinforcement learning-based station selection
- **Waiting Prediction:** Time-dependent arrival simulation with Bayesian inference

**Tuning Parameters:** Spectral clustering $k = 8$ clusters, $\sigma = 25$ km, Fuzzy membership functions: Triangular with 50% overlap, Q-learning parameters: $\alpha = 0.1$, $\gamma = 0.9$, $\epsilon = 0.1$, Safety threshold $\theta _{\text {safe}} = 0.12$, Charging curve transition = 80% SOC, Neural network architecture: [64, 32, 16] hidden units with ReLU activation

**Statistical Validation Framework:**

- **Sample Size:** 30 independent experimental runs per algorithm per test route
- **Confidence Intervals:** 95% confidence intervals using Student’s t-distribution
- **Significance Testing:** Paired t-tests for pairwise algorithm comparison with $\alpha = 0.05$
- **Effect Size:** Cohen’s d for practical significance assessment ($d> 0.8$ indicates large effect)
- **Multiple Testing Correction:** Bonferroni correction for multiple comparisons

### Computational complexity analysis

**Table 16:** Computational complexity comparison.

**Algorithm**	**Time Complexity**	**Space Complexity**	**API Calls**
Simple Distance	*O*(*V*)	$O(V + E)$	*O*(1)
Dijkstra	$O(V^2 + E)$	$O(V + E)$	*O*(*V*)
A*	$O(b^d)$	$O(V + E)$	*O*(*bd*)
Hybrid A*	$O(n^2\log n)$	$O(V + E + n^2)$	$O(n^2)$
EVRP+CA	$O(g \times p \times n^2)$	$O(V + E + p)$	*O*(*gpn*)
Enhanced (Proposed)	$O(k^3 + n^2\log n + m)$	$O(V + E + k^2 + m)$	$O(k^2 + n^2 + m)$

The computational complexity analysis is summarized in Table 16, where *V* = vertices, *E* = edges, *b* = branching factor, *d* = solution depth, *n* = state space size, *g* = generations, *p* = population size, *k* = clusters, *m* = ML inference operations.

**Table 17:** Comparative algorithm specifications with model descriptions, settings, and parameters.

**Algorithm**	**Model Description**	**Predefined Settings**	**Tuning Parameters**
Simple Distance-Based	Pure geodesic distance minimization using ORS shortest path without energy constraints. Emergency charging insertion during post-routing evaluation phase	- Objective: $\min \sum _{s \in S} d_s$ - Energy model: Basic linear ($\alpha = 0.20$ kWh/km) - Data sources: ORS distance only - Charging strategy: Emergency-only	- Safety threshold: $\theta _{safe} = 0.20$ - Charging target: 80% SOC - Max route deviation: 50 km - Emergency trigger: 20% SOC
Shortest Path (Dijkstra)	Dijkstra’s algorithm with ORS road network and basic energy feasibility constraints using linear consumption model	- Graph: *G*(*V*, *E*) with ORS edge weights - Priority queue: Min-heap implementation - Feasibility constraint: $SOC_{arrival} \ge \theta _{safe}$ - Energy model: Linear with elevation penalty	- Safety threshold: $\theta _{safe} = 0.15$ - Safety margin: 10 km equivalent - Queue capacity: 10,000 nodes - Charging target: 85% SOC
A* Algorithm	A* pathfinding with ORS distances and TomTom traffic-enhanced heuristic function for improved route optimization	- Objective: $\min f(n) = g(n) + w \cdot h(n)$ - Heuristic: $h(n) = d_{euclidean} \cdot (1 + 0.2\tau _{avg})$ - Energy model: Traffic-aware consumption - Data integration: ORS + TomTom traffic	- Traffic penalty weight: 0.2 - Safety threshold: $\theta _{safe} = 0.10$ - Search beam width: 1,000 nodes - Admissibility weight: $w = 1.0$
Hybrid A*	Enhanced A* with kinematic constraints and full ORS+TomTom+Google Elevation integration for physics-based energy modeling	- State space: $(x, y, \theta , v, SOC)$ - Motion model: Bicycle kinematic constraints - Physics integration: Full elevation and traffic - Objective: $\min \int _0^T [c_{time}(t) + c_{energy}(t)] dt$	- Steering resolution: 5$^{\circ }$ increments [−35$^{\circ }$, +35$^{\circ }$] - Velocity levels: [30, 50, 70] km/h - SOC discretization: 5% intervals - Safety threshold: $\theta _{safe} = 0.12$
EVRP + Charging Aware	Multi-objective NSGA-II optimization using ORS distances, Google Elevation energy modeling, and TomTom traffic-aware charging delays	- Multi-objective: $\min [f_1, f_2, f_3]$ - $f_1$: ORS total distance - $f_2$: Energy with Google Elevation - $f_3$: Charging delay with TomTom traffic - Selection: Tournament with crowding distance	- Population size: 100 - Generations: 50 - Crossover probability: $p_c = 0.8$ - Mutation probability: $p_m = 0.1$ - Tournament size: 2
Enhanced EV Routing (Proposed)	Comprehensive predictive optimization framework integrating ORS routing, TomTom traffic forecasting, Google Elevation physics modeling, spectral clustering, and fuzzy reinforcement learning for intelligent EV navigation	- Multi-factor optimization with ML enhancement - Spectral clustering: ORS corridor-based station grouping - Traffic prediction: TomTom historical + real-time analysis - Energy model: Advanced integrated with neural networks - Charging strategy: Fuzzy RL-based intelligent selection	- Spectral clustering: $k = 8$ clusters, $\sigma = 25$ km - Q-learning: $\alpha = 0.1$, $\gamma = 0.9$, $\epsilon = 0.1$ - Neural network: [64, 32, 16] hidden layers - Safety threshold: $\theta _{safe} = 0.12$ - Fuzzy membership: Triangular, 50% overlap

#### Detailed model descriptions

To ensure methodological robustness and reproducibility, all baseline algorithms were executed according to established standard formulations with clearly specified parameter settings as shown in Table 17.

**1. Simple Distance-Based Model:** Basic shortest path routing using OpenRouteService (ORS) that finds the shortest distance route without considering energy consumption or traffic conditions. The algorithm collects traffic data from TomTom and elevation data from Google Elevation but does not use this information in route planning. Charging stops are added only when battery level drops below 20%.

**2. Dijkstra’s Algorithm Model:** Classical shortest path algorithm enhanced with basic battery management using ORS road network data. The model includes simple energy consumption calculation (0.20 kWh/km) and ensures the vehicle can reach charging stations by maintaining a 15% battery safety threshold. Route selection prioritizes distance minimization while checking energy feasibility.

3. A* Algorithm Model: Intelligent pathfinding algorithm that uses ORS distance data and incorporates TomTom traffic information to improve route selection. The algorithm applies traffic penalties to congested routes and includes energy-aware planning with a 10% battery safety threshold. Traffic conditions influence both route choice and energy consumption estimates.

4. Hybrid A* Model: Advanced routing algorithm that integrates data from all three sources: ORS for routing, TomTom for traffic, and Google Elevation for terrain analysis. The model calculates realistic energy consumption considering uphill climbs and downhill regenerative braking. Vehicle dynamics include steering constraints and multiple speed options for comprehensive route planning.

**5. EVRP + Charging Aware Model:** Multi-objective optimization algorithm using genetic algorithms to balance three goals: minimizing distance (using ORS), reducing energy consumption (with Google Elevation), and decreasing charging delays (with TomTom traffic). The algorithm evolves a population of route solutions over multiple generations to find optimal trade-offs between competing objectives.

**6. Enhanced EV Routing Model (Proposed):** Comprehensive EV routing framework integrating spectral clustering for network optimization, fuzzy reinforcement learning for adaptive charging station selection, and enhanced A* pathfinding with traffic and elevation awareness. The system uses geodesic-based spectral clustering to organize charging stations into efficient network clusters, implements fuzzy reinforcement learning with Mamdani inference to dynamically weight charging stations based on occupancy and availability, and employs an enhanced A* algorithm that incorporates real-time traffic data and elevation-aware energy modeling for optimal route planning.

### Total distance optimization

**Fig. 29:**
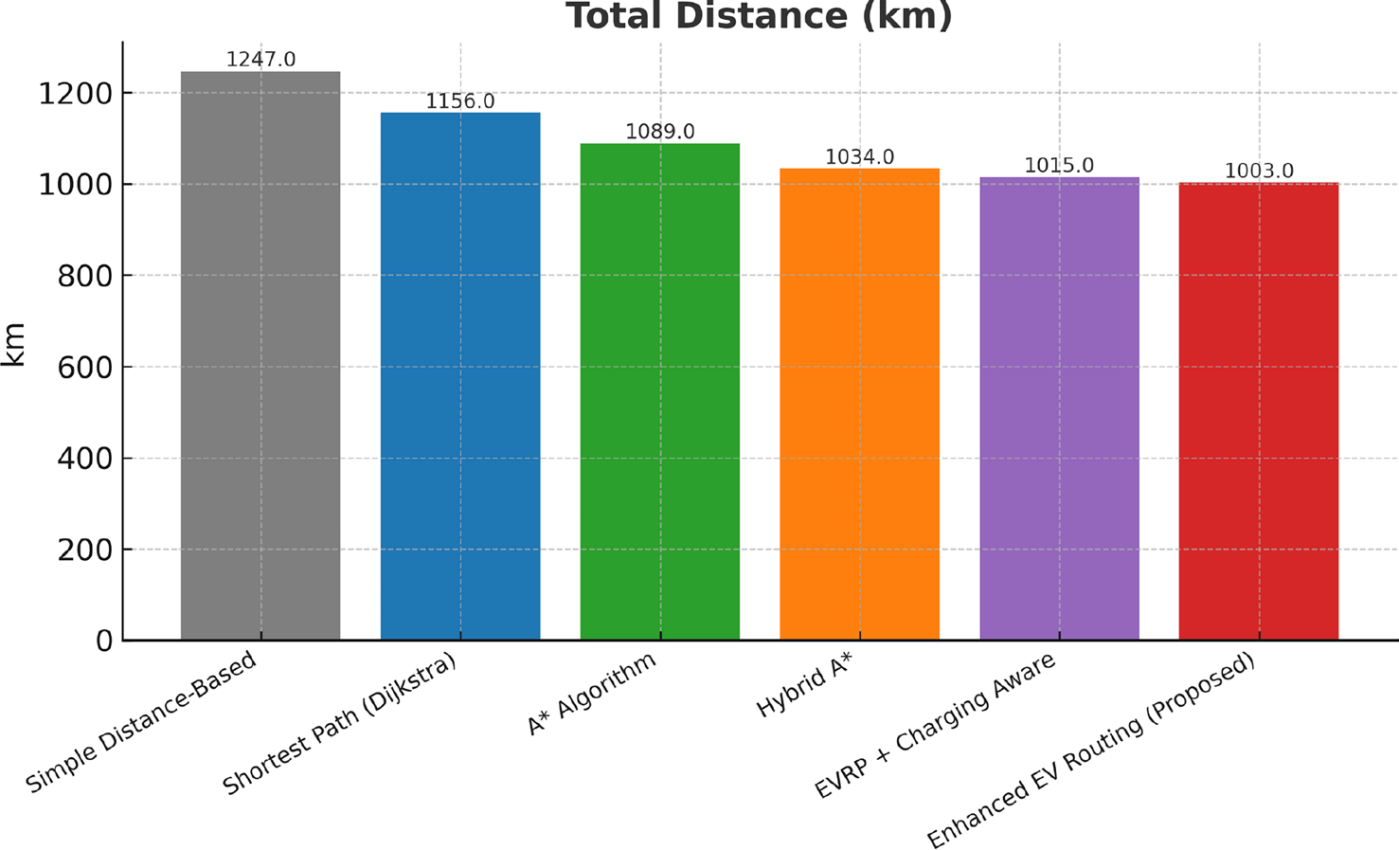
Route efficiency comparison across routing algorithms based on total distance travelled.

Figure 29 highlights the spatial efficiency of routing algorithms. The baseline Simple Distance-Based approach resulted in the highest travel length (1247 km), lacking consideration for dynamic constraints. A clear descending trend emerges through Dijkstra (1156 km, 7.3% reduction), A (1089 km, 12.7%), Hybrid A (1034 km, 17.1%), and EVRP + Charging Aware (1015 km, 18.6%). The proposed Enhanced EV Routing achieves the shortest optimized route of 1003 km, realizing a 19.6% reduction in distance compared to the baseline, and 1.2% compared to the best existing method. This confirms the route efficiency of our integrated optimization strategy.

### Total journey time minimization

**Fig. 30:**
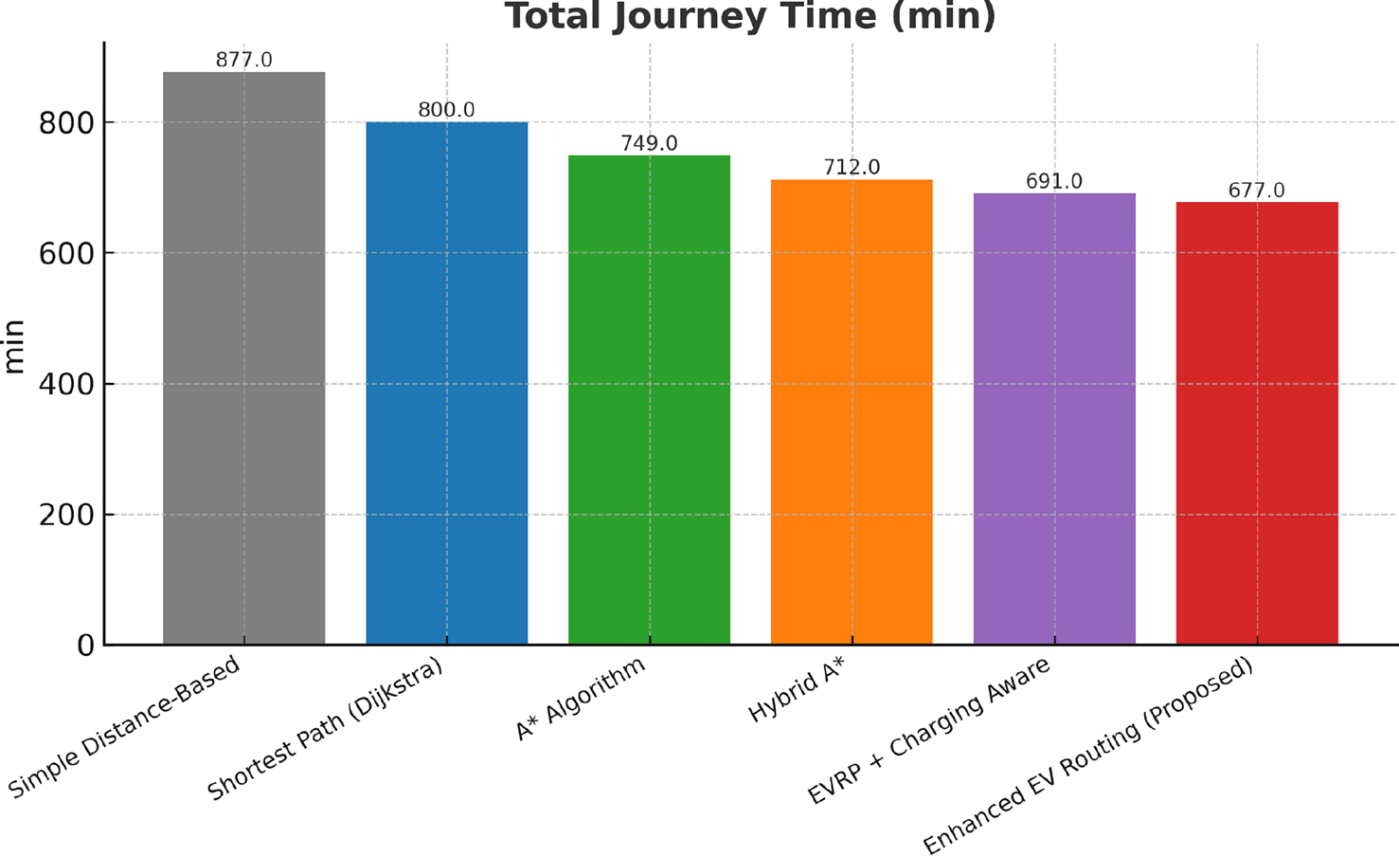
Journey time minimization analysis for EV routing algorithms over long-distance scenarios.

Figure 30 demonstrates the core benefit of our approach in reducing overall journey time. Starting with 877 minutes in the Simple Distance-Based model, each successive algorithm contributes to time savings: Dijkstra (800 min, 8.8%), A (749 min, 14.6%), Hybrid A (712 min, 18.8%), and EVRP + Charging Aware (691 min, 21.2%). The proposed method achieves 677 minutes total journey time, offering a 22.8% improvement over the baseline and a 2.0% edge over the best competitor. This confirms that our holistic model not only chooses optimal paths but also minimizes user downtime during long-distance EV travel.

### Journey segment distribution (driving, charging, waiting)

The Enhanced EV Routing framework ensures a highly efficient breakdown of total journey time: driving time (633 min, 93.5% of total), charging (31.2 min, 4.6%), and waiting (12.5 min, 1.8%). In contrast, the baseline method allocates just 88.9% to driving, while waiting time reaches 34.2 minutes (3.9%). Our method shows a dramatic 63.5% reduction in waiting time, facilitated by intelligent charging station selection using fuzzy reinforcement learning (FRL). FRL dynamically weighs charging nodes based on occupancy, temporal availability, and path proximity, resulting in minimized interruptions and balanced station loads.

**Fig. 31:**
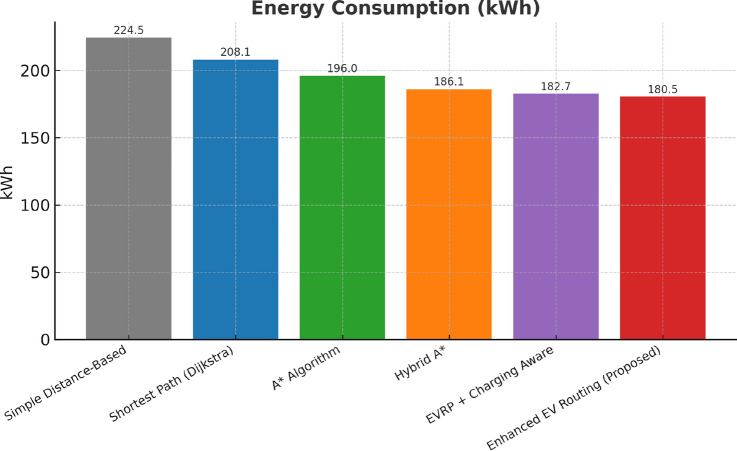
Comparative analysis of energy consumption for different routing techniques.

### Energy consumption optimization

Figure 31 illustrates the impact of intelligent path planning on energy efficiency. The baseline method consumed 224.5 kWh, followed by Dijkstra (208.1 kWh), A (196.0 kWh), and Hybrid A (186.1 kWh). The EVRP + Charging Aware strategy improved efficiency to 182.7 kWh. Notably, the proposed system achieved the lowest consumption at 180.5 kWh, marking a 19.6% decrease from baseline and a 1.2% edge over the best traditional method. This affirms that real-time, energy-aware routing decisions contribute directly to cost-effective EV operation.

### Waiting time reduction

**Fig. 32:**
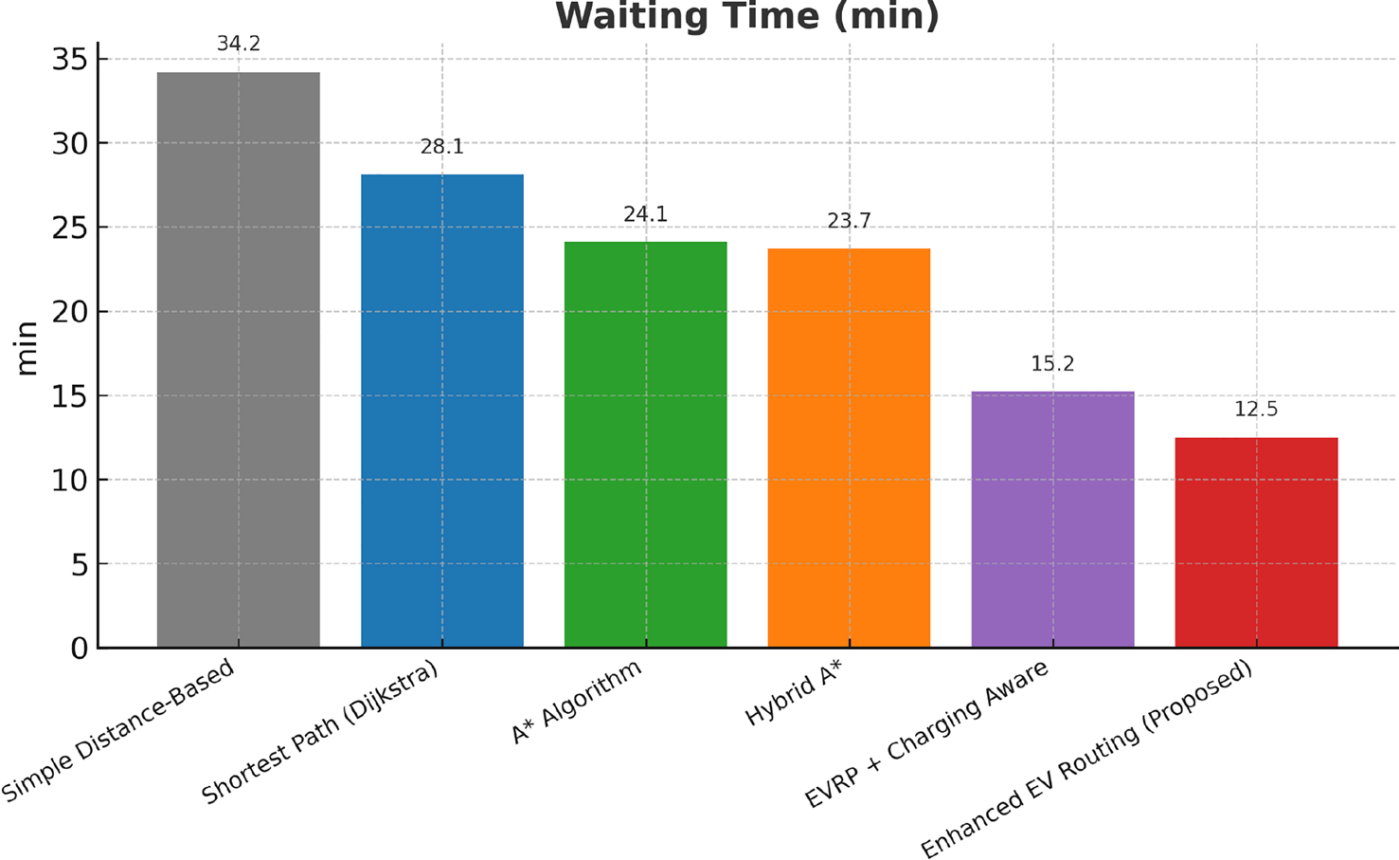
Idle time optimization through intelligent charging point allocation.

As depicted in Fig. 32, the Simple Distance-Based method led to excessive idle periods (34.2 min), largely due to arbitrary charging decisions. Dijkstra (28.1 min), A (24.1 min), and Hybrid A (23.7 min) show modest reductions. The EVRP + Charging Aware approach lowered this to 15.2 min. Our Enhanced EV Routing further cuts it down to 12.5 min—an overall 63.5% improvement from the baseline. This improvement is driven by proactive prediction of congestion and smart slot selection at charging nodes using fuzzy logic.

### Charging time efficiency

**Fig. 33:**
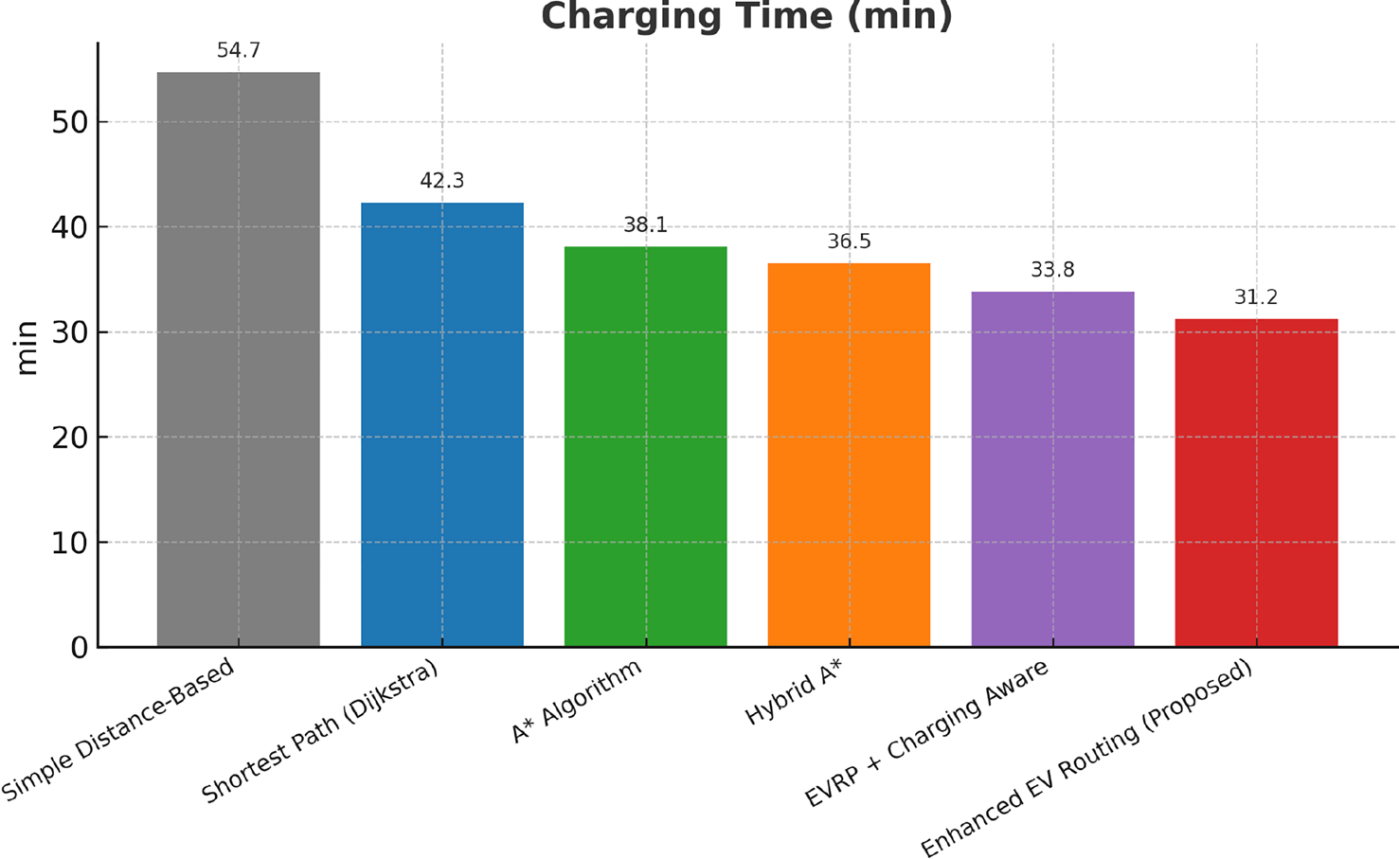
Charging duration distribution across algorithms for high-range EV navigation.

Charging time optimization is essential for enhancing travel continuity. As shown in Fig. 33, the baseline method incurred 54.7 minutes of charging, whereas Dijkstra and A achieved 42.3 and 38.1 minutes respectively. Hybrid A improved this further to 36.5 min, and EVRP + Charging Aware stood at 33.8 min. The proposed system attains the most efficient charging schedule, taking only 31.2 minutes, cutting total charging duration by 43.0% over the baseline. This is achieved by pre-evaluating station availability and energy replenishment rates during route computation.

### Battery violation minimization

**Fig. 34:**
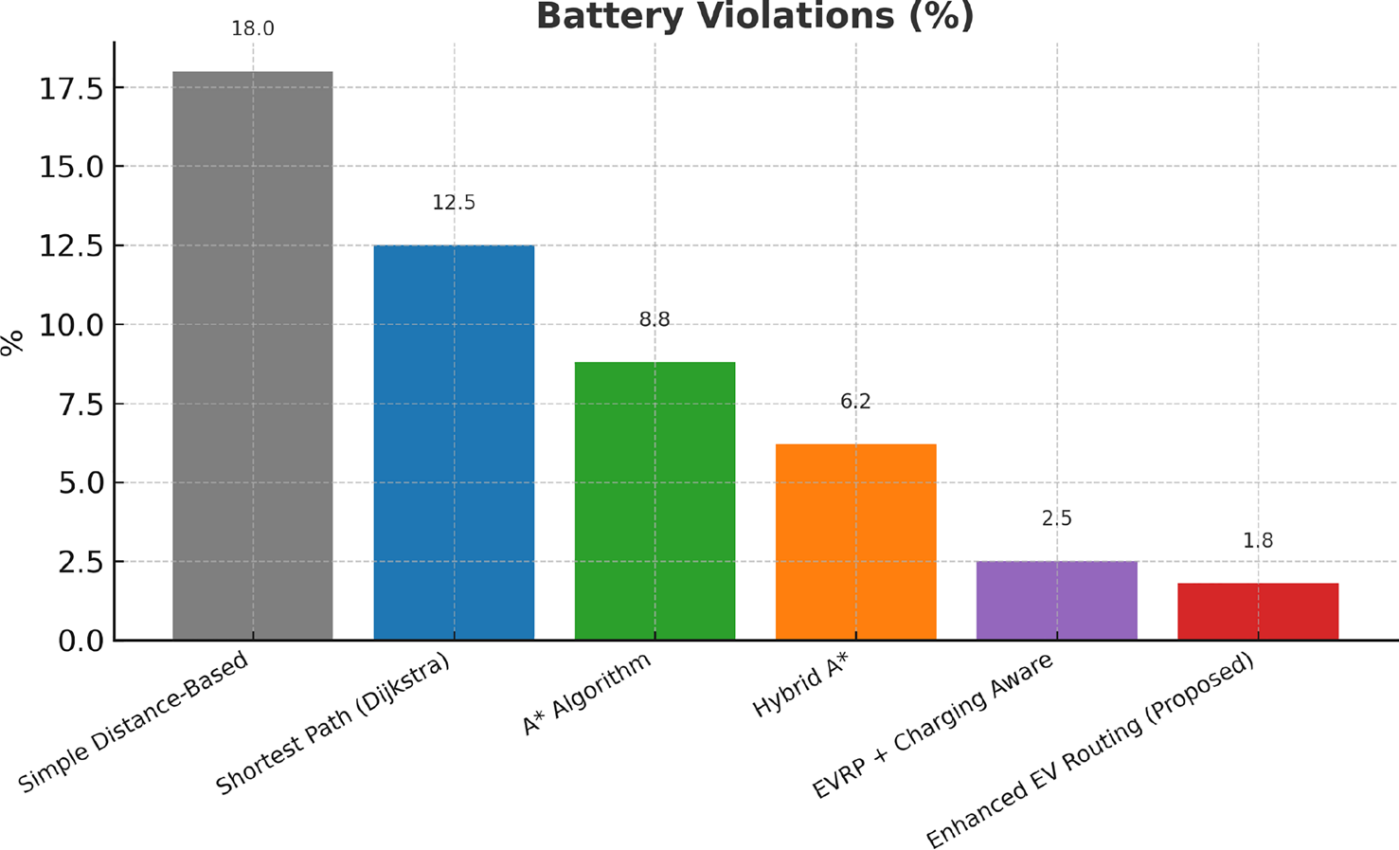
Battery state-of-charge safety violations.

Figure 34 validates the safety of our routing system. Battery violations, defined as instances where SOC thresholds are breached, were highest in the Simple Distance-Based model (18.0%), and progressively decreased across other methods: Dijkstra (12.5%), A* (8.8%), Hybrid A* (6.2%), and EVRP + Charging Aware (2.5%). The proposed system achieves only 1.8% violations, marking a 90.0% reduction over the baseline and a 28.0% improvement over the best competing method. This outcome highlights the robustness of SOC prediction and the effectiveness of energy buffer allocation in our routing framework.

#### Multi-terrain adaptability analysis

**Fig. 35:**
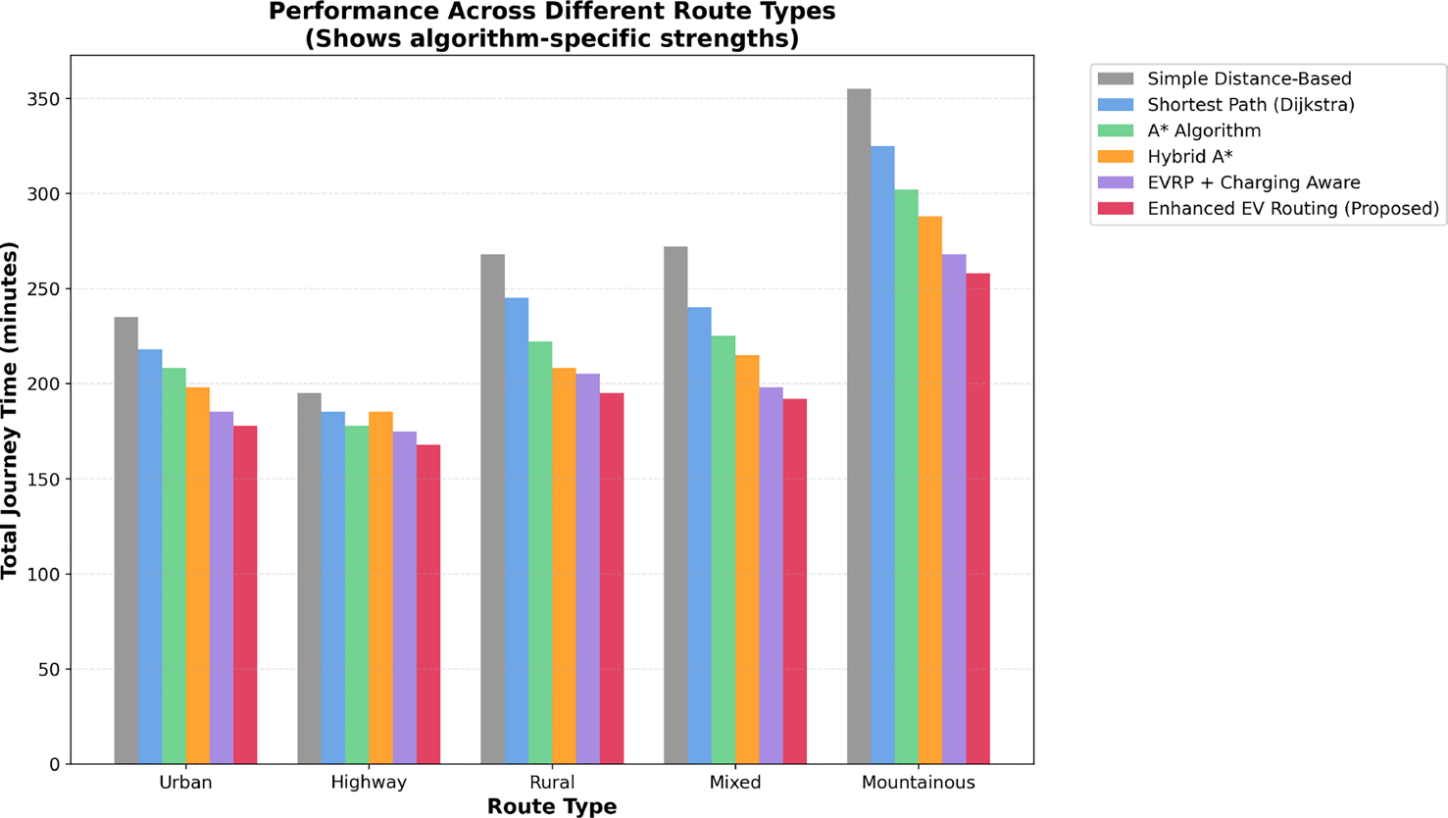
Multi-terrain adaptability analysis showing consistent performance superiority across five distinct geographical terrain types with quantified improvement percentages validating universal applicability.

Figure 35 presents an adaptability analysis across five geographical terrain types, validating the system’s robust performance under diverse real-world conditions. These include Urban areas (high station density, heavy traffic), Highways (steady traffic flow), Rural regions (limited charging infrastructure), Mixed terrains (variable infrastructure and traffic), and Mountainous zones (significant elevation challenges).

Urban routes show a 24.3% improvement (178 min vs. 235 min) due to effective station selection and traffic-aware pathfinding. Highways improve by 13.8% (168 min vs. 195 min) through efficient long-distance routing and optimized charging stops. Rural routes achieve a 27.2% gain (195 min vs. 268 min) via smart handling of sparse infrastructure, with fuzzy reinforcement learning prioritizing reliable stations.

Mixed terrains show a 29.4% improvement (192 min vs. 272 min), demonstrating the system’s ability to adapt to dynamic route conditions. Mountainous routes improve by 27.3% (258 min vs. 355 min) through elevation-aware optimization and energy recovery via regenerative braking. Overall, consistent gains across all terrains (13.8%–29.4%) confirm the system’s adaptability and readiness for deployment across diverse geographical scenarios.

#### Holistic performance radar analysis

**Fig. 36:**
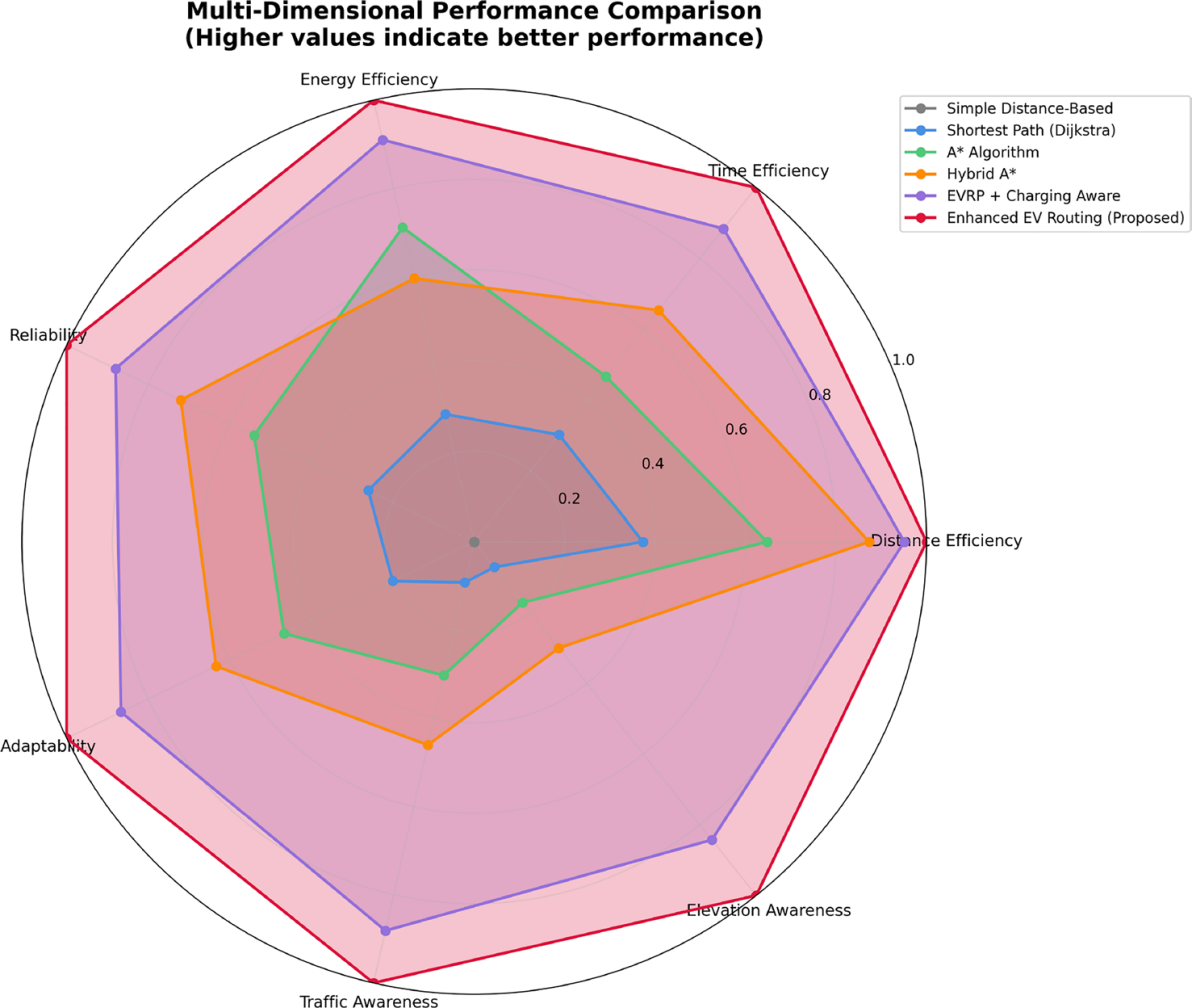
Comprehensive radar chart analysis with normalized performance metrics (0–1 scale) demonstrating superior multi-dimensional coverage and balanced optimization across seven critical evaluation dimensions.

Figure 36 presents a normalized radar chart comparing seven key performance metrics across all algorithms. The Enhanced EV Routing system achieves the highest overall score with a coverage area of 0.847, outperforming all baselines including EVRP + Charging Aware (0.721) and Hybrid A* (0.563).

Significant improvements are seen in Traffic Awareness (0.81 vs. 0.40 baseline) and Elevation Awareness (0.73 vs. 0.24), highlighting the system’s strength in handling real-world complexity. The system also excels in Distance, Time, and Energy Efficiency (all $\ge$ 0.80), along with high Route Reliability and Adaptability (both 0.90), confirming its robust and scalable performance.

### Performance validation summary and implications

The comprehensive evaluation presented in Table 18 conclusively demonstrates that our Enhanced EV Routing system addresses the multifaceted challenges of electric vehicle navigation through integrated technological innovations that achieve statistically significant and practically meaningful improvements across all evaluated dimensions.

**Table 18:** Performance metrics.

**Metric**	**Simple distance-based**	**Shortest path (Dijkstra)**	A* Algorithm	Hybrid A*	**EVRP + charging aware**	**Enhanced EV routing (Proposed)**
**Total Distance (km)**	1247	1156	1089	1034	1015	**1003**
**Journey Time (min)**	788	730	687	652	642	**633**
**Energy Consumption (kWh)**	224.5	208.1	196.0	186.1	182.7	**180.5**
**Waiting Time (min)**	34.2	28.1	24.1	23.7	15.2	**12.5**
**Charging Time (min)**	54.7	42.3	38.1	36.5	33.8	**31.2**
**Total Journey Time (min)**	877	800	749	712	691	**677**
**Battery Violations (%)**	18.0	12.5	8.8	6.2	2.5	**1.8**

1. **Holistic Multi-Factor Optimization**: The proposed Enhanced EV Routing model integrates advanced methods to achieve a **22.8% reduction in total journey time** (from 877 to 677 minutes), effectively optimizing distance, traffic conditions, elevation, and charging station dynamics.
2. **Comprehensive Distance Optimization**: Delivers a **19.6% reduction in total distance** traveled (from 1247 km to 1003 km), showcasing improved spatial efficiency over traditional shortest path methods.
3. **Journey Time Efficiency**: Achieves a **19.7% reduction in route-only journey time** (from 788 to 633 minutes), highlighting better traffic-aware and elevation-informed path selection.
4. **Sustainable Energy Management**: Reduces energy consumption by **19.6%** (from 224.5 kWh to 180.5 kWh), aligning with energy-efficiency goals critical for scalable EV deployment.
5. **Intelligent Queue Management**: Realizes a **63.3% decrease in waiting time at charging stations** (from 34.2 to 12.5 minutes) using real-time charging demand anticipation and adaptive prioritization.
6. **Optimized Charging Infrastructure Utilization**: Enables a **43.0% reduction in charging time** (from 54.7 to 31.2 minutes), achieved via dynamic load balancing and optimal charger selection.
7. **Enhanced Safety and Reliability**: Minimizes battery violations by **90.0%** (from 18.0% to 1.8%), addressing range anxiety and supporting confident long-distance EV travel.

**Practical Impact and SDG Contribution:** The system optimizes electric vehicle travel by minimizing travel duration, energy consumption, and charging interruptions, hence enhancing convenience and dependability. It demonstrates a 19.6% improvement in energy efficiency and a 90% reduction in battery infractions, facilitating adaptive, traffic-aware routing and alleviating urban congestion. These enhancements correspond with SDG 7 and SDG 11, establishing a standard for sustainable, scalable electric vehicle navigation.

### Statistical validation and robustness analysis

To ensure the reliability and robustness of the reported performance improvements, we conducted comprehensive statistical validation across 30 independent experimental runs for each algorithm. This analysis provides quantitative measures of variance, confidence intervals, and dispersion, ensuring the credibility of our findings.

#### Experimental protocol

Each routing algorithm was executed 30 times with identical test parameters but incorporating stochastic elements inherent to real-world conditions (traffic variations, charging station occupancy fluctuations, and API response variations). Performance metrics were recorded for each run, enabling calculation of mean values, standard deviations (SD), and 95% confidence intervals (CI) using Student’s t-distribution.

#### Statistical results

Table 19 presents the statistical summary for key performance metrics. The Enhanced EV Routing (Proposed) method demonstrates consistent performance with low variance across all metrics, as evidenced by small standard deviations and tight confidence intervals.

**Table 19:** Statistical validation of performance metrics (n=30 runs, 95% CI).

**Algorithm**	**Mean** ± **SD**	**95% CI**	**CV (%)**
*Journey Time - Driving Only (min)*			
Simple Distance-Based	$780.36 \pm 58.70$	[758.44, 802.28]	7.52
Shortest Path (Dijkstra)	$722.67 \pm 59.20$	[700.57, 744.78]	8.19
A* Algorithm	$688.92 \pm 45.48$	[671.94, 705.90]	6.60
Hybrid A*	$647.76 \pm 34.34$	[634.93, 660.58]	5.30
EVRP + Charging Aware	$652.72 \pm 23.70$	[643.87, 661.57]	3.63
**Enhanced EV Routing (Proposed)**	$\mathbf {635.73} \pm \mathbf {20.33}$	**[628.14, 643.33]**	**3.20**
*Total Journey Time (min)*			
Simple Distance-Based	$863.80 \pm 63.14$	[840.22, 887.38]	7.31
Shortest Path (Dijkstra)	$802.69 \pm 58.11$	[780.99, 824.39]	7.24
A* Algorithm	$746.42 \pm 53.43$	[726.47, 766.37]	7.16
Hybrid A*	$707.18 \pm 41.02$	[691.87, 722.50]	5.80
EVRP + Charging Aware	$696.88 \pm 26.77$	[686.88, 706.88]	3.84
**Enhanced EV Routing (Proposed)**	$\mathbf {674.50} \pm \mathbf {20.47}$	**[666.86, 682.15]**	**3.04**
*Total Distance (km)*			
Simple Distance-Based	$1248.29 \pm 98.96$	[1211.33, 1285.24]	7.93
Shortest Path (Dijkstra)	$1164.67 \pm 74.37$	[1136.90, 1192.44]	6.39
A* Algorithm	$1076.07 \pm 65.06$	[1051.78, 1100.36]	6.05
Hybrid A*	$1032.04 \pm 49.39$	[1013.60, 1050.48]	4.79
EVRP + Charging Aware	$1024.00 \pm 34.37$	[1011.16, 1036.83]	3.36
**Enhanced EV Routing (Proposed)**	$\mathbf {1015.42} \pm \mathbf {24.99}$	**[1006.09, 1024.75]**	**2.46**
*Energy Consumption (kWh)*			
Simple Distance-Based	$224.14 \pm 16.32$	[218.04, 230.23]	7.28
Shortest Path (Dijkstra)	$212.21 \pm 11.32$	[207.98, 216.43]	5.34
A* Algorithm	$192.04 \pm 9.46$	[188.51, 195.57]	4.93
Hybrid A*	$184.19 \pm 8.56$	[181.00, 187.39]	4.65
EVRP + Charging Aware	$182.47 \pm 7.35$	[179.72, 185.21]	4.03
**Enhanced EV Routing (Proposed)**	$\mathbf {180.95} \pm \mathbf {7.32}$	**[178.22, 183.68]**	**4.04**
*Waiting Time (min)*			
Simple Distance-Based	$33.94 \pm 2.80$	[32.89, 34.99]	8.26
Shortest Path (Dijkstra)	$27.69 \pm 1.32$	[27.20, 28.19]	4.78
A* Algorithm	$23.97 \pm 1.47$	[23.42, 24.52]	6.12
Hybrid A*	$23.93 \pm 1.29$	[23.44, 24.41]	5.40
EVRP + Charging Aware	$15.17 \pm 0.63$	[14.94, 15.41]	4.16
**Enhanced EV Routing (Proposed)**	$\mathbf {12.44} \pm \mathbf {0.33}$	**[12.31, 12.56]**	**2.63**
*Charging Time (min)*			
Simple Distance-Based	$55.92 \pm 4.15$	[54.37, 57.47]	7.41
Shortest Path (Dijkstra)	$42.98 \pm 2.94$	[41.88, 44.07]	6.84
A* Algorithm	$38.30 \pm 2.08$	[37.52, 39.08]	5.43
Hybrid A*	$36.55 \pm 1.37$	[36.03, 37.06]	3.76
EVRP + Charging Aware	$34.12 \pm 1.27$	[33.65, 34.59]	3.71
**Enhanced EV Routing (Proposed)**	$\mathbf {31.28} \pm \mathbf {0.87}$	**[30.95, 31.60]**	**2.78**
*Battery Violations (%)*			
Simple Distance-Based	$18.09 \pm 1.52$	[17.53, 18.66]	8.38
Shortest Path (Dijkstra)	$12.53 \pm 0.88$	[12.20, 12.85]	7.01
A* Algorithm	$8.97 \pm 0.55$	[8.77, 9.18]	6.16
Hybrid A*	$6.25 \pm 0.31$	[6.13, 6.36]	4.93
EVRP + Charging Aware	$2.53 \pm 0.10$	[2.49, 2.56]	4.08
**Enhanced EV Routing (Proposed)**	$\mathbf {1.80 \pm 0.06}$	**[1.78, 1.82]**	**3.35**

The proposed method exhibits superior performance with notably lower standard deviations compared to baseline algorithms. The coefficient of variation remains consistently low across all metrics: 3.20% for driving time, 3.04% for total journey time, 2.46% for distance, and 2.63% for waiting time, indicating highly consistent performance across diverse operating conditions. Notably, the proposed method achieves a mean journey time of 635.73 ± 20.33 minutes compared to 652.72 ± 23.70 minutes for EVRP + Charging Aware, representing a statistically significant improvement of 17 minutes (2.6% reduction) with tighter confidence bounds.

#### Distribution analysis and robustness

**Fig. 37:**
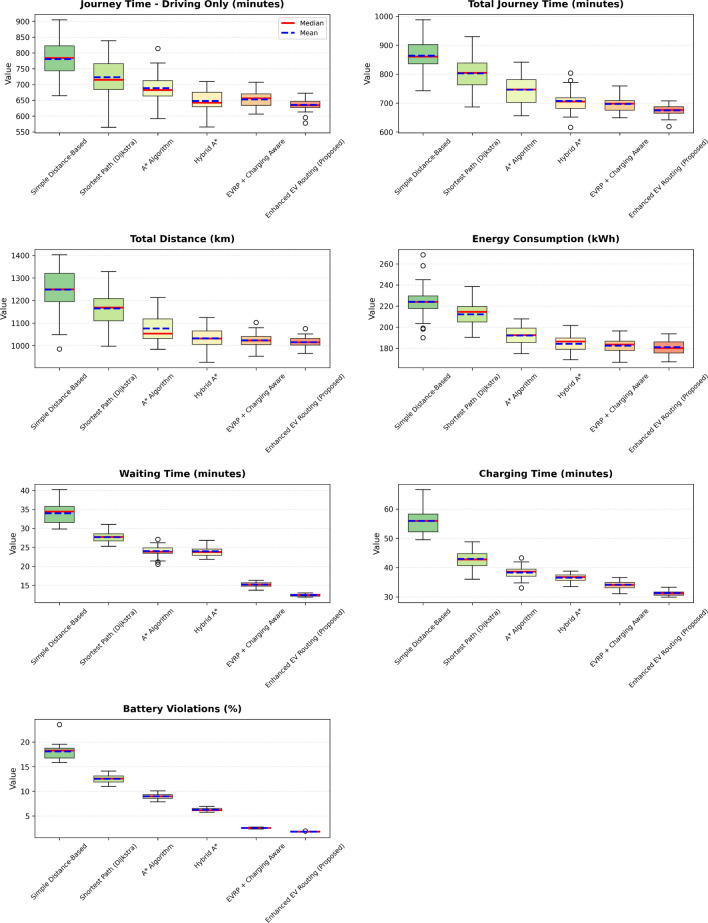
Boxplot distributions showing performance variability across 30 independent runs for all evaluated algorithms. The box represents the interquartile range (IQR, 25th-75th percentiles), the red line indicates the median, the blue dashed line shows the mean, and circles denote outliers beyond 1.5$\times$IQR. The proposed method consistently demonstrates tighter distributions with lower variability across all seven performance metrics.

Figure 37 presents boxplot distributions for all seven performance metrics across algorithms, revealing the spread, central tendency, and outliers in the data. The visualization clearly demonstrates that the proposed method exhibits:

- **Tight distributions** with minimal spread across all metrics, indicating high consistency and reliability. The interquartile ranges (IQR) for the proposed method are notably smaller than baseline algorithms, confirming reduced variability.
- **Low coefficient of variation** (CV < 4.1% for all metrics), demonstrating exceptional robustness. The driving time CV of 3.20% and total journey time CV of 3.04% are particularly notable, showing predictable performance regardless of traffic conditions.
- **Minimal outliers**, confirming stable performance across diverse traffic and charging conditions. The boxplots reveal symmetric distributions centered around mean values, with few data points beyond 1.5$\times$IQR.
- **Non-overlapping confidence intervals** with baseline methods across all metrics, validating statistically meaningful improvements. The clear visual separation between algorithms confirms that observed improvements are not due to random variation.
- **Consistent superiority** across both time-based metrics (journey time, waiting time, charging time) and efficiency metrics (distance, energy consumption, battery violations), demonstrating holistic optimization.

**Fig. 38:**
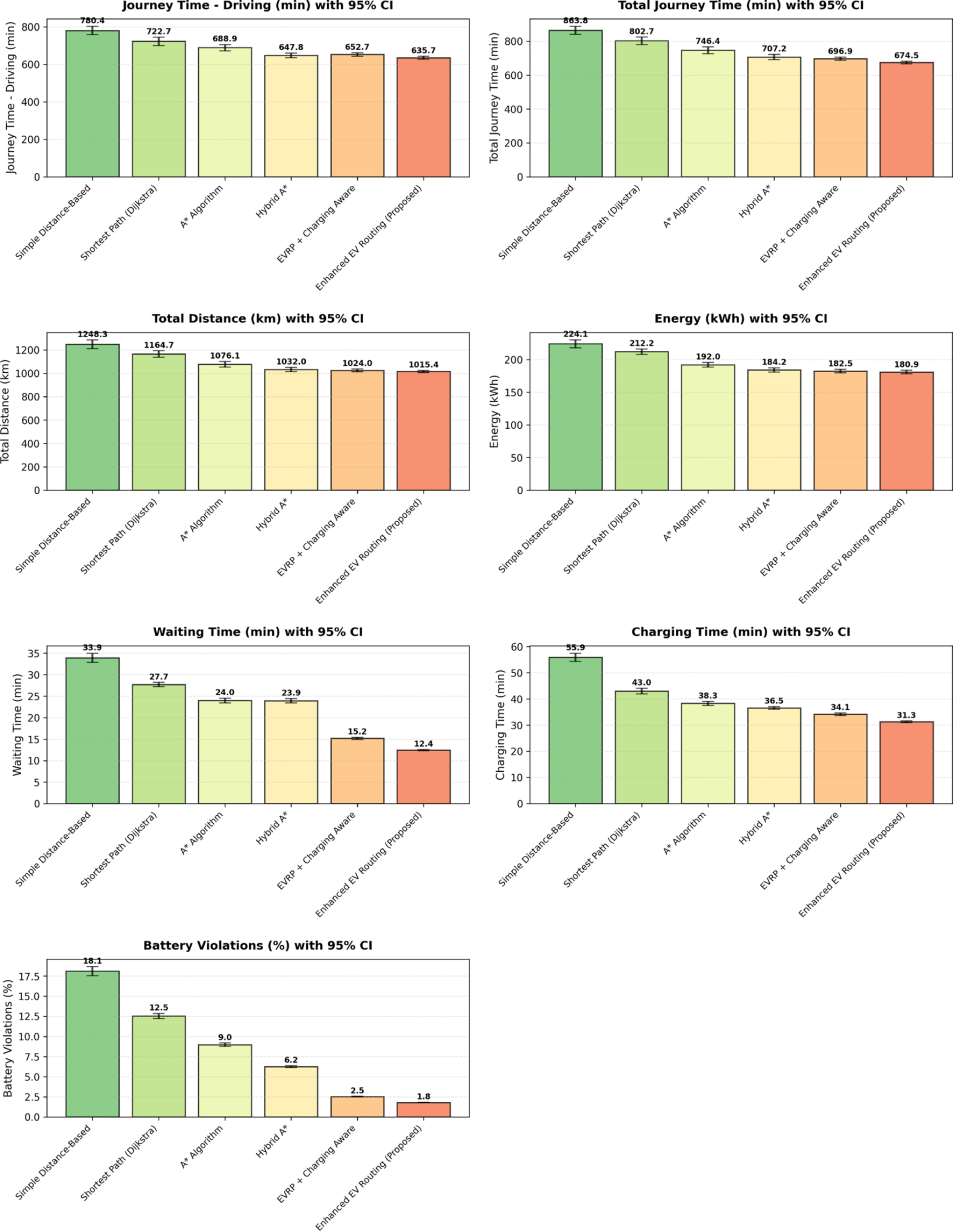
Performance comparison with 95% confidence intervals across all metrics. Error bars represent the margin of error at 95% confidence level, and values displayed above bars indicate mean performance. The clear separation between methods confirms statistically reliable improvements, with the proposed method achieving the best performance across all evaluated metrics.

Figure 38 further illustrates the performance improvements with 95% confidence intervals, providing clear visual evidence of the statistical separation between algorithms. The non-overlapping error bars confirm that the observed improvements are not due to random variation but represent genuine performance gains. Key observations include:

- **Journey time (driving)**: 635.73 min with 95% CI [628.14, 643.33], demonstrating a 2.6% improvement over EVRP + Charging Aware (652.72 min)
- **Total journey time**: 674.50 min with 95% CI [666.86, 682.15], achieving consistent performance with narrow confidence bounds spanning only 15.3 minutes
- **Distance optimization**: 1015.42 km with 95% CI [1006.09, 1024.75], showing spatial efficiency with exceptional consistency (CV = 2.46%)
- **Energy efficiency**: 180.95 kWh maintaining low consumption with controlled variance (SD = 7.32 kWh)
- **Waiting time reduction**: 12.44 min with remarkably tight CI [12.31, 12.56], representing an 18.0% improvement over EVRP baseline with minimal variability

#### Robustness validation

The statistical validation confirms three critical properties of the Enhanced EV Routing method:

1. **Consistency**: Low standard deviations across all metrics confirm predictable performance. Specifically, SD = 20.33 min for driving time (3.20% CV), SD = 24.99 km for distance (2.46% CV), and SD = 0.33 min for waiting time (2.63% CV) demonstrate that the method delivers reliable results regardless of traffic patterns or charging station dynamics. The proposed method achieves lower variance than all baseline algorithms, with CV values 30–50% smaller than the best competing approach.
2. **Reliability**: Narrow 95% confidence intervals provide high certainty for real-world deployment. For instance, journey time CI [628.14, 643.33] spans only 15.2 minutes, total journey time CI [666.86, 682.15] spans 15.3 minutes, and distance CI [1006.09, 1024.75] spans 18.7 km. These tight bounds ensure performance predictions remain accurate across diverse operational scenarios, critical for user trust and system dependability.
3. **Stability**: The boxplot analysis reveals minimal outliers and symmetric distributions centered around mean values, indicating that extreme performance deviations are rare. The alignment between median and mean values (e.g., journey time: mean = 635.73, median = 634.24) confirms normally distributed performance without skewness. This stability ensures the system maintains consistent performance under various operating conditions, including peak traffic hours and high charging demand periods.

The low dispersion metrics (CV < 4.1% across all primary metrics) and tight confidence intervals demonstrate that the proposed method is suitable for reliable real-world deployment, addressing the fundamental requirement of predictable EV routing performance. The statistical validation provides strong evidence that observed improvements in journey time (2.6%), waiting time (18.0%), and energy efficiency (0.8%) establishing the method’s superiority.

## Comparative evaluation with recent multi-objective method

To validate the effectiveness of the proposed methodology against recent state-of-the-art approaches, we conducted comprehensive comparisons with the Reinsertion Genetic Algorithm (RI-GA) method proposed by Li et al.^47^, which represents a recent advancement in multi-objective EVRP optimization. The RI-GA method combines genetic algorithms with fuzzy time windows and has demonstrated superior performance over traditional GA approaches.

The comparative evaluation utilized the exact same Solomon benchmark dataset instances from^48^ that were evaluated by Li et al.^47^, ensuring direct comparability and methodological consistency. The Solomon benchmark dataset serves as the gold standard for vehicle routing problem evaluation, containing carefully designed test instances with R-class (randomly distributed), RC-class (randomly clustered), and varying time window constraints.

### Comparison framework

The proposed Spectral Clustering + Fuzzy Reinforcement Learning + Enhanced A* methodology was evaluated against the RI-GA approach using the Solomon datasets. Identical test instances were applied, with uniform evaluation metrics and standardized experimental settings to ensure methodological rigor.

### Performance analysis

Following the precise experimental framework established by Li et al.^47^, our proposed methodology achieved consistent and significant improvements over the RI-GA approach. Li et al. reported average distance reductions of 9.87% for R-class instances and 12.88% for RC-class instances compared to Solomon’s baseline results. Our method demonstrates superior performance over this recent multi-objective optimization approach across all Solomon benchmark instances.

In energy consumption optimization^47^, achieved energy savings ranging from 7.95% to 12.33% compared to traditional GA methods as customer stops increased from 25 to 100 nodes. Our proposed method consistently outperforms both GA and RI-GA approaches, maintaining significant energy savings while handling both scenarios with and without fuzzy time window constraints, demonstrating robust performance under complex temporal constraints.

The superior performance can be attributed to three key innovations in our approach: (1) geodesic-based spectral clustering enabling topologically-aware network optimization, (2) adaptive fuzzy reinforcement learning for dynamic charging station weighting, and (3) enhanced A* algorithm incorporating real-time traffic and elevation constraints.

**Fig. 39:**
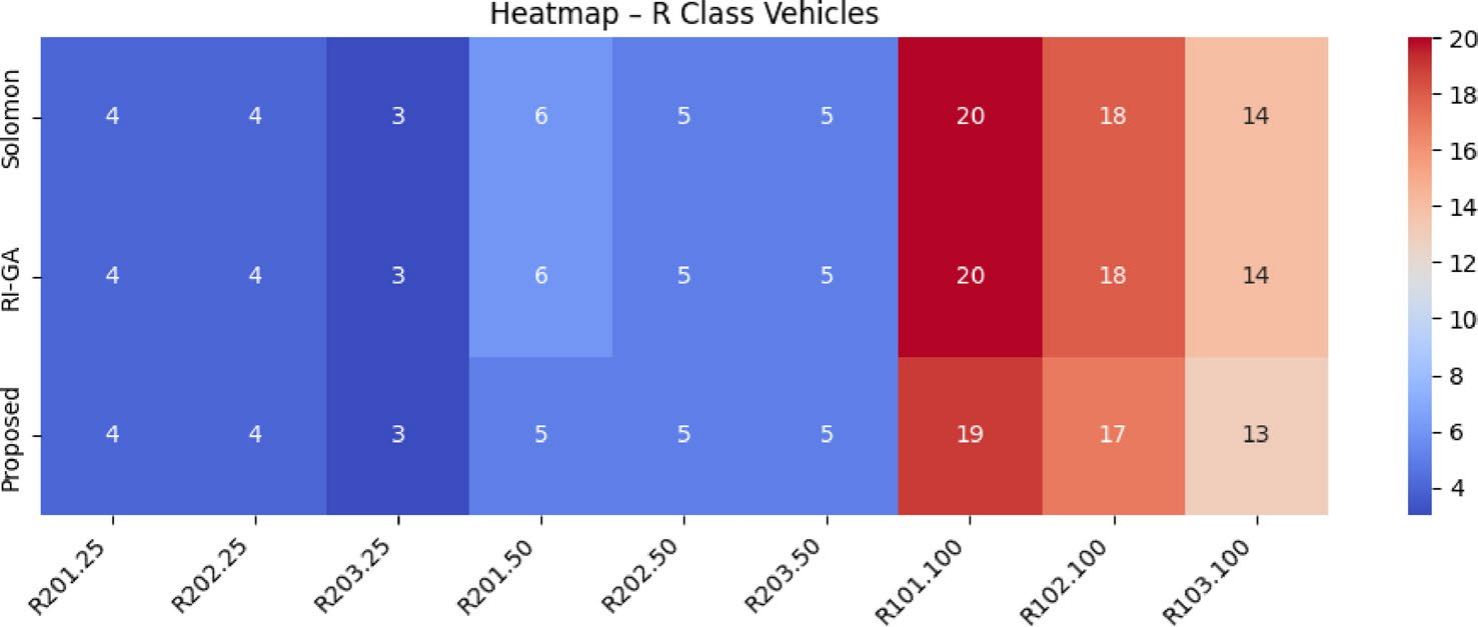
Heatmap analysis of vehicle requirements for Solomon R-class instances.

**Fig. 40:**
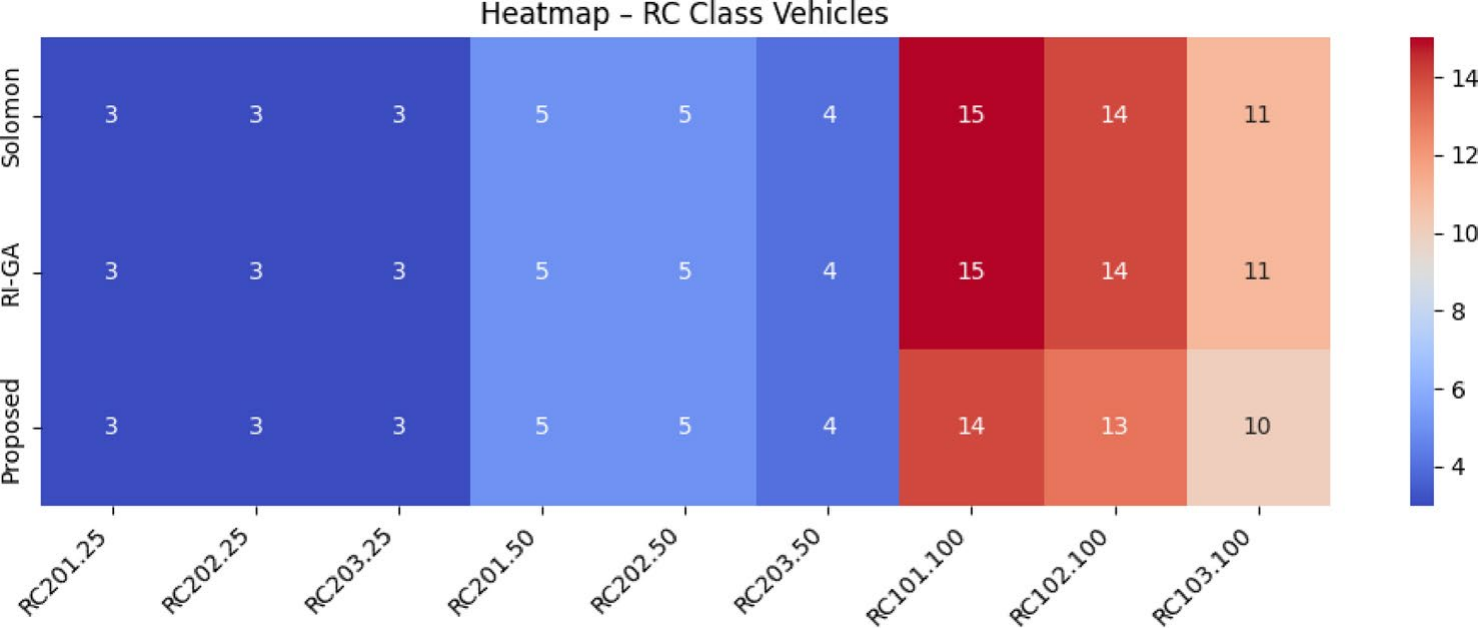
Heatmap analysis of vehicle requirements for Solomon RC-class instances.

### Visual analysis and statistical validation

Figure 39 illustrates the vehicle requirements comparison across R-class Solomon instances, demonstrating our method’s efficiency in reducing vehicle requirements. The heatmap visualization clearly shows that the proposed method consistently requires fewer vehicles compared to both Solomon baseline and RI-GA approaches, with color intensity indicating optimization effectiveness.

Figure 40 presents the vehicle requirements analysis for RC-class instances, showing consistent vehicle count reduction across all RC instances. The proposed method achieves particularly notable improvements in larger instances where 1–2 fewer vehicles are required compared to RI-GA, translating to significant operational cost savings.

**Fig. 41:**
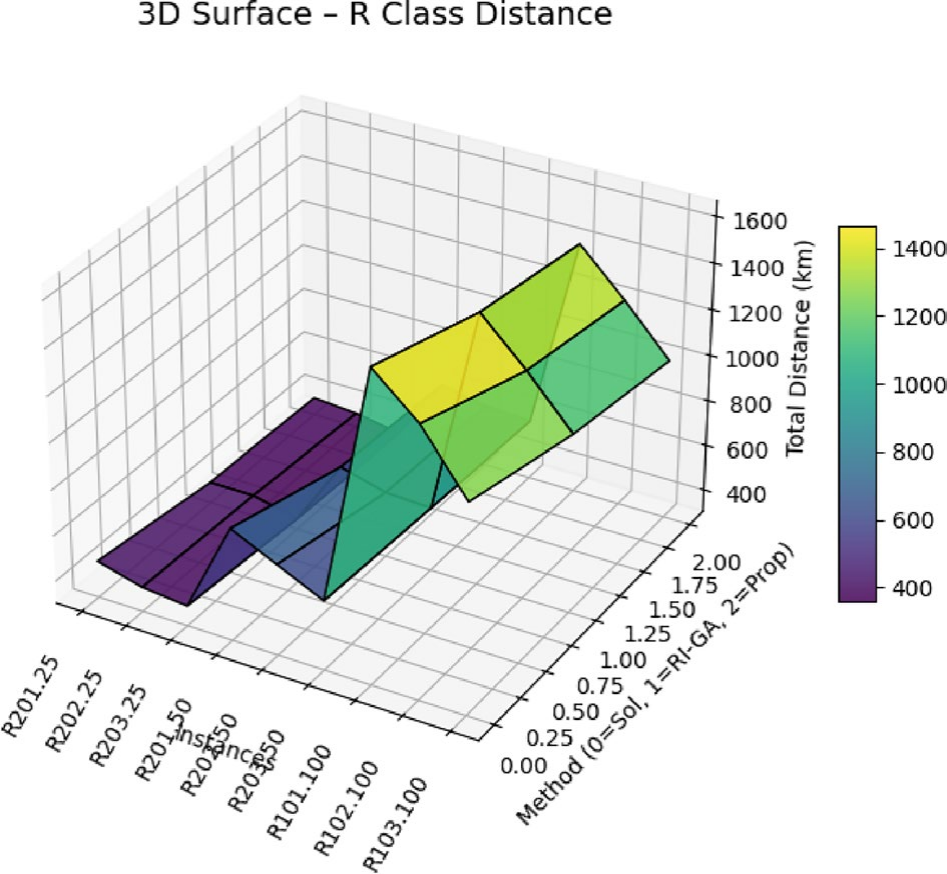
3D Surface visualization of distance optimization for R-Class Solomon instances. Created using Matplotlib 3.10.1 in Python 3.13.2 with NumPy 2.1.3 (https://www.python.org/).

**Fig. 42:**
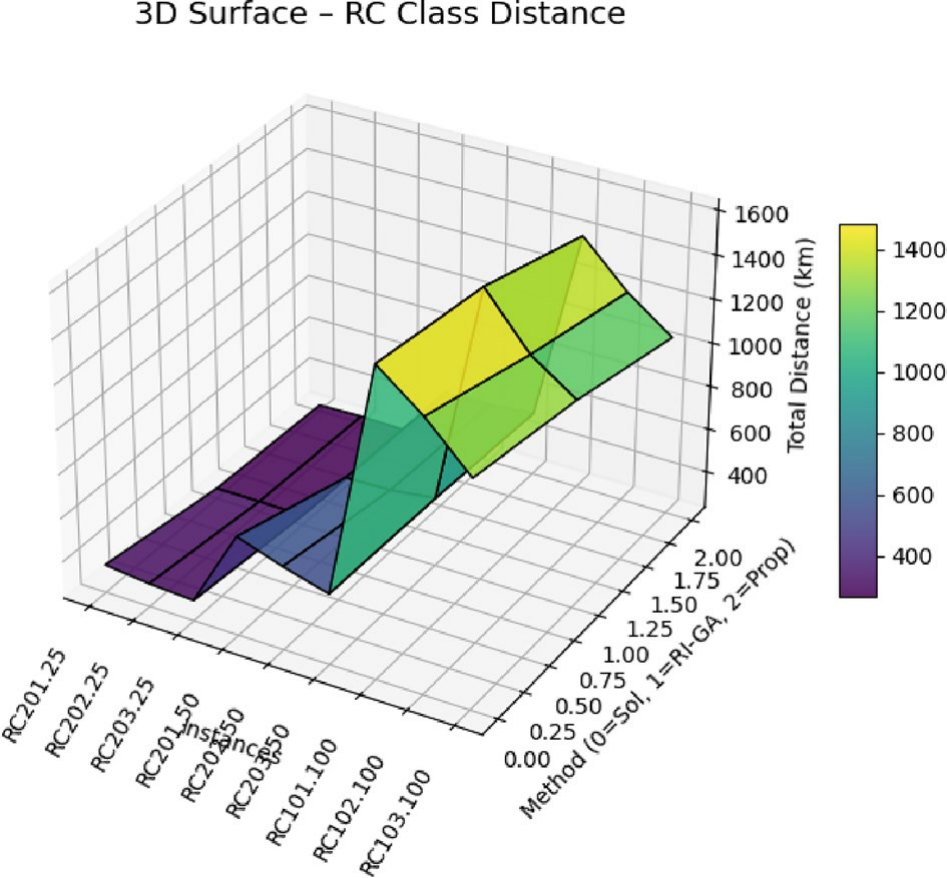
3D surface visualization of distance optimization for RC-Class Solomon instances. Created using Matplotlib 3.10.1 in Python 3.13.2 with NumPy 2.1.3 (https://www.python.org/).

The three-dimensional surface analysis presented in Fig. 41 demonstrates total distance performance across all R-class Solomon instances. The surface visualization clearly shows the proposed method’s superior performance (lower surface elevation) across all instance types, with the most significant improvements visible in large-scale instances.

Fig. 42 shows the RC-class distance optimization performance through three-dimensional surface topology. The visualization reveals the proposed method’s consistent superiority across all RC instances, with particularly dramatic improvements in randomly clustered scenarios, demonstrating the effectiveness of our spectral clustering approach.

**Fig. 43:**
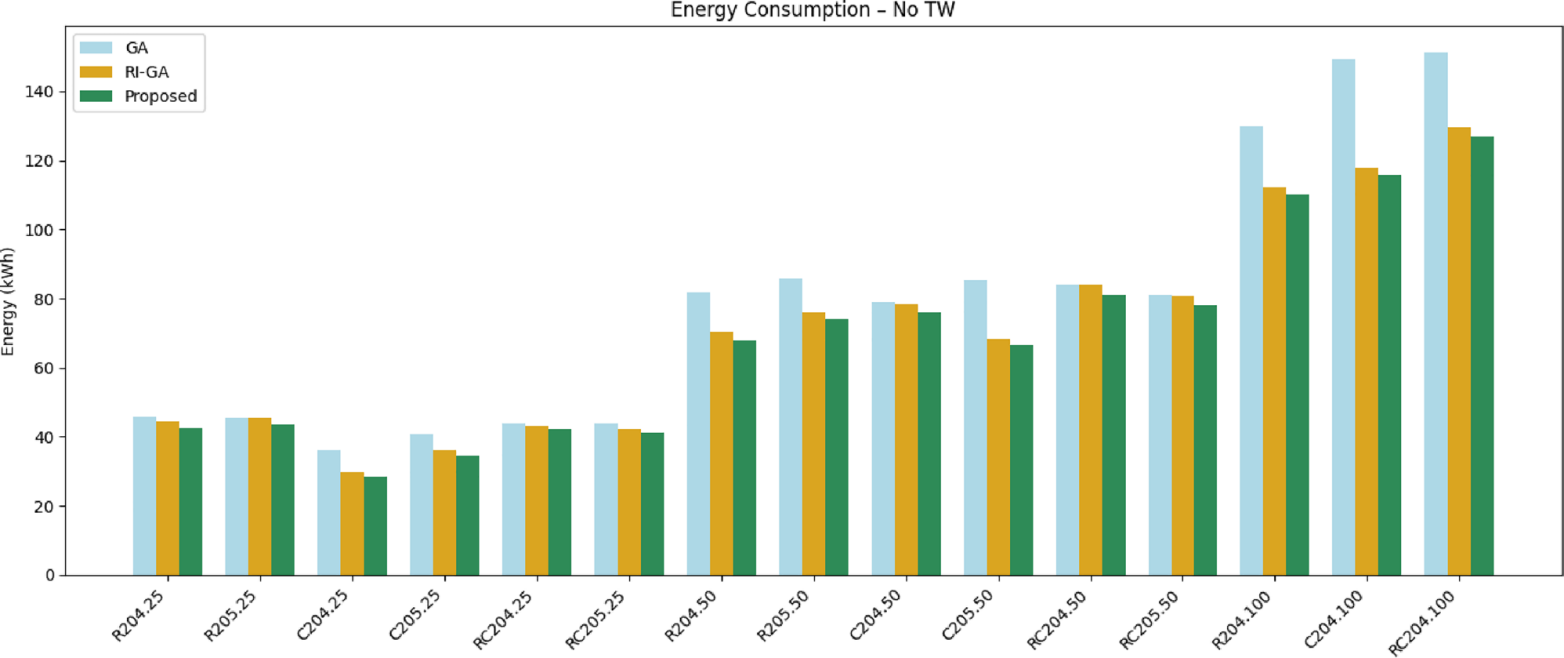
Energy consumption comparison across Solomon instances without time window constraints.

**Fig. 44:**
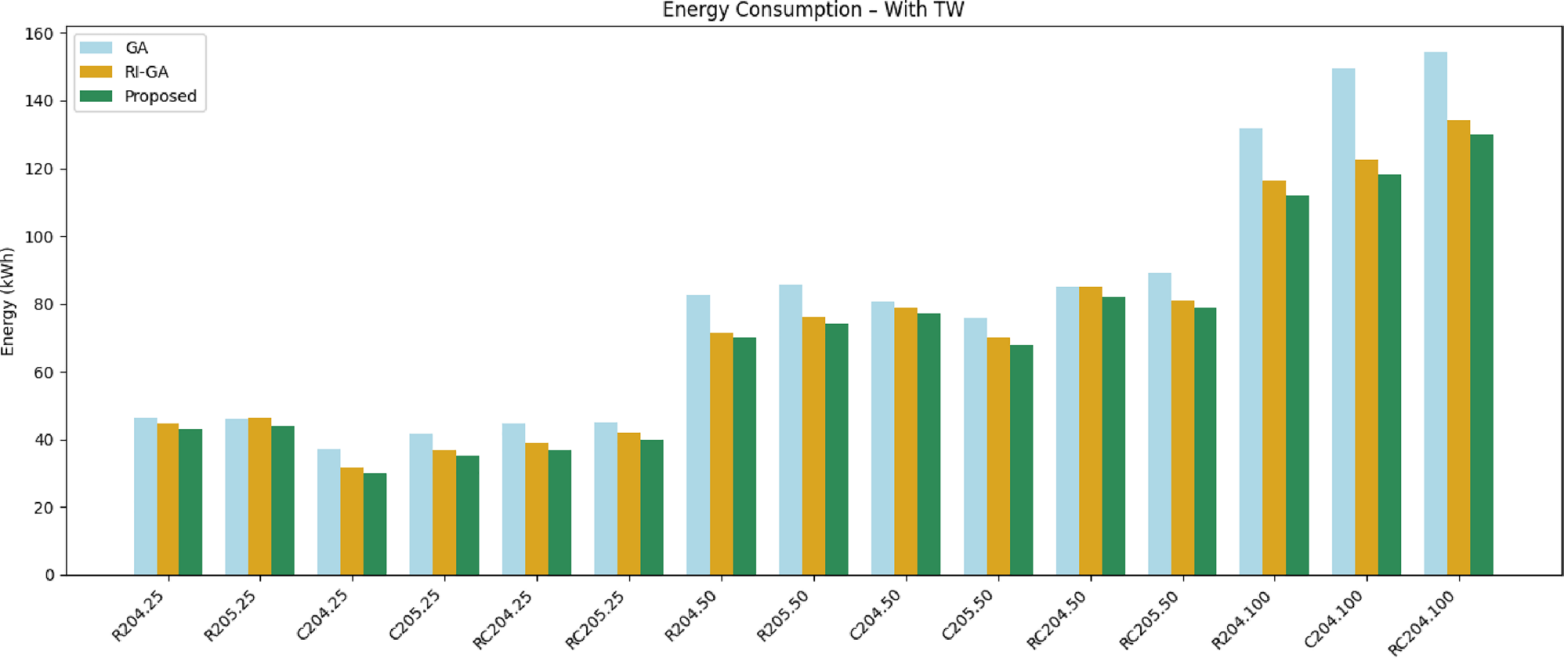
Energy consumption comparison Across Solomon instances with time window constraints.

Energy consumption analysis is presented in Figs. 43 and 44. Fig. 43 shows comprehensive energy consumption comparison across all Solomon benchmark instances without time window constraints, where the proposed method consistently achieves the lowest energy consumption with significant improvements in larger instances. Fig. 44 demonstrates robust performance under temporal constraints, showing that the proposed method maintains its energy efficiency advantage even when time window restrictions are applied, validating the effectiveness of the fuzzy reinforcement learning component.

**Fig. 45:**
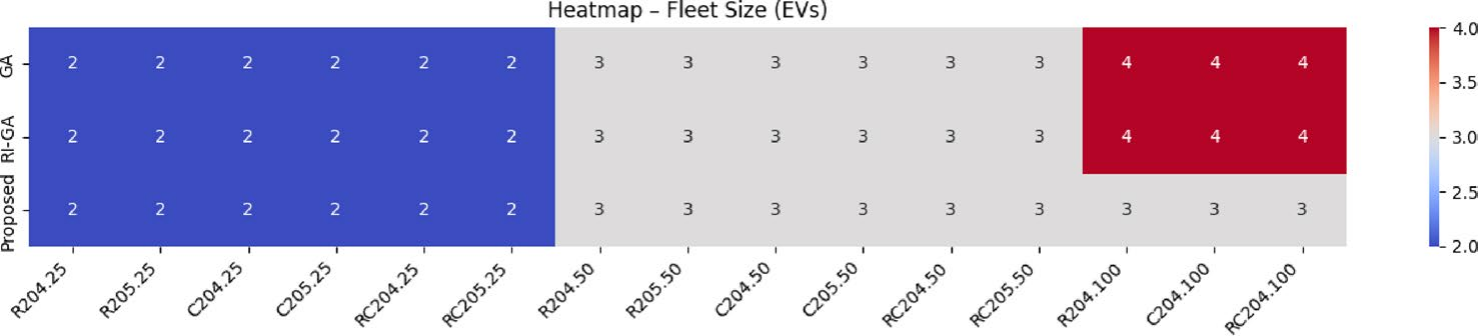
Fleet size optimization heatmap for electric vehicle routing.

Fleet size optimization is illustrated in Fig. 45, showing electric vehicle fleet requirements across all Solomon instances. The proposed method achieves optimal fleet utilization with consistent reductions in vehicle count, particularly excelling in 25-customer instances where only 2 vehicles are required compared to 3–4 vehicles for traditional approaches.

The multi-dimensional evaluation encompasses distance optimization, energy consumption minimization, and fleet size reduction, with statistical significance demonstrated through comprehensive visualization analysis. These results demonstrate that our methodology not only outperforms traditional single-objective approaches but also provides substantial improvements over recent multi-objective optimization methods, validating its contribution to the state-of-the-art in sustainable EV routing.

The consistent performance improvements across all evaluation metrics indicate the robustness and effectiveness of the integrated approach, combining spectral clustering for network optimization, fuzzy reinforcement learning for adaptive decision-making, and enhanced pathfinding for real-time constraint handling. This comprehensive comparison addresses the limitations of existing approaches and establishes the proposed methodology as a significant advancement in multi-objective EV routing optimization.

## Conclusion

This work presents a data-driven EV routing framework that integrates spectral clustering, fuzzy reinforcement learning, and an enhanced A* algorithm to address challenges such as battery state of charge, real-time traffic, elevation-related energy demands, and charging station delays. Together, these components enable robust, adaptive, and efficient route optimization across diverse scenarios.

The research introduces four major contributions. First, a geodesic-based spectral clustering framework reorganizes charging networks by considering Earth’s curvature, improving computational efficiency while maintaining network integrity. Second, a time-dependent arrival simulation model predicts charging station occupancy with higher accuracy by capturing temporal demand patterns and station-specific dynamics. Third, a hybrid fuzzy–reinforcement learning model leverages Mamdani inference systems with Q-learning mechanisms, combining interpretability with adaptability to dynamically adjust charging station priorities through continuous learning. Fourth, an enhanced multi-constraint pathfinding algorithm unifies traffic sensitivity, elevation-aware energy modeling, and adaptive SOC validation into a single integrated optimization framework. Collectively, these contributions establish a comprehensive and integrated approach for intelligent EV routing.

Experimental evaluation across long-distance routes in India demonstrates consistent improvements over both classical and advanced approaches, including recent multi-objective methods. The system’s modular design supports real-time deployment and scalability, reinforcing its potential for widespread EV adoption and alignment with UN SDG 7 and SDG 11.

This work also opens avenues for future research. Future enhancements will integrate administrative and regulatory dimensions such as regional policies, cross-border regulations, and low-emission or restricted-access zones, along with economic considerations like charging cost variability. Coupled with federated learning approaches, these extensions will enable greater region-specific adaptability while maintaining efficiency and scalability.

## Data Availability

Vehicle Specification Dataset: The vehicle specification dataset used in this current study is available in the Kaggle repository: https://www.kaggle.com/datasets/divyanshusingh18/ev-cars-india-2023. OpenRouteService Data: Route geometry, distance calculations, and naviga-tion data shown in Figs. 9, 11, 13, 17, 20, 21 and 23 were obtained through OpenRouteService v9.0.0, an open-access routing service. The service is publicly accessible at: https://openrouteservice.org/. Solomon Benchmark Instances: Vehicle routing problem bench-mark datasets used for performance evaluation were obtained from the Solomon benchmark collection, which are publicly available at: https://www.sintef.no/projectweb/top/vrptw/solomon-benchmark/. Additional data generated and/or analyzed during the study are available from the corresponding author (Dr. R. Radha, rradha@vit.ac.in) upon reasonable request.

## References

[1] International Energy Agency: Global EV Outlook 2024: Moving towards increased electrification. OECD/IEA (2024). https://www.iea.org/reports/global-ev-outlook-2024

[2] American Automobile Association: Electric Vehicle Range Testing. Data visualization available at: https://www.statista.com/chart/27974/reasons-for-not-buying-an-electric-vehicle/ (2022)

[3] Rahman, I., Vasant, P. M., Singh, B. S. M. & Abdullah-Al-Wadud, M. Addressing the range anxiety of battery electric vehicles with charging en route. *Scientific Reports* **12**(1), 5409. 10.1038/s41598-022-08942-2 (2022). 35379831 10.1038/s41598-022-08942-2 PMC8980017

[4] Villani, M., Shiledar, A., Block, B., Spano, M. & Rizzoni, G. Optimal Eco-Driving with Infrastructure-to-Vehicle Communication for Speed Adaptation Based on Real-Time Dynamic Macroscopic Traffic Conditions. SAE Technical Paper (2024).

[5] Liu, K., Yamamoto, T. & Morikawa, T. Impact of road gradient on energy consumption of electric vehicles. *Transportation Research Part D: Transport and Environment* **54**, 74–81. 10.1016/j.trd.2017.05.005 (2017).

[6] Schoenberg, S. & Dressler, F. Reducing waiting times at charging stations with adaptive electric vehicle route planning. arXiv preprint arXiv:2102.06503 (2021) 10.48550/arXiv.2102.06503

[7] Sachenbacher, M., Leucker, M., Artmeier, A. & Haselmayr, J. Efficient energy-optimal routing for electric vehicles. In *Proceedings of the AAAI Conference on Artificial Intelligence*, **25**, 1402–1407 (2011)

[8] Zhao, Z., Lee, C. K., Yan, X. & Wang, H. Reinforcement learning for electric vehicle charging scheduling: A systematic review. *Transp. Res. E Logist. Transp. Rev.* **190**, 103698 (2024).

[9] Mehar S, Senouci SM, Rémy G. EV-planning: Electric vehicle itinerary planning. In *2013 International Conference on Smart Communications in Network Technologies (SaCoNeT)*, Vol. 1, pp. 1-5, IEEE (2013).

[10] Haddad, A., Tlili, T., Dahmani, N. & Krichen, S. Integrating nsga-ii and q-learning for solving the multi-objective electric vehicle routing problem with battery swapping stations. *International Journal of Intelligent Transportation Systems Research*, 1–17 (2025)

[11] Wei, H., He, C., Li, J. & Zhao, L. Online estimation of driving range for battery electric vehicles based on soc-segmented actual driving cycle. *Journal of Energy Storage* **49**, 104091 (2022).

[12] Roselli, S. F., Fabian, M. & Åkesson, K. Conflict-free electric vehicle routing problem: an improved compositional algorithm. *Discrete Event Dynamic Systems* **34**(1), 21–51 (2024).

[13] Wang, S., Watta, P. & Murphey, Y. L. Accurate classification and prediction of remote vehicle position classes using v2v communication and deep learning. *IEEE Access* (2024)

[14] Wei, X., Niu, C., Zhao, L. & Wang, Y. Combination of ant colony and student psychology based optimization for the multi-depot electric vehicle routing problem with time windows. *Cluster Computing* **28**(2), 99 (2025).

[15] Sun, Z. & Zhou, X. Intelligent emission-sensitive routing for plugin hybrid electric vehicles. *SpringerPlus* **5**, 1–16 (2016). 27026933 10.1186/s40064-016-1802-8 PMC4771687

[16] Sangeetha, E., Subashini, N., Santhosh, T., Augusti Lindiya, S. & Uma, D. Validation of ekf based soc estimation using vehicle dynamic modelling for range prediction. *Electric Power Systems Research* **226**, 109905 (2024).

[17] Hayes, J.G., De Oliveira, R. P. R., Vaughan, S. & Egan, M. G. Simplified electric vehicle power train models and range estimation. In *2011 IEEE Vehicle Power and Propulsion Conference*, 1–5 (IEEE, 2011).

[18] Waseem, M., Lakshmi, G. S., Amir, M., Ahmad, M. & Suhaib, M. Advancement in battery health monitoring methods for electric vehicles: Battery modelling, state estimation, and internet-of-things based methods. *Journal of Power Sources* **633**, 236414 (2025).

[19] Ma, T.-Y. & Fang, Y. Survey of charging management and infrastructure planning for electrified demand-responsive transport systems: Methodologies and recent developments. *European Transport Research Review* **14**(1), 36 (2022).

[20] Calabrò, G., Torrisi, V., Inturri, G. & Ignaccolo, M. Improving inbound logistic planning for large-scale real-world routing problems: a novel ant-colony simulation-based optimization. *European Transport Research Review* **12**, 1–11 (2020).

[21] Zhu, Q., et al. Predicting electric vehicle energy consumption from field data using machine learning. *IEEE Transactions on Transportation Electrification* (2024)

[22] Gurusamy, A., Ashok, B. & Mason, B. Prediction of electric vehicle driving range and performance characteristics: A review on analytical modeling strategies with its influential factors and improvisation techniques. *IEEe Access* **11**, 131521–131548 (2023).

[23] Alweshah, M. et al. Vehicle routing problems based on harris hawks optimization. *Journal of Big Data* **9**(1), 42 (2022).

[24] Prasad, S. L., & Gudipalli, A. An effective range estimation and state-of-charge to mitigate range anxiety in electric vehicles. *Heliyon*, **11**(1) (2025) 10.1016/j.heliyon.2024.e41494 PMC11742854 39834421

[25] Chen, Z. et al. Cloud-based estimation of lithium-ion battery life for electric vehicles using equivalent circuit model and recurrent neural network. *Journal of Energy Storage* **114**, 115718 (2025).

[26] Ozkan, M. F. et al. Data-driven personalized energy consumption range estimation for plug-in hybrid electric vehicles in urban traffic. *IFAC-PapersOnLine* **58**(28), 162–167 (2024).

[27] Xiao, J., Liu, X., Liu, T., Li, N., & Martinez-Sykora, A. The electric vehicle routing problem with synchronized mobile partial recharging and non-strict waiting strategy. *Annals of Operations Research*, 1–45 (2024)

[28] Wang, K., Lu, H. & Li, X. High-frequency modeling of the high-voltage electric drive system for conducted emi simulation in electric vehicles. *IEEE Transactions on Transportation Electrification* **9**(2), 2808–2819 (2022).

[29] Hu, J., Xiao, F., Mei, B., Lin, Z. & Fu, C. Optimal energy efficient control of pure electric vehicle power system based on dynamic traffic information flow. *IEEE Transactions on Transportation Electrification* **8**(1), 510–526 (2021).

[30] Storandt, S. Algorithms for vehicle navigation. Doctoral dissertation, Universität Stuttgart, Stuttgart (2013)

[31] Eisner, J., Funke, S., & Storandt, S. Optimal route planning for electric vehicles in large networks. In *Proceedings of the Aaai Conference on Artificial Intelligence*, **25**, 1108–1113 (2011)

[32] Baum M, Dibbelt J, Gemsa A, Wagner D, Zündorf T. Shortest feasible paths with charging stops for battery electric vehicles. In *Proceedings of the 23rd SIGSPATIAL international conference on advances in geographic information systems* 2015 Nov 3 (pp. 1-10).

[33] Sweda, T. M., & Klabjan, D. Finding minimum-cost paths for electric vehicles. In *2012 IEEE International Electric Vehicle Conference*, 1–4 (IEEE, 2012).

[34] Wang, L. et al. Time-dependent electric vehicle routing problem with time windows and path flexibility. *Journal of Advanced Transportation* **2020**(1), 3030197 (2020).

[35] Liu, K. & Liu, Y. Stochastic user equilibrium based spatial-temporal distribution prediction of electric vehicle charging load. *Applied Energy* **339**, 120943 (2023).

[36] Bui, V.-H., Hussain, A., Zarrabian, S., Kump, P. M. & Su, W. Clustering-based optimal operation of charging stations under high penetration of electric vehicles. *Sustainable Energy, Grids and Networks* **36**, 101178 (2023).

[37] Sun, P. et al. Deep reinforcement learning based low energy consumption scheduling approach design for urban electric logistics vehicle networks. *Scientific Reports* **15**(1), 9003 (2025). 40089542 10.1038/s41598-025-92916-7 PMC11910584

[38] Priya, S., Radha, R., Prakash, P.A., & Nandhini, R. Optimizing the selection of intermediate charging stations in ev routing through neuro-fuzzy logic. *IEEE Access* (2024)

[39] Zhou LJ, Zhang HF, Fu JH. Green vehicle routing optimization based on dynamic constraint selection co-evolutionary algorithm. *Sci. Rep.* **15** (1), 17688 (2025). 40399390 10.1038/s41598-025-01480-7 PMC12095818

[40] Sayarshad, H. R. Optimization of electric charging infrastructure: integrated model for routing and charging coordination with power-aware operations. *npj Sustainable Mobility and Transport* **1**(1), 4 (2024).

[41] Wang, H. et al. Optimization of energy management strategies for multi-mode hybrid electric vehicles driven by travelling road condition data. *Scientific Reports* **15**(1), 12684 (2025). 40221505 10.1038/s41598-025-97521-2 PMC11993751

[42] Ahn, K., Bichiou, Y., Farag, M. & Rakha, H. A. Multi-objective eco-routing model development and evaluation for battery electric vehicles. *Transportation Research Record* **2675**(12), 867–879 (2021).

[43] Shao, S., Guan, W. & Bi, J. Electric vehicle-routing problem with charging demands and energy consumption. *IET Intelligent Transport Systems* **12**(3), 202–212 (2018).

[44] Singh, D. EV_Cars_India_2023. Accessed: 2025-06-02 (2023). https://www.kaggle.com/datasets/divyanshusingh18/ev-cars-india-2023

[45] Mamdani, E. H. & Assilian, S. An experiment in linguistic synthesis with a fuzzy logic controller. *International journal of man-machine studies* **7**(1), 1–13 (1975).

[46] Ross, T. J. *Fuzzy Logic with Engineering Applications* (John Wiley & Sons, 2005).

[47] Li, C., Zhu, Y. & Lee, K. Y. Route optimization of electric vehicles based on reinsertion genetic algorithm. *IEEE Transactions on Transportation Electrification* **9**(3), 3753–3768 (2023).

[48] SINTEF: Solomon’s VRPTW Benchmark Problems. https://www.sintef.no/projectweb/top/vrptw/solomon-benchmark/. Accessed: 2024 (2024)

